# ­­U.S. Medical Eligibility Criteria for Contraceptive Use, 2024

**DOI:** 10.15585/mmwr.rr7304a1

**Published:** 2024-08-08

**Authors:** Antoinette T. Nguyen, Kathryn M. Curtis, Naomi K. Tepper, Katherine Kortsmit, Anna W. Brittain, Emily M. Snyder, Megan A. Cohen, Lauren B. Zapata, Maura K. Whiteman, Contributors, Courtney Baker, Divya Dethier, Sophia Garbarino, Heather Gold, Emma Halper, Nathalie Kapp, Gopika Krishna, Marielle Meurice, Stephanie Ramer, Jessica Rodenhizer, Nisha Verma, Steffanie Wright

**Affiliations:** 1 Division of Reproductive Health, National Center for Chronic Disease Prevention and Health Promotion, CDC, Atlanta, Georgia; University of Texas Southwestern Medical Center, Dallas, Texas; University of Hawaii, Honolulu, Hawaii; Emory University, Atlanta, Georgia; Emory University, Atlanta, Georgia; Emory University, Atlanta, Georgia; International Planned Parenthood Federation, London, England; Columbia University, New York, New York; University of California-San Diego, San Diego, California; CDC, Atlanta, Georgia; CDC, Atlanta, Georgia; Emory University, Atlanta, Georgia; Harvard University, Boston, Massachusetts

## Abstract

*The 2024* U.S. Medical Eligibility Criteria for Contraceptive Use *(U.S. MEC) comprises recommendations for the use of specific contraceptive methods by persons who have certain characteristics or medical conditions. These recommendations for health care providers were updated by CDC after review of the scientific evidence and a meeting with national experts in Atlanta, Georgia, during January 25–27, 2023. The information in this report replaces the 2016 U.S. MEC* (CDC. U.S. Medical Eligibility Criteria for Contraceptive Use, 2016. MMWR 2016:65[No. RR-3]:1–103). *Notable updates include 1) the addition of recommendations for persons with chronic kidney disease; 2) revisions to the recommendations for persons with certain characteristics or medical conditions (i.e., breastfeeding, postpartum, postabortion, obesity, surgery, deep venous thrombosis or pulmonary embolism with or without anticoagulant therapy, thrombophilia, superficial venous thrombosis, valvular heart disease, peripartum cardiomyopathy, systemic lupus erythematosus, high risk for HIV infection, cirrhosis, liver tumor, sickle cell disease, solid organ transplantation, and drug interactions with antiretrovirals used for prevention or treatment of HIV infection); and 3) inclusion of new contraceptive methods, including new doses or formulations of combined oral contraceptives, contraceptive patches, vaginal rings, progestin-only pills, levonorgestrel intrauterine devices, and vaginal pH modulator. The recommendations in this report are intended to serve as a source of evidence-based clinical practice guidance for health care providers. The goals of these recommendations are to remove unnecessary medical barriers to accessing and using contraception and to support the provision of person-centered contraceptive counseling and services in a noncoercive manner. Health care providers should always consider the individual clinical circumstances of each person seeking contraceptive services. This report is not intended to be a substitute for professional medical advice for individual patients; when needed, patients should seek advice from their health care providers about contraceptive use.*

## Introduction

*U.S. Medical Eligibility Criteria for Contraceptive Use, 2024* (U.S. MEC) provides recommendations for health care providers for safe use of contraceptive methods for persons who have certain characteristics or medical conditions within the framework of removing unnecessary medical barriers to accessing and using contraception. U.S. MEC is a companion document to *U.S. Selected Practice Recommendations for Contraceptive Use, 2024* (U.S. SPR) (*1*), which provides recommendations for health care providers that address provision of contraceptive methods and management of side effects and issues related to contraceptive method use (*2*). Both U.S. MEC and U.S. SPR were adapted from global guidance developed by the World Health Organization (WHO) (*3*,*4*). WHO intended for the global guidance to be used by local or national policymakers, family planning program managers, and the scientific community as a reference when they develop family planning guidance at the country or program level (*3*). CDC first published U.S. MEC in 2010, after a formal process during 2008–2010 to adapt the global guidance for use in the United States, which included rigorous identification and critical appraisal of the scientific evidence through systematic reviews and input from national experts on how to translate that evidence into recommendations for U.S. health care providers (*5*); a subsequent update was published in 2016 (*6*).

U.S. MEC and U.S. SPR recommendations are components of quality contraceptive services and can be used in conjunction with other guidance documents such as *Providing Quality Family Planning Services: Recommendations of CDC and the U.S. Office of Population Affairs*, which provides recommendations for the content and delivery of services related to preventing or for achieving pregnancy (*7*–*9*). Evidence-based guidance can support health care providers when providing person-centered counseling and contraceptive services, including assisting persons in selecting and using contraceptive methods safely and effectively.

Equitable access to the full range of contraceptive methods for all those seeking care is an essential component of high-quality sexual and reproductive health care. Contraceptive services should be offered in a noncoercive manner that supports a person’s values, goals, and reproductive autonomy through a shared decision-making process with health care providers (*10*–*14*). Because of the history of and ongoing forced sterilization and reproductive coercion in the United States among persons of racial and ethnic minority groups, persons with disabilities, and other groups that have been marginalized, it is important that persons can select the method that best meets their needs to promote reproductive autonomy (*10*–*12*).

This report replaces the 2016 version of U.S. MEC (*6*) with new and revised recommendations, on the basis of new evidence and input from experts. This updated document uses gender-inclusive language throughout. However, when summarizing published evidence that describes study populations by specific genders, the wording of the primary studies has been maintained for accuracy. A summary of new and revised recommendations from the 2016 U.S. MEC is provided (Appendix A). Notable updates include

- addition of recommendations for persons with chronic kidney disease, specifically those with nephrotic syndrome, those receiving hemodialysis, and those receiving peritoneal dialysis;
- revisions to recommendations for persons with certain characteristics or medical conditions (i.e., breastfeeding, postpartum, postabortion, obesity, surgery, history of deep venous thrombosis or pulmonary embolism with or without anticoagulant therapy, thrombophilia, superficial venous thrombosis, valvular heart disease, peripartum cardiomyopathy, systemic lupus erythematosus, cirrhosis, liver tumor, sickle cell disease, and solid organ transplantation);
- revisions to recommendations for persons at high risk for HIV infection (this recommendation was developed and published in 2020) (*15*);
- revisions to recommendations for drug interactions with antiretrovirals to include prevention in addition to treatment for HIV infection (this recommendation was developed and published in 2020) (*15*); and
- inclusion of additional contraceptive methods, including new doses or formulations of combined oral contraceptives (COCs), contraceptive patches, vaginal rings, progestin-only pills (POPs), levonorgestrel intrauterine devices (LNG-IUDs), and vaginal pH modulator.

U.S. MEC recommendations are meant to serve as a source of evidence-based clinical guidance for health care providers and can support the provision of person-centered contraceptive counseling and services in a noncoercive manner. Health care providers should always consider the individual clinical circumstances of each person seeking contraceptive services. This report is not intended to be a substitute for professional medical advice for individual patients; when needed, patients should seek advice from their health care providers about contraceptive use.

## Methods

Since publication of the 2016 U.S. MEC, CDC has monitored the literature for new evidence relevant to the recommendations through the WHO/CDC Continuous Identification of Research Evidence (CIRE) system (*16*). This system identifies new evidence as it is published and allows WHO and CDC to update systematic reviews and facilitate updates to recommendations as new evidence warrants. Automated searches are run in PubMed weekly, and the results are reviewed. Abstracts that meet specific criteria are added to the web-based CIRE system, which facilitates coordination and peer review of systematic reviews for both WHO and CDC. For this update, CDC reviewed all existing recommendations in the 2016 U.S. MEC for new evidence identified by CIRE that had the potential to lead to a changed recommendation. To obtain comments from the public about revisions to CDC’s contraception recommendations (U.S. MEC and U.S. SPR), CDC published a notice in the Federal Register (86 FR 46703) on August 19, 2021, requesting public comment on content to consider for revision or addition to the recommendations and how to improve the implementation of the guidance documents (*17*). The comment period closed on October 18, 2021. CDC received 46 submissions from the general public, including private persons, professional organizations, academic institutions, and industry. CDC reviewed each of the submissions and carefully considered them when revising the recommendations.

During January 21, 25, and 26, 2022, CDC held virtual scoping meetings that included 27 participants with expertise in contraception, adolescent health, and thrombosis, as well as representatives from partner organizations, to solicit their individual input on the scope for updating both the 2016 U.S. MEC and 2016 U.S. SPR. The 27 invited participants represented various types of health care providers and health care provider organizations. Lists of participants and potential conflicts of interests are provided at the end of this report. Meeting participants discussed topics to be addressed in the update of U.S. MEC on the basis of the presentation of new evidence published since 2016 (identified through the CIRE system), submissions received through the Federal Register notice, and feedback CDC received from other sources (e.g., health care providers and others through e-mail, public inquiry, and questions received at conferences). CDC identified multiple topics to consider when updating the guidance, including revision of existing recommendations for certain characteristics or medical conditions (postpartum, postabortion, obesity, anticoagulant therapy, known thrombogenic mutations, viral hepatitis, cirrhosis, liver tumors, sickle cell disease, and solid organ transplantation), addition of recommendations for new characteristics or medical conditions (chronic kidney disease and antiphospholipid syndrome), and addition of recommendations for new contraceptive methods (including new formulations of COCs, contraceptive patches, vaginal rings, POPs, LNG-IUDs, and vaginal pH modulator). CDC determined that all other recommendations in the 2016 U.S. MEC were up to date and consistent with the existing body of evidence for that recommendation.

In preparation for a subsequent expert meeting held during January 25–27, 2023, to review the scientific evidence for potential recommendations, CDC staff members and other invited authors conducted systematic reviews for each of the topics being considered. The purpose of these systematic reviews was to identify direct and indirect evidence about the safety of contraceptive method use by persons with selected characteristics or medical conditions (e.g., risk for disease progression or other adverse health effects in persons with chronic kidney disease who use combined hormonal contraceptives [CHCs]). Person-centered outcomes that might represent contraceptive users’ values and preferences (e.g., method continuation and patient satisfaction) were considered where relevant and available for each of the systematic reviews. Preferred Reporting Items for Systematic Reviews and Meta-Analyses (PRISMA) guidelines were followed for reporting systematic reviews (*18*). The Grading of Recommendations Assessment, Development and Evaluation (GRADE) approach was used to assess the certainty of the evidence (*19*,*20*). Certainty of evidence was rated as high, moderate, low, or very low depending on criteria including study design, risk for bias, indirectness, imprecision, and inconsistency. Outcomes evaluated in randomized clinical trials (RCTs) are considered to have high certainty of evidence and those in observational studies to have low certainty; these ratings are adjusted according to the previously mentioned criteria. When direct evidence was limited or not available, indirect evidence (e.g., evidence on proxy outcomes or among healthy persons) and theoretical issues were considered. Reviews are referenced and cited throughout this report; the full reviews will be submitted to peer-reviewed journals and will contain the details of each review, including the systematic review question, literature search protocol (registered in https://www.crd.york.ac.uk/PROSPERO), inclusion and exclusion criteria, evidence tables, and quality assessments. Brief summaries of the evidence and GRADE tables are included (Supplementary Appendix, https://stacks.cdc.gov/view/cdc/156516). CDC staff members continued to monitor new evidence identified through the CIRE system during the preparation for the January 2023 meeting.

In addition to the preparation of the systematic reviews, CDC included patient perspectives in the guideline update process to better consider how the resulting updated recommendations could meet patient preferences and needs. Consideration of patient perspectives can center discussions on the evidence in a person-centered care model, can support inclusion of patient perspectives along with provider perspectives on the evidence, and has the potential to shape recommendations (*14*,*21*,*22*). In November and December 2022, listening sessions were held with a different group of 18 participants, representing themselves or patient advocacy organizations, who provided perspectives from patient populations such as youths; lesbian, gay, bisexual, transgender, queer, and intersex (LGBTQI+) persons; persons with disabilities; and persons with chronic medical conditions. The goal of the listening sessions was to gather insights about participants’ experiences, values, preferences, and information needs related to contraceptive choice and decision-making.

During January 25–27, 2023, in Atlanta, Georgia, CDC held a meeting with 40 participants who were invited to provide their individual perspectives on the scientific evidence presented and the implications for practice for U.S. MEC. Thirty-eight participants represented a wide range of expertise in contraception provision, research, and reproductive justice and included obstetricians and gynecologists, pediatricians, family physicians, internal medicine physicians, nurse practitioners, epidemiologists, and others with research and clinical practice expertise in contraceptive safety, effectiveness, and management. Two participants were patient representatives who provided their individual perspectives on the topics discussed throughout the meeting. Six additional participants with expertise relevant to specific topics on the meeting agenda provided information and participated in the discussion on their topic of expertise only (e.g., an expert in kidney disease was asked to provide general information about the condition and to assist in interpreting the evidence and any theoretical concerns on the use of contraceptive methods in persons with the condition). During the meeting, a summary of the information from the patient listening sessions was presented, and the two patient representatives presented information on their individual experiences and perspectives related to receipt of contraceptive services. The evidence from the systematic review for each topic was presented, including direct evidence and any indirect evidence or theoretical concerns. Meeting participants provided their individual perspectives on topics discussed throughout the meeting and on using the evidence to develop recommendations that would meet the needs of U.S. health care providers and the patients they serve. Participants also provided feedback on the certainty of evidence, the balance of benefits and harms, and values and preferences. Areas of research that need additional investigation also were considered during the meeting. Lists of participants and potential conflicts of interest are provided at the end of this report.

After the January 2023 meeting, CDC determined the recommendations in this report, taking into consideration the individual perspectives provided by the meeting participants. Feedback also was received from a group of four external reviewers, composed of health care providers and researchers who had not participated in the scoping or update meetings. These external reviewers were asked to provide comments on the accuracy, feasibility, and clarity of the recommendations.

## Keeping Guidance Up to Date

As with any evidence-based guidance document, a key challenge is keeping the recommendations up to date as new scientific evidence becomes available. Working with WHO, CDC uses the CIRE system to ensure that WHO and CDC guidance is based on the best available evidence and that a mechanism is in place to update guidance when new evidence becomes available (*16*). CDC will continue to work with WHO to identify and assess all new relevant evidence and determine whether changes in the recommendations are warranted. CDC will completely review U.S. MEC periodically. Updates to the guidance will published in CDC’s *Morbidity and Mortality Weekly Report* (*MMWR*) and posted on the CDC website (https://www.cdc.gov/contraception/hcp/contraceptive-guidance).

As part of the process to update these recommendations, CDC identifies gaps in the evidence for the recommendations considered. Evidence is often limited on the safety of contraceptive methods among persons with certain characteristics or medical conditions. Generalizability of the published evidence to all persons seeking contraceptive services presents a challenge because of biases about who might be included in studies on contraceptive safety. New, high-quality research on contraception that addresses priority research gaps inclusive of diverse populations can further strengthen these recommendations and improve clinical practice.

## How to Use This Document

The recommendations in this report are intended to help health care providers determine the safe use of contraceptive methods among persons with certain characteristics and medical conditions. Providers can use the information in these recommendations during contraceptive counseling with patients. The tables include recommendations for the use of contraceptive methods by persons with certain characteristics or medical conditions. Each condition is defined as representing either a person’s characteristics (e.g., age or postpartum status) or a known medical condition (e.g., diabetes or hypertension). The recommendations refer to contraceptive methods being used for contraceptive purposes; the recommendations do not consider the use of contraceptive methods for treatment of medical conditions because the eligibility criteria in these situations might differ. The conditions affecting eligibility for the use of each contraceptive method are classified into one of four categories (Box 1).

BOX 1 **Categories of medical eligibility criteria for contraceptive use**

U.S. MEC 1 = A condition for which there is no restriction for the use of the contraceptive method

U.S. MEC 2 = A condition for which the advantages of using the method generally outweigh the theoretical or proven risks

U.S. MEC 3 = A condition for which the theoretical or proven risks usually outweigh the advantages of using the method

U.S. MEC 4 = A condition that represents an unacceptable health risk if the contraceptive method is used

**Abbreviation:** U.S. MEC = *U.S. Medical Eligibility Criteria for Contraceptive Use*.

### Contraceptive Decision-Making

CDC acknowledges the paramount importance of personal autonomy in contraceptive decision-making. This is critically important because of the context of historical and ongoing contraceptive coercion and reproductive mistreatment in the United States, especially among communities that have been marginalized, including human rights violations such as forced sterilization and enrollment in contraceptive trials without informed consent (*10*–*12*). Coercive practices in the health care system can include provider bias for certain contraceptive methods over a patient’s reproductive goals and preferences, lack of person-centered counseling and support, and policies or incentives for uptake of certain contraceptive methods (*11*). For health care providers and the settings in which they work, it is important to acknowledge the structural systems that drive inequities (e.g., discrimination because of race, ethnicity, disability, sex, gender, and sexual orientation), work to mitigate harmful impacts, and recognize that provider bias (unconscious or explicit) might affect contraceptive counseling and provision of services (*12*). All persons seeking contraceptive care need access to appropriate counseling and services that support the person’s values, goals, and reproductive autonomy (*10*–*14*). Health care providers can support the contraceptive needs of all persons by using a person-centered framework and recognizing the many factors that influence individual decision-making about contraception (*10*,*12*,*14*).

The U.S. MEC and U.S. SPR recommendations can be used to support a person’s contraceptive decision-making (Box 2). Persons should have equitable access to the full range of contraceptive methods and be given the information they need for contraceptive decision-making in a noncoercive manner. Patient-centeredness has been defined by the Institute of Medicine as “providing care that is respectful of and responsive to individual patient preferences, needs, and values and ensuring that patient values guide all clinical decisions” (*23*). Shared decision-making and person-centered approaches to providing health care recognize the expertise of both the medical provider and the patient (*10*,*12*,*23*).

BOX 2 **Using the *U.S. Medical Eligibility Criteria for Contraceptive Use* and *U.S. Selected Practice Recommendations for Contraceptive Use* recommendations to support contraceptive decision-making**

- CDC acknowledges the paramount importance of personal autonomy in contraceptive decision-making.
- Persons should have equitable access to the full range of contraceptive methods.
- Contraceptive services should be offered in a noncoercive manner that honors a person’s values, goals, and reproductive autonomy.
- Shared decision-making and person-centered approaches recognize the expertise of both the health care provider and the person.
- A person-centered approach to contraceptive decision-making
  º prioritizes a person’s preferences and reproductive autonomy rather than a singular focus on pregnancy prevention,
  º respects the person as the main decision-maker in contraceptive decisions, and
  º includes respecting the decision not to use contraception or to discontinue contraceptive method use.
- U.S. MEC and U.S. SPR recommendations can be used by health care providers to support persons in contraceptive decision-making.
- U.S. MEC and U.S. SPR recommendations can be used by health care providers to remove unnecessary medical barriers to accessing and using contraception.

**Abbreviations:** U.S. MEC = *U.S. Medical Eligibility Criteria for Contraceptive Use*; U.S. SPR = *U.S. Selected Practice Recommendations for Contraceptive Use*.

Health care providers should always consider the individual clinical and social factors of each person seeking contraceptive services and discuss reproductive desires, expectations, preferences, and priorities regarding contraception. A person might consider and prioritize many elements when choosing an acceptable contraceptive method, such as safety, effectiveness (*24*), availability (including accessibility and affordability), side effects, user control, reversibility, and ease of removal or discontinuation. In addition, a person’s health risks associated with pregnancy and access to comprehensive health care services should be considered in these discussions. A person-centered approach to contraceptive decision-making prioritizes a person’s preferences and reproductive autonomy rather than a singular focus on pregnancy prevention and respects the person as the main decision-maker in contraceptive decisions, including the decision not to use contraception or to discontinue contraceptive method use (*12*,*25*). Voluntary informed choice of contraceptive methods is an essential guiding principle, and contraceptive counseling, where applicable, might be an important contributor to the successful use of contraceptive methods. Key resources provide additional information on person-centered contraceptive counseling and care (*7*,*10*,*12*,*26*).

### Using U.S. MEC Categories in Practice

Health care providers can use the eligibility categories when assessing the safety of contraceptive method use for persons with certain characteristics or medical conditions. Category 1 comprises conditions for which no restrictions exist for use of the contraceptive method. However, category 1 does not imply that the method is the most appropriate choice for a person, who might be prioritizing other factors when considering contraception. Classification of a method or condition as category 2 indicates the method generally can be used, with additional discussion about risks and benefits, and careful follow-up might be required. For a method or condition classified as category 3, use of that method usually is not recommended unless other more appropriate methods are not available or acceptable. The severity of the condition and the availability, practicality, and acceptability of alternative methods should be considered, and careful follow-up is required. Hence, provision of a contraceptive method to a person with a condition classified as category 3 requires careful clinical judgment and might warrant additional counseling, consultation, or follow-up. Category 4 comprises conditions that represent an unacceptable health risk if the method is used. For example, a person who smokes and is aged <35 years generally can use COCs (category 2). However, for a person aged ≥35 years who smokes <15 cigarettes per day, the use of COCs usually is not recommended unless other methods are not available or acceptable (category 3). A person aged ≥35 years who smokes ≥15 cigarettes per day should not use COCs because of unacceptable health risks, primarily the risk for myocardial infarction and stroke (category 4). The implementation of this clinical guidance might vary within different health systems, clinics, or settings. For example, in certain settings, category 3 might mean that a special consultation is warranted. Health departments and medical societies or organizations can provide information on implementation through additional guidance or clinical protocols.

The recommendations address medical eligibility criteria for the initiation and continued use of all contraceptive methods evaluated. The issue of medical eligibility criteria for continuation of a contraceptive method is clinically relevant whenever a medical condition develops or worsens during use of a contraceptive method. When the categories differ for initiation and continuation, these differences are noted. When different initiation and continuation recommendations are not given, the category is the same for initiation and continuation of use.

On the basis of this classification system, the eligibility criteria for initiating and continuing use of a specific contraceptive method are presented in tables (Appendices A, B, C, D, E, and J). In these tables, the first column indicates the condition. Multiple conditions are divided into subconditions to differentiate between varying condition types or severity. The next columns provide classifications of the condition for initiation, continuation, or both into categories 1, 2, 3, or 4 for specific contraceptive methods. For certain conditions, the last column further clarifies the numeric category in cases where the numeric classification does not adequately capture the recommendation. These clarifications are considered a necessary element of the recommendation. The last column also summarizes the evidence for the recommendation if evidence exists. The recommendations for which no evidence is cited might be based on information from sources other than systematic reviews and might take into account individual perspectives from either the WHO or U.S. expert meetings in which these recommendations were developed. For certain recommendations, comments in the third column can provide additional rationale or other information about the recommendation. Information provided along with the numeric recommendation (i.e., clarifications, evidence, and comments) is additional detail that providers can use as part of their counseling and referrals, as needed.

U.S. MEC recommendations comprise one aspect of contraceptive counseling. All persons should be counseled about the full range of contraceptive options for which they are medically eligible. Voluntary informed choice of contraceptive methods is an essential guiding principle of these recommendations, and person-centered contraceptive counseling can help to ensure a person’s contraceptive needs are met successfully.

### Recommendations for Use of Contraceptive Methods

The classifications for whether persons with certain characteristics or medical conditions can safely use specific contraceptive methods are provided for intrauterine devices (IUDs), including the copper IUD (Cu-IUD) and LNG-IUD (Appendix B); progestin-only contraceptives (POCs), including progestin-only implants, depot medroxyprogesterone acetate injections, and POPs (Appendix C); CHCs, including COCs, combined transdermal patches, and combined vaginal rings (Appendix D); barrier contraceptive methods, including external (male) and internal (female) condoms, spermicides and vaginal pH modulator, and diaphragm with spermicide or cervical cap with spermicide (Appendix E); fertility awareness–based methods (Appendix F); lactational amenorrhea method (Appendix G); coitus interruptus (Appendix H); permanent contraception, including tubal surgery and vasectomy (Appendix I); and emergency contraception, including emergency use of the Cu-IUD and emergency contraceptive pills (Appendix J). A table at the end of this report summarizes the classifications for the hormonal and intrauterine methods (Appendix K).

### Prevention of Sexually Transmitted Infections

All patients, regardless of contraceptive choice, should be counseled about the use of condoms and the risk for sexually transmitted infections (STIs), including HIV infection (*27*). Most contraceptive methods, such as hormonal methods, IUDs, and permanent contraception do not protect against STIs, including HIV infection. Consistent and correct use of external (male) latex condoms reduces the risk for STIs, including HIV infection (*27*). Although evidence is limited, use of internal (female) condoms can provide protection from acquisition and transmission of STIs (*27*). Patients also should be counseled that pre-exposure prophylaxis (PrEP), when taken as prescribed, is highly effective for preventing HIV infection (*28*). Additional information about prevention and treatment of STIs is available from CDC’s *Sexually Transmitted Infections Treatment Guidelines* (https://www.cdc.gov/std/treatment-guidelines/default.htm) (*27*), and information on PrEP for prevention of HIV infection is available from the U.S. Public Health Service’s *Preexposure Prophylaxis for the Prevention of HIV Infection in the United States — 2021 Update: A Clinical Practice Guideline* (https://www.cdc.gov/hiv/pdf/risk/prep/cdc-hiv-prep-guidelines-2021.pdf) (*28*).

### Pregnancy and Increased Health Risk

Discussion of health risks associated with pregnancy is an important aspect of contraceptive counseling. For persons with certain medical conditions, pregnancy poses increased health risks. Conditions included in U.S. MEC that are associated with increased risk for adverse health events as a result of pregnancy are identified throughout the document (Box 3). This is not a comprehensive list of all conditions that could lead to adverse events during pregnancy. Certain medical conditions included in U.S. MEC recommendations also are treated with teratogenic drugs, which could have adverse effects when used during pregnancy. When applying U.S. MEC classifications during person-centered counseling, health care providers should discuss the risks of a particular contraceptive method as well as the health risks associated with pregnancy. Even though permanent contraception and long-acting, reversible contraceptive methods are highly effective, persons should be provided with the full range of contraceptive options and supported in their autonomous decisions about pregnancy planning and contraceptive choices. Discussions about pregnancy should include reviewing access to comprehensive health care services and subspecialists for a high-risk pregnancy (*29*).

BOX 3 Conditions included in *U.S. Medical Eligibility Criteria for Contraceptive Use* associated with increased risk for adverse health events as a result of pregnancy*

Breast cancer

Chronic kidney disease: with current nephrotic syndrome, receiving hemodialysis, or receiving peritoneal dialysis

Complicated valvular heart disease

Cystic fibrosis

Decompensated cirrhosis

Deep venous thrombosis/pulmonary embolism

Diabetes: insulin dependent; with nephropathy, retinopathy, or neuropathy or other vascular disease; or of >20 years’ duration

Endometrial cancer

Epilepsy

Gestational trophoblastic disease

Hepatocellular adenoma and malignant liver tumors (hepatocellular carcinoma)

History of bariatric surgery within the past 2 years

HIV infection: not clinically well or not receiving antiretroviral therapy

Hypertension (systolic ≥160 mm Hg or diastolic ≥100 mm Hg)

Ischemic heart disease

Ovarian cancer

Peripartum cardiomyopathy

Schistosomiasis with fibrosis of the liver

Sickle cell disease

Solid organ transplantation within the past 2 years

Stroke

Systemic lupus erythematosus

Thrombophilia (e.g., factor V Leiden mutation; prothrombin gene mutation; protein S, protein C, and antithrombin deficiencies; or antiphospholipid syndrome)

Tuberculosis

* Even though permanent contraception and long-acting, reversible contraceptive methods are highly effective, persons should be provided with the full range of contraceptive options and supported in their autonomous decisions about pregnancy planning and contraceptive choices. Discussions about pregnancy should include reviewing access to comprehensive health care services and subspecialists for a high-risk pregnancy.

## Appendix A Summary of Changes from *U.S. Medical Eligibility Criteria for Contraceptive Use, 2016*

The classification additions, deletions, and modifications from the 2016 *U.S. Medical Eligibility Criteria for Contraceptive Use* (U.S. MEC) are summarized in this appendix (Box A1) (Tables A1, A2, and A3). For conditions for which classifications changed for one or more contraceptive methods or for which the condition description underwent a substantive modification, the changes or modifications are noted (Tables A1, A2, and A3). Conditions that do not appear in this table remain unchanged from the 2016 U.S. MEC.

BOX A1 Categories for classifying intrauterine devices and hormonal contraceptives U.S. MEC 1 = A condition for which there is no restriction for the use of the contraceptive method U.S. MEC 2 = A condition for which the advantages of using the method generally outweigh the theoretical or proven risks U.S. MEC 3 = A condition for which the theoretical or proven risks usually outweigh the advantages of using the method U.S. MEC 4 = A condition that represents an unacceptable health risk if the contraceptive method is used **Abbreviation:** U.S. MEC = *U.S. Medical Eligibility Criteria for Contraceptive Use*.

**TABLE A1:** Summary of changes in classifications for hormonal contraceptive methods and intrauterine devices from *U.S. Medical Eligibility Criteria for Contraceptive Use, 2016*

Condition	Cu-IUD		LNG-IUD				Implant	DMPA		POP	CHC	Clarification
**Breastfeeding**												
a. <21 days postpartum	—		—				2	2		2	4	Breastfeeding provides important health benefits for breastfeeding parent and infant. The U.S. Dietary Guidelines for Americans and American Academy of Pediatrics recommend that infants be exclusively breastfed for about the first 6 months with continued breastfeeding while introducing appropriate complementary foods for 1 year or longer (*1*) or up to age 2 years or longer (*2*).
b. 21 to <30 days postpartum												
i. With other risk factors for VTE (e.g., age ≥35 years, previous VTE, thrombophilia, immobility, transfusion at delivery, peripartum cardiomyopathy, BMI ≥30 kg/m^2^, postpartum hemorrhage, postcesarean delivery, preeclampsia, or smoking)	—		—				2	2		2	3	**CHC:** For persons with other risk factors for VTE, these risk factors might increase the classification to a category 4. **Breastfeeding:** Breastfeeding provides important health benefits for breastfeeding parent and infant. The U.S. Dietary Guidelines for Americans and American Academy of Pediatrics recommend that infants be exclusively breastfed for about the first 6 months with continued breastfeeding while introducing appropriate complementary foods for 1 year or longer (*1*) or up to age 2 years or longer (*2*).
ii. Without other risk factors for VTE	—		—				2	2		2	3	Breastfeeding provides important health benefits for breastfeeding parent and infant. The U.S. Dietary Guidelines for Americans and American Academy of Pediatrics recommend that infants be exclusively breastfed for about the first 6 months with continued breastfeeding while introducing appropriate complementary foods for 1 year or longer (*1*) or up to age 2 years or longer (*2*).
c. 30–42 days postpartum												
i. With other risk factors for VTE (e.g., age ≥35 years, previous VTE, thrombophilia, immobility, transfusion at delivery, peripartum cardiomyopathy, BMI ≥30 kg/m^2^, postpartum hemorrhage, postcesarean delivery, preeclampsia, or smoking)	—		—				1	2*		1	3	**CHC:** For persons with other risk factors for VTE, these risk factors might increase the classification to a category 4. **Breastfeeding:** Breastfeeding provides important health benefits for breastfeeding parent and infant. The U.S. Dietary Guidelines for Americans and American Academy of Pediatrics recommend that infants be exclusively breastfed for about the first 6 months with continued breastfeeding while introducing appropriate complementary foods for 1 year or longer (*1*) or up to age 2 years or longer (*2*).
ii. Without other risk factors for VTE	—		—				1	1		1	2	Breastfeeding provides important health benefits for breastfeeding parent and infant. The U.S. Dietary Guidelines for Americans and American Academy of Pediatrics recommend that infants be exclusively breastfed for about the first 6 months with continued breastfeeding while introducing appropriate complementary foods for 1 year or longer (*1*) or up to age 2 years or longer (*2*).
d. >42 days postpartum	—		—				1	1		1	2	Breastfeeding provides important health benefits for breastfeeding parent and infant. The U.S. Dietary Guidelines for Americans and American Academy of Pediatrics recommend that infants be exclusively breastfed for about the first 6 months with continued breastfeeding while introducing appropriate complementary foods for 1 year or longer (*1*) or up to age 2 years or longer (*2*).
**Postpartum **(nonbreastfeeding)												
a. <21 days postpartum	—		—				1	2*		1	4	—
b. 21–42 days postpartum												
i. With other risk factors for VTE (e.g., age ≥35 years, previous VTE, thrombophilia, immobility, transfusion at delivery, peripartum cardiomyopathy, BMI ≥30 kg/m^2^, postpartum hemorrhage, postcesarean delivery, preeclampsia, or smoking)	—		—				1	2*		1	3	**CHC:** For persons with other risk factors for VTE, these risk factors might increase the classification to a category 4.
ii. Without other risk factors for VTE	—		—				1	1		1	2	—
c. >42 days postpartum	—		—				1	1		1	1	—
**Postpartum** (including cesarean delivery, breastfeeding, or nonbreastfeeding)												
a. <10 minutes after delivery of the placenta	2*		2*				—	—		—	—	**IUD:** Postpartum placement of IUDs is safe and does not appear to increase health risks associated with IUD use such as infection. Higher rates of expulsion during the postpartum period should be considered as they relate to effectiveness, along with patient access to interval placement (i.e., not related to pregnancy) when expulsion rates are lower. **Breastfeeding:** Breastfeeding provides important health benefits for breastfeeding parent and infant. The U.S. Dietary Guidelines for Americans and American Academy of Pediatrics recommend that infants be exclusively breastfed for about the first 6 months with continued breastfeeding while introducing appropriate complementary foods for 1 year or longer (*1*) or up to age 2 years or longer (*2*).
b. 10 minutes after delivery of the placenta to <4 weeks	2		2				—	—		—	—	**IUD:** Postpartum placement of IUDs is safe and does not appear to increase health risks associated with IUD use such as infection. Higher rates of expulsion during the postpartum period should be considered as they relate to effectiveness, along with patient access to interval placement (i.e., not related to pregnancy) when expulsion rates are lower. **Breastfeeding:** Breastfeeding provides important health benefits for breastfeeding parent and infant. The U.S. Dietary Guidelines for Americans and American Academy of Pediatrics recommend that infants be exclusively breastfed for about the first 6 months with continued breastfeeding while introducing appropriate complementary foods for 1 year or longer (*1*) or up to age 2 years or longer (*2*).
c. ≥4 weeks	1		1				—	—		—	—	**IUD:** Postpartum placement of IUDs is safe and does not appear to increase health risks associated with IUD use such as infection. Higher rates of expulsion during the postpartum period should be considered as they relate to effectiveness, along with patient access to interval placement (i.e., not related to pregnancy) when expulsion rates are lower. **Breastfeeding:** Breastfeeding provides important health benefits for breastfeeding parent and infant. The U.S. Dietary Guidelines for Americans and American Academy of Pediatrics recommend that infants be exclusively breastfed for about the first 6 months with continued breastfeeding while introducing appropriate complementary foods for 1 year or longer (*1*) or up to age 2 years or longer (*2*).
d. Postpartum sepsis	4		4				—	—		—	—	—
**Postabortion **(spontaneous or induced)												
a. First trimester abortion												
i. Procedural (surgical)*	1		1				1	1		1	1	**IUD:** IUDs may be placed immediately after abortion completion. **POC:** POCs may be started immediately after abortion completion or at time of medication abortion initiation. **DMPA**: After a first trimester medication abortion that did not include mifepristone, there is no restriction for the use of DMPA (category 1). After a first trimester medication abortion that included mifepristone, there is no restriction for use of DMPA after abortion completion (category 1) and benefits generally outweigh risks with DMPA use immediately at time of medication abortion initiation (category 2). Concurrent administration of DMPA with mifepristone might slightly decrease medication abortion effectiveness and increase risk for ongoing pregnancy. Risk for ongoing pregnancy with concurrent administration of DMPA with mifepristone should be considered along with personal preference and access to follow-up abortion and contraceptive care.* **CHC:** CHCs may be started immediately after abortion completion or at time of medication abortion initiation.
ii. Medication*	1		1				1	1/2*		1	1	
iii. Spontaneous abortion with no intervention*	1		1				1	1		1	1	
b. Second trimester abortion												
i. Procedural (surgical)*	2		2				1	1		1	1	**IUD:** IUDs may be placed immediately after abortion completion. **POC:** POCs may be started immediately after abortion completion or at time of medication abortion initiation. **CHC:** CHCs may be started immediately after abortion completion or at time of medication abortion initiation.
ii. Medication*	2		2				1	1		1	1	
iii. Spontaneous abortion with no intervention*	2		2				1	1		1	1	
c. Immediate postseptic abortion	4		4				1	1		1	1	**POC:** POCs may be started immediately after abortion completion or at time of medication abortion initiation. **CHC:** CHCs may be started immediately after abortion completion or at time of medication abortion initiation.
**Obesity**												
a. BMI ≥30 kg/m^2^	1		1				1	1		1	2	**CHC:** Risk for thrombosis increases with multiple risk factors, such as obesity, older age (e.g., ≥40 years), diabetes, smoking, family history of thrombosis, and dyslipidemia. When a person has multiple risk factors, any of which alone would increase risk for thrombosis, use of CHCs might increase thrombosis risk to an unacceptable level. However, a simple addition of categories for multiple risk factors is not intended; for example, a combination of two category 2 risk factors might not necessarily warrant a higher category.*
b. Menarche to <18 years and BMI ≥30 kg/m^2^	1		1				1	2		1	2	**CHC:** Risk for thrombosis increases with multiple risk factors, such as obesity, older age (e.g., ≥40 years), diabetes, smoking, family history of thrombosis, and dyslipidemia. When a person has multiple risk factors, any of which alone would increase risk for thrombosis, use of CHCs might increase thrombosis risk to an unacceptable level. However, a simple addition of categories for multiple risk factors is not intended; for example, a combination of two category 2 risk factors might not necessarily warrant a higher category.*
**Surgery**												
a. Minor surgery without immobilization	1		1				1	1		1	1	—
b. Major surgery												
i. Without prolonged immobilization	1		1				1	1		1	2	—
ii. With prolonged immobilization	1		1*				1*	2		1*	4	—
**Deep venous thrombosis/ Pulmonary embolism** This condition is associated with increased risk for adverse health events as a result of pregnancy (Box 3).												
a. Current or history of DVT/PE, receiving anticoagulant therapy (therapeutic dose) (e.g., acute DVT/PE or long-term therapeutic dose)*	2		2				2	2		2	3*	**Cu-IUD:** Persons using anticoagulant therapy are at risk for gynecologic complications of therapy, such as heavy or prolonged bleeding. Cu-IUDs might worsen bleeding.* **LNG-IUD:** Persons using anticoagulant therapy are at risk for gynecologic complications of therapy, such as heavy or prolonged bleeding. LNG-IUDs can be of benefit in preventing or treating this complication. When a contraceptive method is used as a therapy, rather than solely to prevent pregnancy, the risk/benefit ratio might differ and should be considered on a case-by-case basis.* **POC:** Persons using anticoagulant therapy are at risk for gynecologic complications of therapy, such as heavy or prolonged bleeding and hemorrhagic ovarian cysts. POCs can be of benefit in preventing or treating these complications; benefits might vary by POC dose and formulation. When a contraceptive method is used as a therapy, rather than solely to prevent pregnancy, the risk/benefit ratio might differ and should be considered on a case-by-case basis.* **CHC:** Persons using anticoagulant therapy are at risk for gynecologic complications of therapy, such as heavy or prolonged bleeding and hemorrhagic ovarian cysts. CHCs can be of benefit in preventing or treating these complications. When a contraceptive method is used as a therapy, rather than solely to prevent pregnancy, the risk/benefit ratio might differ and should be considered on a case-by-case basis.* **CHC:** When a patient discontinues therapeutic dose of anticoagulant therapy, careful consideration should be given to transitioning from CHCs to a progestin-only or nonhormonal method, if acceptable to the patient.*
b. History of DVT/PE, receiving anticoagulant therapy (prophylactic dose)*												**Cu-IUD:** Persons using anticoagulant therapy are at risk for gynecologic complications of therapy, such as heavy or prolonged bleeding. Cu-IUDs might worsen bleeding.* **LNG-IUD:** Persons using anticoagulant therapy are at risk for gynecologic complications of therapy, such as heavy or prolonged bleeding. LNG-IUDs can be of benefit in preventing or treating this complication. When a contraceptive method is used as a therapy, rather than solely to prevent pregnancy, the risk/benefit ratio might differ and should be considered on a case-by-case basis.* **POC:** Persons using anticoagulant therapy are at risk for gynecologic complications of therapy, such as heavy or prolonged bleeding and hemorrhagic ovarian cysts. POCs can be of benefit in preventing or treating these complications; benefits might vary by POC dose and formulation. When a contraceptive method is used as a therapy, rather than solely to prevent pregnancy, the risk/benefit ratio might differ and should be considered on a case-by-case basis.* **CHC:** Persons using anticoagulant therapy are at risk for gynecologic complications of therapy, such as heavy or prolonged bleeding and hemorrhagic ovarian cysts. CHCs can be of benefit in preventing or treating these complications. When a contraceptive method is used as a therapy, rather than solely to prevent pregnancy, the risk/benefit ratio might differ and should be considered on a case-by-case basis.*
i. Higher risk for recurrent DVT/PE (one or more risk factors)*	2		2				2	3*		2	4	
• Thrombophilia (e.g., factor V Leiden mutation; prothrombin gene mutation; protein S, protein C, and antithrombin deficiencies; or antiphospholipid syndrome)* • Active cancer (metastatic, receiving therapy, or within 6 months after clinical remission), excluding nonmelanoma skin cancer* • History of recurrent DVT/PE*												
ii. Lower risk for recurrent DVT/PE (no risk factors)*	2		2				2	2		2	3	
c. History of DVT/PE, not receiving anticoagulant therapy*												
i. Higher risk for recurrent DVT/PE (one or more risk factors)*	1		2				2	3*		2	4	
• History of estrogen-associated DVT/PE • Pregnancy-associated DVT/PE* • Idiopathic DVT/PE* • Thrombophilia (e.g., factor V Leiden mutation; prothrombin gene mutation; protein S, protein C, or antithrombin deficiencies; or antiphospholipid syndrome)* • Active cancer (metastatic, receiving therapy, or within 6 months after clinical remission), excluding nonmelanoma skin cancer* • History of recurrent DVT/PE*												
ii. Lower risk for recurrent DVT/PE (no risk factors)*	1		2				2	2		2	3	
d. Family history (first-degree relatives)	1		1				1	1		1	2	
**Thrombophilia** (e.g., factor V Leiden mutation; prothrombin gene mutation; protein S, protein C, and antithrombin deficiencies; or antiphospholipid syndrome) This condition is associated with increased risk for adverse health events as a result of pregnancy (Box 3).	1		2				2	3*		2	4	Routine screening in the general population before contraceptive initiation is not recommended. If a person has current or history of DVT/PE, see recommendations for DVT/PE.* Classification of antiphospholipid syndrome includes presence of a clinical feature (e.g., thrombosis or obstetric morbidity) and persistently abnormal antiphospholipid antibody test on two or more occasions at least 12 weeks apart (*3*).*
**Superficial venous disorders**												
a. Varicose veins	1		1				1	1		1	1	—
b. Superficial venous thrombosis (acute or history)	1		1				1	2*		1	3	**CHC:** Superficial venous thrombosis might be associated with an increased risk for VTE. If a person has risk factors for concurrent DVT (e.g., thrombophilia or cancer) or has current or history of DVT, see recommendations for DVT/PE. Superficial venous thrombosis associated with a peripheral intravenous catheter is less likely to be associated with additional thrombosis and use of CHCs may be considered.
**Valvular heart disease** Complicated valvular heart disease is a condition associated with increased risk for adverse health events as a result of pregnancy (Box 3).												
a. Uncomplicated	1		1				1	1		1	2	**—**
b. Complicated (pulmonary hypertension, risk for atrial fibrillation, or history of subacute bacterial endocarditis)	1		1				1	2*		1	4	
**Peripartum cardiomyopathy** This condition is associated with increased risk for adverse health events as a result of pregnancy (Box 3).												
a. Normal or mildly impaired cardiac function (New York Heart Association Functional Class I or II: no limitation of activities or slight, mild limitation of activity) (*4*)												
i. <6 months	2		2				1	2*		1	4	—
ii. ≥6 months	2		2				1	2*		1	3	
b. Moderately or severely impaired cardiac function (New York Heart Association Functional Class III or IV: marked limitation of activity or should be at complete rest) (*4*)	2		2				2	3*		2	4	
**Chronic kidney disease*** This condition is associated with increased risk for adverse health events as a result of pregnancy (Box 3).	Initiation	Continuation	Initiation	Continuation			—					
a. Current nephrotic syndrome*	1*	1*	2*	2*			2*	3*		2* DRSP POP with known hyperkalemia: 4*	4*	**DRSP POP:** Persons with known hyperkalemia should not use DRSP POPs because of the risk for worsening hyperkalemia (category 4). For persons with CKD without known hyperkalemia (category 2), consider checking serum potassium level during first cycle of DRSP POPs.*
b. Hemodialysis*	1*	1*	2*	2*			2*	3*		2* DRSP POP with known hyperkalemia: 4*	4*	**DRSP POP:** Persons with known hyperkalemia should not use DRSP POPs because of the risk for worsening hyperkalemia (category 4). For persons with CKD without known hyperkalemia (category 2), consider checking serum potassium level during first cycle of DRSP POPs.*
c. Peritoneal dialysis*	2*	1*	2*	2*			2*	3*		2* DRSP POP with known hyperkalemia: 4*	4*	**DRSP POP:** Persons with known hyperkalemia should not use DRSP POPs because of the risk for worsening hyperkalemia (category 4). For persons with CKD without known hyperkalemia (category 2), consider checking serum potassium level during first cycle of DRSP POPs.*
**Systemic lupus erythematosus** This condition is associated with increased risk for adverse health events as a result of pregnancy (Box 3).								Initiation	Continuation	—		
a. Positive (or unknown) antiphospholipid antibodies	1	1	2*				2*	3	3	2*	4	Persons with SLE are at increased risk for ischemic heart disease, stroke, and VTE. Categories assigned to such conditions in U.S. MEC should be the same for persons with SLE who have these conditions. For all subconditions of SLE, classifications are based on the assumption that no other risk factors for cardiovascular disease are present; these classifications must be modified in the presence of such risk factors.
b. Severe thrombocytopenia	3	2	2				2	3	2	2	2	Persons with SLE are at increased risk for ischemic heart disease, stroke, and VTE. Categories assigned to such conditions in U.S. MEC should be the same for persons with SLE who have these conditions. For all subconditions of SLE, classifications are based on the assumption that no other risk factors for cardiovascular disease are present; these classifications must be modified in the presence of such risk factors. Severe thrombocytopenia increases the risk for bleeding. The category should be assessed according to the severity of thrombocytopenia and its clinical manifestations. In persons with very severe thrombocytopenia who are at risk for spontaneous bleeding, consultation with a specialist and certain pretreatments might be warranted.
c. Immunosuppressive therapy	2	1	2				2	2	2	2	2	Persons with SLE are at increased risk for ischemic heart disease, stroke, and VTE. Categories assigned to such conditions in U.S. MEC should be the same for persons with SLE who have these conditions. For all subconditions of SLE, classifications are based on the assumption that no other risk factors for cardiovascular disease are present; these classifications must be modified in the presence of such risk factors.
d. None of the above	1	1	2				2	2	2	2	2	Persons with SLE are at increased risk for ischemic heart disease, stroke, and VTE. Categories assigned to such conditions in U.S. MEC should be the same for persons with SLE who have these conditions. For all subconditions of SLE, classifications are based on the assumption that no other risk factors for cardiovascular disease are present; these classifications must be modified in the presence of such risk factors.
**High risk for HIV infection**	Initiation	Continuation	Initiation		Continuation		1	1		1	1	**IUD:** Many persons at high risk for HIV infection are also at risk for other STIs (see recommendations for Sexually transmitted infections in U.S. MEC and recommendations on STI screening before IUD placement in U.S. SPR [https://www.cdc.gov/contraception/hcp/usspr]) (*5*).*****
	1*	1*	1*		1*							
**Cirrhosis** Decompensated cirrhosis is associated with increased risk for adverse health events as a result of pregnancy (Box 3).												
a. Compensated (normal liver function)	1		1				1	1		1	1	—
b. Decompensated (impaired liver function)	1		2*				2*	3		2*	4	
**Liver tumors** Hepatocelluar adenoma and malignant liver tumors are associated with increased risk for adverse health events as a result of pregnancy (Box 3).												
a. Benign												
i. Focal nodular hyperplasia	1		2				2	2		2	2	
ii. Hepatoceullular adenoma	1		2*				2*	3		2*	4	
b. Malignant (hepatocellular carcinoma)	1		3				3	3		3	4	
**Sickle cell disease** This condition is associated with increased risk for adverse health events as a result of pregnancy (Box 3).	2		1				1	2/3*		1	4*	**DMPA:** The category should be assessed according to the severity of the condition and risk for thrombosis.*
**Solid organ transplantation** This condition is associated with increased risk for adverse health events as a result of pregnancy (Box 3).	Initiation	Continuation	Initiation			Continuation	—					
a. No graft failure	1*	1*	1*			1*	2	2/3*		2	2	**DMPA:** DMPA use among persons receiving long-term immunosuppressive therapy with a history of, or risk factors for, nontraumatic fractures is classified as category 3. Otherwise, DMPA use for persons with solid organ transplantation is classified as category 2.* **CHC:** Persons with transplant due to Budd-Chiari syndrome should not use CHCs because of the increased risk for thrombosis.*
b. Graft failure	2*	1*	2*			1*	2	2/3*		2	4	**DMPA:** DMPA use among persons receiving long-term immunosuppressive therapy with a history of, or risk factors for, nontraumatic fractures is classified as category 3. Otherwise, DMPA use for persons with solid organ transplantation is classified as category 2.*
**Antiretrovirals used for prevention (PrEP) or treatment of HIV infection*^,†^**												
See the following guidelines for the most up-to-date recommendations on drug-drug interactions between hormonal contraception and antiretrovirals: 1) Recommendations for the Use of Antiretroviral Drugs During Pregnancy and Interventions to Reduce Perinatal HIV Transmission in the United States (https://clinicalinfo.hiv.gov/en/guidelines/perinatal/prepregnancy-counseling-childbearing-age-overview?view=full#table-3) (*6*) and 2) Guidelines for the Use of Antiretroviral Agents in Adults and Adolescents With HIV (https://clinicalinfo.hiv.gov/en/guidelines/hiv-clinical-guidelines-adult-and-adolescent-arv/drug-interactions-overview?view=full) (*7*).												

**Abbreviations:** ARV = antiretroviral; BMI = body mass index; CHC = combined hormonal contraceptive; CKD = chronic kidney disease; Cu-IUD = copper intrauterine device; DMPA = depot medroxyprogesterone acetate; DRSP = drospirenone; DVT = deep venous thrombosis; IUD = intrauterine device; LNG-IUD = levonorgestrel intrauterine device; PE = pulmonary embolism; POC = progestin-only contraceptive; POP = progestin-only pill; PrEP = pre-exposure prophylaxis; SLE = systemic lupus erythematous; STI = sexually transmitted infection; U.S. MEC = *U.S. Medical Eligibility Criteria for Contraceptive Use*; U.S. SPR = *U.S. Selected Practice Recommendations for Contraceptive Use*; VTE = venous thromboembolism.

* Indicates a condition for which the classification changed for one or more contraceptive methods or for which the condition description underwent a substantive modification.

^†^ U.S. MEC recommendations for concurrent use of hormonal contraceptives or IUDs and ARVs for treatment of HIV infection also apply to use of ARVs for PrEP.

**TABLE A2:** Summary of changes for barrier methods from *U.S. Medical Eligibility Criteria for Contraceptive Use, 2016*

Condition	Condom	Spermicide/Vaginal pH modulator*^,†^	Diaphragm/Cap (with spermicide)	Clarification
**Chronic kidney disease*** This condition is associated with increased risk for adverse health events as a result of pregnancy (Box 3).				
a. Current nephrotic syndrome*	1*	1*	1*	—
b. Hemodialysis*	1*	1*	1*	—
c. Peritoneal dialysis*	1*	1*	1*	—
**Cervical cancer (awaiting treatment)**	1	Vaginal pH modulator: 1* Spermicide: 2	1	The cap should not be used. Diaphragm use has no restrictions.
**High risk for HIV infection**	1	Vaginal pH modulator: 1* Spermicide: 4	4	—
**HIV infection** For persons with HIV infection who are not clinically well or not receiving ARV therapy, this condition is associated with increased risk for adverse health events as a result of pregnancy (Box 3).	1	Vaginal pH modulator: 1* Spermicide: 3	3	
**Antiretrovirals used for prevention (PrEP) or treatment of HIV infection*^,§^**	1	1/3/4*******	3/4	No drug interaction between ARV therapy and barrier method use is known. HIV infection is classified as category 1 for vaginal pH modulator and category 3 for spermicide and diaphragm or cap (see recommendations for HIV infection). High risk for HIV infection is classified as category 1 for vaginal pH modulator and category 4 for spermicide and diaphragm or cap (see recommendations for High risk for HIV infection).*

**Abbreviations:** ARV = antiretroviral; PrEP = pre-exposure prophylaxis*.*

* Indicates a condition for which the classification changed for one or more contraceptive methods or for which the condition description underwent a substantive modification.

^†^ The contraceptive method “Spermicide” has been changed to “Spermicide/Vaginal pH modulator.” Recommendations for “Spermicide/Vaginal pH modulator” are the same as those previously for “Spermicide,” with exceptions noted.

^§^ *U.S. Medical Eligibility Criteria for Contraceptive Use* recommendations for concurrent use of barrier methods and ARVs for treatment of HIV infection also apply to use of ARVs for PrEP.

**TABLE A3:** Summary of changes for emergency contraception from *U.S. Medical Eligibility Criteria for Contraceptive Use, 2016*

Condition	Category				Clarification
	Cu-IUD	UPA	LNG	COC	
**Solid organ transplantation** This condition is associated with increased risk for adverse health events as a result of pregnancy (Box 3).					
a. No graft failure	1*	1	1	1	—
b. Graft failure	2*	1	1	1	—

**Abbreviations:** COC = combined oral contraceptive; Cu-IUD = copper intrauterine device; LNG = levonorgestrel; UPA = ulipristal acetate.

* Indicates a condition for which the classification changed for one or more contraceptive methods or for which the condition description underwent a substantive modification.

## Appendix B Classifications for Intrauterine Devices

Classifications for intrauterine devices (IUDs) are for the copper (380 mm^2^) and levonorgestrel (13.5 mg, 19.5 mg, or 52 mg) IUDs (Box B1) (Table B1). IUDs do not protect against sexually transmitted infections (STIs), including HIV infection, and patients using IUDs should be counseled that consistent and correct use of external (male) latex condoms reduces the risk for STIs, including HIV infection (*1*). Use of internal (female) condoms can provide protection from transmission of STIs, although data are limited (*1*). Patients also should be counseled that pre-exposure prophylaxis, when taken as prescribed, is highly effective for preventing HIV infection (*2*).

BOX B1 Categories for classifying intrauterine devices U.S. MEC 1 = A condition for which there is no restriction for the use of the contraceptive method U.S. MEC 2 = A condition for which the advantages of using the method generally outweigh the theoretical or proven risks U.S. MEC 3 = A condition for which the theoretical or proven risks usually outweigh the advantages of using the method U.S. MEC 4 = A condition that represents an unacceptable health risk if the contraceptive method is used **Abbreviation:** U.S. MEC = *U.S. Medical Eligibility Criteria for Contraceptive Use*.

**TABLE B1:** Classifications for intrauterine devices, including the copper intrauterine device and levonorgestrel intrauterine device

Condition	Category				Clarification/Evidence/Comment
	Cu-IUD		LNG-IUD		
**Personal Characteristics and Reproductive History**					
**Pregnancy**	4		4		**Clarification:** The IUD is not indicated during pregnancy and should not be used because of the risk for serious pelvic infection and septic spontaneous abortion.
**Age**					
a. Menarche to <20 years	2		2		**Comment:** Concern exists both about the risk for expulsion from nulliparity and for STIs from sexual behavior in younger age groups (see U.S. SPR for recommendations on STI screening before IUD placement (https://www.cdc.gov/contraception/hcp/usspr) (*3*).
b. ≥20 years	1		1		—
**Parity**					
a. Nulliparous	2		2		**Evidence:** Data conflict about whether IUD use is associated with infertility among nulliparous women, although well-conducted studies suggest no increased risk (*4**–**12*).
b. Parous	1		1		—
**Postpartum** (including cesarean delivery, breastfeeding, or nonbreastfeeding)					
a. <10 minutes after delivery of the placenta	2		2		**Clarification:** Postpartum placement of IUDs is safe and does not appear to increase health risks associated with IUD use such as infection. Higher rates of expulsion during the postpartum period should be considered as they relate to effectiveness, along with patient access to interval placement (i.e., not related to pregnancy) when expulsion rates are lower. **Clarification (breastfeeding):** Breastfeeding provides important health benefits for breastfeeding parent and infant. The U.S. Dietary Guidelines for Americans and American Academy of Pediatrics recommend that infants be exclusively breastfed for about the first 6 months with continued breastfeeding while introducing appropriate complementary foods for 1 year or longer (*13*) or up to age 2 years or longer (*14*). **Evidence:** Studies suggest that immediate postplacental (<10 minutes) and early postpartum (10 minutes up until 72 hours) placement of Cu-IUDs and LNG-IUDs is associated with increased risk for expulsion compared with interval placement (i.e., not related to pregnancy). A meta-analysis found an increased risk for expulsion with immediate postplacental placement (8.6%; range = 0%–31.9%) and early postpartum placement (25.1%; range = 3.5%–46.7%) compared with interval placement (1.6%; range = 0%–4.8%) (Supplementary Appendix, https://stacks.cdc.gov/view/cdc/156516). Although immediate postplacental placement at the time of cesarean delivery might have increased risk for expulsion compared with interval placement, risk appears lower than that for placement at the time of vaginal delivery. Evidence for infection, perforation, and removals for pain or bleeding are limited; however, these events are rare (*15**–**67*). **Evidence (breastfeeding):** Two RCTs found conflicting results on breastfeeding outcomes when LNG-IUDs were initiated immediately postpartum compared with 6–8 weeks postpartum. Initiation of LNG-IUDs immediately postpartum had no other harmful effect on infant health, growth, or development (*19**,**68*). Breastfeeding women using IUDs do not have an increased risk for certain IUD-related adverse events including expulsion, infection, pain, or bleeding compared with nonbreastfeeding women. The risk for perforation is increased independently among breastfeeding women and among women ≤36 weeks postpartum, compared with nonpostpartum women; however, the absolute risk for perforation remains low (*15**–**67**,**69*). **Comment:** Risk factors for breastfeeding difficulties include previous breastfeeding difficulties, certain medical conditions, certain perinatal complications, and preterm birth. For all breastfeeding persons, with or without risk factors for breastfeeding difficulties, discussions about contraception should include information about risks, benefits, and alternatives.
b. 10 minutes after delivery of the placenta to <4 weeks	2		2		**Clarification:** Postpartum placement of IUDs is safe and does not appear to increase health risks associated with IUD use such as infection. Higher rates of expulsion during the postpartum period should be considered as they relate to effectiveness, along with patient access to interval placement (i.e., not related to pregnancy) when expulsion rates are lower. **Clarification (breastfeeding):** Breastfeeding provides important health benefits for breastfeeding parent and infant. The U.S. Dietary Guidelines for Americans and American Academy of Pediatrics recommend that infants be exclusively breastfed for about the first 6 months with continued breastfeeding while introducing appropriate complementary foods for 1 year or longer (*13*) or up to age 2 years or longer (*14*). **Evidence:** Studies suggest that immediate postplacental (<10 minutes) and early postpartum (10 minutes up until 72 hours) placement of Cu-IUDs and LNG-IUDs is associated with increased risk for expulsion compared with interval placement (i.e., not related to pregnancy). A meta-analysis found an increased risk for expulsion with immediate postplacental placement (8.6%; range = 0%–31.9%) and early postpartum placement (25.1%; range = 3.5%–46.7%) compared with interval placement (1.6%; range = 0%–4.8%) (Supplementary Appendix, https://stacks.cdc.gov/view/cdc/156516). Although immediate postplacental placement at the time of cesarean delivery might have increased risk for expulsion compared with interval placement, risk appears lower than that for placement at the time of vaginal delivery. Evidence for infection, perforation, and removals for pain or bleeding are limited; however, these events are rare (*15**–**67*). **Evidence (breastfeeding):** Two RCTs found conflicting results on breastfeeding outcomes when LNG-IUDs were initiated immediately postpartum compared with 6–8 weeks postpartum. Initiation of LNG-IUDs immediately postpartum had no other harmful effect on infant health, growth, or development (*19**,**68*). Breastfeeding women using IUDs do not have an increased risk for certain IUD-related adverse events including expulsion, infection, pain, or bleeding compared with nonbreastfeeding women. The risk for perforation is increased independently among breastfeeding women and among women ≤36 weeks postpartum, compared with nonpostpartum women; however, the absolute risk for perforation remains low (*15**–**67**,**69*). **Comment:** Risk factors for breastfeeding difficulties include previous breastfeeding difficulties, certain medical conditions, certain perinatal complications, and preterm birth. For all breastfeeding persons, with or without risk factors for breastfeeding difficulties, discussions about contraception should include information about risks, benefits, and alternatives.
c. ≥4 weeks	1		1		**Clarification:** Postpartum placement of IUDs is safe and does not appear to increase health risks associated with IUD use such as infection. Higher rates of expulsion during the postpartum period should be considered as they relate to effectiveness, along with patient access to interval placement (i.e., not related to pregnancy) when expulsion rates are lower. **Clarification (breastfeeding):** Breastfeeding provides important health benefits for breastfeeding parent and infant. The U.S. Dietary Guidelines for Americans and American Academy of Pediatrics recommend that infants be exclusively breastfed for about the first 6 months with continued breastfeeding while introducing appropriate complementary foods for 1 year or longer (*13*) or up to age 2 years or longer (*14*). **Evidence (breastfeeding):** Initiation of LNG-IUDs at 4 weeks postpartum or later demonstrated no detrimental effect on breastfeeding outcomes and no harmful effect on infant health, growth, or development (*19**,**68*). Breastfeeding women using IUDs do not have an increased risk for certain IUD-related adverse events including expulsion, infection, pain, or bleeding compared with nonbreastfeeding women. The risk for perforation is increased independently among breastfeeding women and among women ≤36 weeks postpartum, compared with nonpostpartum women; however, the absolute risk for perforation remains low (*15**–**67**,**69*). **Comment:** Risk factors for breastfeeding difficulties include previous breastfeeding difficulties, certain medical conditions, certain perinatal complications, and preterm birth. For all breastfeeding persons, with or without risk factors for breastfeeding difficulties, discussions about contraception should include information about risks, benefits, and alternatives.
d. Postpartum sepsis	4		4		**Comment:** Theoretical concern exists that postpartum placement of an IUD in a person with recent chorioamnionitis or current endometritis might be associated with increased complications.
**Postabortion **(spontaneous or induced)					
a. First trimester abortion					**Clarification:** IUDs may be placed immediately after abortion completion. **Evidence:** Risk for complications from immediate versus delayed placement of an IUD after abortion did not differ. Expulsion was greater when an IUD was placed after a second trimester procedural abortion than when placed after a first trimester procedural abortion. Safety or expulsion for postabortion placement of an LNG-IUD did not differ from that of a Cu-IUD (*70*).
i. Procedural (surgical)	1		1		
ii. Medication	1		1		
iii. Spontaneous abortion with no intervention	1		1		
b. Second trimester abortion					
i. Procedural (surgical)	2		2		
ii. Medication	2		2		
iii. Spontaneous abortion with no intervention	2		2		
c. Immediate postseptic abortion	4		4		**Comment:** Placement of an IUD might substantially worsen the condition.
**Past ectopic pregnancy**	1		1		**Comment:** The absolute risk for ectopic pregnancy is extremely low because of the high effectiveness of IUDs. However, when a person becomes pregnant during IUD use, the relative likelihood of ectopic pregnancy increases substantially.
**History of pelvic surgery** (see recommendations for Postpartum [including cesarean delivery])	1		1		—
**Smoking**					
a. Age <35 years	1		1		—
b. Age ≥35 years					
i. <15 cigarettes per day	1		1		—
ii. ≥15 cigarettes per day	1		1		—
**Obesity**					
a. BMI ≥30 kg/m^2^	1		1		—
b. Menarche to <18 years and BMI ≥30 kg/m^2^	1		1		—
**History of bariatric surgery** This condition is associated with increased risk for adverse health events as a result of pregnancy (Box 3).					
a. Restrictive procedures: decrease storage capacity of the stomach (vertical banded gastroplasty, laparoscopic adjustable gastric band, or laparoscopic sleeve gastrectomy)	1		1		—
b. Malabsorptive procedures: decrease absorption of nutrients and calories by shortening the functional length of the small intestine (Roux-en-Y gastric bypass or biliopancreatic diversion)	1		1		—
**Surgery**					
a. Minor surgery without immobilization	1		1		—
b. Major surgery					
i. Without prolonged immobilization	1		1		—
ii. With prolonged immobilization	1		1		**Evidence:** No direct evidence was identified on risk for thrombosis with POC use among those undergoing major surgery (Supplementary Appendix, https://stacks.cdc.gov/view/cdc/156516).
**Cardiovascular Disease**					
**Multiple risk factors for atherosclerotic cardiovascular disease** (e.g., older age, smoking, diabetes, hypertension, low HDL, high LDL, or high triglyceride levels)	1		2		—
**Hypertension** Systolic blood pressure ≥160 mm Hg or diastolic blood pressure ≥100 mm Hg are associated with increased risk for adverse health events as a result of pregnancy (Box 3).					
a. Adequately controlled hypertension	1		1		**Clarification:** For all categories of hypertension, classifications are based on the assumption that no other risk factors for cardiovascular disease exist. When multiple risk factors do exist, risk for cardiovascular disease might increase substantially. A single reading of blood pressure level is not sufficient to classify a person as hypertensive.
b. Elevated blood pressure levels (properly taken measurements)					**Clarification:** For all categories of hypertension, classifications are based on the assumption that no other risk factors for cardiovascular disease exist. When multiple risk factors do exist, risk for cardiovascular disease might increase substantially. A single reading of blood pressure level is not sufficient to classify a person as hypertensive. **Comment:** Theoretical concern exists about the effect of LNG on lipids. Use of Cu-IUDs has no restrictions.
i. Systolic 140–159 mm Hg or diastolic 90–99 mm Hg	1		1		
ii. Systolic ≥160 mm Hg or diastolic ≥100 mm Hg	1		2		
c. Vascular disease	1		2		**Clarification:** For all categories of hypertension, classifications are based on the assumption that no other risk factors for cardiovascular disease exist. When multiple risk factors do exist, risk for cardiovascular disease might increase substantially. A single reading of blood pressure level is not sufficient to classify a person as hypertensive. **Comment:** Theoretical concern exists about the effect of LNG on lipids. Use of Cu-IUDs has no restrictions.
**History of high blood pressure during pregnancy** (when current blood pressure is measurable and normal)	1		1		—
**Deep venous thrombosis/ Pulmonary embolism** This condition is associated with increased risk for adverse health events as a result of pregnancy (Box 3).					
a. Current or history of DVT/PE, receiving anticoagulant therapy (therapeutic dose) (e.g., acute DVT/PE or long-term therapeutic dose)	2		2		**Clarification (Cu-IUD):** Persons using anticoagulant therapy are at risk for gynecologic complications of therapy, such as heavy or prolonged bleeding. Cu-IUDs might worsen bleeding. **Clarification (LNG-IUD):** Persons using anticoagulant therapy are at risk for gynecologic complications of therapy, such as heavy or prolonged bleeding. LNG-IUDs can be of benefit in preventing or treating this complication. When a contraceptive method is used as a therapy, rather than solely to prevent pregnancy, the risk/benefit ratio might differ and should be considered on a case-by-case basis. **Evidence:** Limited evidence was identified on use of POCs or Cu-IUDs among women with acute DVT/PE receiving anticoagulant therapy (Supplementary Appendix, https://stacks.cdc.gov/view/cdc/156516). In one study among women with a history of acute VTE currently receiving therapeutic anticoagulant therapy (i.e., rivaroxaban or enoxaparin/vitamin K antagonist [warfarin or acenocoumarol]), the incidence of recurrent VTE was similar among estrogen users (CHC or estrogen-only pills), POC users, and women not on hormonal therapy (*71*). Limited evidence suggests that placement of a Cu-IUD or LNG-IUD does not increase risk for bleeding complications in women receiving anticoagulant therapy (Supplementary Appendix, https://stacks.cdc.gov/view/cdc/156516).
b. History of DVT/PE, receiving anticoagulant therapy (prophylactic dose)					**Clarification (Cu-IUD):** Persons using anticoagulant therapy are at risk for gynecologic complications of therapy, such as heavy or prolonged bleeding. Cu-IUDs might worsen bleeding. **Clarification (LNG-IUD):** Persons using anticoagulant therapy are at risk for gynecologic complications of therapy, such as heavy or prolonged bleeding. LNG-IUDs can be of benefit in preventing or treating this complication. When a contraceptive method is used as a therapy, rather than solely to prevent pregnancy, the risk/benefit ratio might differ and should be considered on a case-by-case basis. **Evidence:** Limited evidence suggests that placement of the LNG-IUD does not increase risk for bleeding complications in women receiving anticoagulant therapy (Supplementary Appendix, https://stacks.cdc.gov/view/cdc/156516).
i. Higher risk for recurrent DVT/PE (one or more risk factors)	2		2		
• Thrombophilia (e.g., factor V Leiden mutation; prothrombin gene mutation; protein S, protein C, and antithrombin deficiencies; or antiphospholipid syndrome) • Active cancer (metastatic, receiving therapy, or within 6 months after clinical remission), excluding nonmelanoma skin cancer • History of recurrent DVT/PE					
ii. Lower risk for recurrent DVT/PE (no risk factors)	2		2		
c. History of DVT/PE, not receiving anticoagulant therapy					
i. Higher risk for recurrent DVT/PE (one or more risk factors)	1		2		—
• History of estrogen-associated DVT/PE • Pregnancy-associated DVT/PE • Idiopathic DVT/PE • Thrombophilia (e.g., factor V Leiden mutation; prothrombin gene mutation; protein S, protein C, and antithrombin deficiencies; or antiphospholipid syndrome) • Active cancer (metastatic, receiving therapy, or within 6 months after clinical remission), excluding nonmelanoma skin cancer • History of recurrent DVT/PE					
ii. Lower risk for recurrent DVT/PE (no risk factors)	1		2		—
d. Family history (first-degree relatives)	1		1		—
**Thrombophilia** (e.g., factor V Leiden mutation; prothrombin gene mutation; protein S, protein C, and antithrombin deficiencies; or antiphospholipid syndrome) This condition is associated with increased risk for adverse health events as a result of pregnancy (Box 3).	1		2		**Clarification:** Routine screening in the general population before contraceptive initiation is not recommended. **Clarification:** If a person has current or history of DVT/PE, see recommendations for DVT/PE. **Clarification**: Classification of antiphospholipid syndrome includes presence of a clinical feature (e.g., thrombosis or obstetric morbidity) and persistently abnormal antiphospholipid antibody test on two or more occasions at least 12 weeks apart (*72*). **Evidence:** Limited evidence was identified on LNG-IUD use among persons with thrombophilia. Among women with factor V Leiden mutation, one study found that women using LNG-IUD had similar risk for venous thrombosis as those not using hormonal contraception (*73*). No evidence was identified on POC use among persons with prothrombin gene mutation, protein S deficiency, protein C deficiency, antithrombin deficiency, or antiphospholipid syndrome (Supplementary Appendix, https://stacks.cdc.gov/view/cdc/156516).
**Superficial venous disorders**					
a. Varicose veins	1		1		—
b. Superficial venous thrombosis (acute or history)	1		1		—
**Current and history of ischemic heart disease** This condition is associated with increased risk for adverse health events as a result of pregnancy (Box 3).	1		Initiation	Continuation	**Comment:** Theoretical concern exists about the effect of LNG on lipids. Use of Cu-IUDs has no restrictions.
			2	3	
**Stroke** (history of cerebrovascular accident) This condition is associated with increased risk for adverse health events as a result of pregnancy (Box 3).	1		2		**Comment:** Theoretical concern exists about the effect of LNG on lipids. Use of Cu-IUDs has no restrictions.
**Valvular heart disease** Complicated valvular heart disease is associated with increased risk for adverse health events as a result of pregnancy (Box 3).					**Comment:** According to the American Heart Association, administration of prophylactic antibiotics solely to prevent endocarditis is not recommended for patients who undergo genitourinary tract procedures, including placement or removal of IUDs (*74*).
a. Uncomplicated	1		1		
b. Complicated (pulmonary hypertension, risk for atrial fibrillation, or history of subacute bacterial endocarditis)	1		1		
**Peripartum cardiomyopathy** This condition is associated with increased risk for adverse health events as a result of pregnancy (Box 3).					**Evidence:** No direct evidence exists on the safety of IUDs among women with peripartum cardiomyopathy. Limited indirect evidence from noncomparative studies did not demonstrate any cases of arrhythmia or infective endocarditis in women with cardiac disease who used IUDs (*75*). **Comment:** IUD placement might induce cardiac arrhythmias in healthy persons; persons with peripartum cardiomyopathy have a high incidence of cardiac arrhythmias.
a. Normal or mildly impaired cardiac function (New York Heart Association Functional Class I or II: no limitation of activities or slight, mild limitation of activity) (*76*)					
i. <6 months	2		2		
ii. ≥6 months	2		2		
b. Moderately or severely impaired cardiac function (New York Heart Association Functional Class III or IV: marked limitation of activity or should be at complete rest) (*76*)	2		2		
**Renal Disease**					
**Chronic kidney disease** This condition is associated with increased risk for adverse health events as a result of pregnancy (Box 3).	Initiation	Continuation	Initiation	Continuation	
a. Current nephrotic syndrome	1	1	2	2	**Comment:** A person might have CKD without current nephrotic syndrome, but might have other conditions often associated with CKD (e.g., diabetes, hypertension, SLE). See recommendations for other conditions if they apply.
b. Hemodialysis	1	1	2	2	**Evidence:** No comparative studies were identified on the safety of IUD use among persons with CKD on hemodialysis (Supplementary Appendix, https://stacks.cdc.gov/view/cdc/156516). One case report of LNG-IUD use in a person with CKD on hemodialysis reported improved abnormal uterine bleeding and anemia (*77*). **Comment:** A person might have CKD without hemodialysis, but might have other conditions often associated with CKD (e.g., diabetes, hypertension, and SLE). See recommendations for other conditions if they apply.
c. Peritoneal dialysis	2	1	2	2	**Evidence:** No comparative studies were identified on IUD use among persons with CKD on peritoneal dialysis (Supplementary Appendix, https://stacks.cdc.gov/view/cdc/156516). Four case reports of IUD use among women with CKD on peritoneal dialysis identified one case of peritoneal allergic reaction (78), three cases of peritonitis (*78**–**80*) and one case of TOA (*78*). **Comment:** A person might have CKD without peritoneal dialysis, but might have other conditions often associated with CKD (e.g., diabetes, hypertension, and SLE). See recommendations for other conditions if they apply.
**Rheumatic Diseases**					
**Systemic lupus erythematosus** This condition is associated with increased risk for adverse health events as a result of pregnancy (Box 3).	Initiation	Continuation	—		
a. Positive (or unknown) antiphospholipid antibodies	1	1	2		**Clarification:** Persons with SLE are at increased risk for ischemic heart disease, stroke, and VTE. Categories assigned to such conditions in U.S. MEC should be the same for persons with SLE who have these conditions. For all subconditions of SLE, classifications are based on the assumption that no other risk factors for cardiovascular disease are present; these classifications must be modified in the presence of such risk factors (*81**–**99*). **Evidence:** No direct evidence was identified on POC use among persons with SLE with antiphospholipid antibodies (*100*) (Supplementary Appendix, https://stacks.cdc.gov/view/cdc/156516).
b. Severe thrombocytopenia	3	2	2		**Clarification:** Persons with SLE are at increased risk for ischemic heart disease, stroke, and VTE. Categories assigned to such conditions in U.S. MEC should be the same for persons with SLE who have these conditions. For all subconditions of SLE, classifications are based on the assumption that no other risk factors for cardiovascular disease are present; these classifications must be modified in the presence of such risk factors (*81**–**99*). **Clarification:** Severe thrombocytopenia increases the risk for bleeding. The category should be assessed according to the severity of thrombocytopenia and its clinical manifestations. In persons with very severe thrombocytopenia who are at risk for spontaneous bleeding, consultation with a specialist and certain pretreatments might be warranted. **Evidence:** The LNG-IUD might be a useful treatment for menorrhagia in women with severe thrombocytopenia (*94*).
c. Immunosuppressive therapy	2	1	2		**Clarification:** Persons with SLE are at increased risk for ischemic heart disease, stroke, and VTE. Categories assigned to such conditions in U.S. MEC should be the same for persons with SLE who have these conditions. For all subconditions of SLE, classifications are based on the assumption that no other risk factors for cardiovascular disease are present; these classifications must be modified in the presence of such risk factors (*81**–**99*).
d. None of the above	1	1	2		**Clarification:** Persons with SLE are at increased risk for ischemic heart disease, stroke, and VTE. Categories assigned to such conditions in U.S. MEC should be the same for persons with SLE who have these conditions. For all subconditions of SLE, classifications are based on the assumption that no other risk factors for cardiovascular disease are present; these classifications must be modified in the presence of such risk factors (*81**–**99*).
**Rheumatoid arthritis**	Initiation	Continuation	Initiation	Continuation	—
a. Not receiving immunosuppressive therapy	1	1	1	1	—
b. Receiving immunosuppressive therapy	2	1	2	1	—
**Neurologic Conditions**					
**Headaches**					
a. Nonmigraine (mild or severe)	1		1		—
b. Migraine					
i. Without aura (includes menstrual migraine)	1		1		**Evidence:** No studies directly examined the risk for stroke among women with migraine using LNG-IUDs (*101*). Limited evidence demonstrated that women using LNG-IUDs do not have an increased risk for ischemic stroke compared with women not using hormonal contraceptives (*102*). **Comment:** Menstrual migraine is a subtype of migraine without aura. For more information see the International Headache Society’s *International Classification of Headache Disorders, 3rd ed.* (https://ichd-3.org) (*103*).
ii. With aura	1		1		
**Epilepsy** This condition is associated with increased risk for adverse health events as a result of pregnancy (Box 3).	1		1		—
**Multiple sclerosis**					
a. Without prolonged immobility	1		1		—
b. With prolonged immobility	1		1		—
**Depressive Disorders**					
**Depressive disorders**	1		1		**Clarification:** If a person is receiving psychotropic medications or St. John’s wort, see recommendations for Drug Interactions. **Evidence:** The frequency of psychiatric hospitalizations for women with bipolar disorder or depression did not significantly differ among women using DMPA, LNG-IUD, Cu-IUD, or sterilization (*104*).
**Reproductive Tract Infections and Disorders**					
**Vaginal bleeding patterns**			Initiation	Continuation	—
a. Irregular pattern without heavy bleeding	1		1	1	
b. Heavy or prolonged bleeding (includes regular and irregular patterns)	2		1	2	**Clarification:** Unusually heavy bleeding should raise suspicion of a serious underlying condition. **Evidence:** Evidence from studies examining the treatment effects of the LNG-IUD among women with heavy or prolonged bleeding reported no increase in adverse effects and found the LNG-IUD to be beneficial in treating menorrhagia (*105**–**112*).
**Unexplained vaginal bleeding** (suspicious for serious condition) before evaluation	Initiation	Continuation	Initiation	Continuation	**Clarification:** If pregnancy or an underlying pathological condition (e.g., pelvic malignancy) is suspected, it must be evaluated and the category adjusted after evaluation. The IUD does not need to be removed before evaluation.
	4	2	4	2	
**Endometriosis**	2		1		**Evidence:** LNG-IUD use among women with endometriosis decreased dysmenorrhea, pelvic pain, and dyspareunia (*113**–**117*).
**Benign ovarian tumors** (including cysts)	1		1		—
**Severe dysmenorrhea**	2		1		**Comment:** Dysmenorrhea might intensify with Cu-IUD use. LNG-IUD use has been associated with reduction of dysmenorrhea.
**Gestational trophoblastic disease** This condition is associated with increased risk for adverse health events as a result of pregnancy (Box 3).					
a. Suspected gestational trophoblastic disease (immediate postevacuation)					**Clarification:** For all subconditions of gestational trophoblastic disease, classifications are based on the assumption that persons are under close medical supervision because of the need for monitoring of β-hCG levels for appropriate disease surveillance. **Evidence:** Limited evidence suggests that women using an IUD after uterine evacuation for a molar pregnancy are not at greater risk for postmolar trophoblastic disease than are women using other methods of contraception (*118*). **Comment:** The risk for expulsion immediately postevacuation for gestational trophoblastic disease is unknown. Expulsion is greater after IUD placement immediately postevacuation for a spontaneous or induced abortion in the second trimester compared with IUD placement after a first trimester abortion.
i. Uterine size first trimester	1		1		
ii. Uterine size second trimester	2		2		
b. Confirmed gestational trophoblastic disease (after initial evacuation and during monitoring)	Initiation	Continuation	Initiation	Continuation	—
i. Undetectable or nonpregnant β-hCG levels	1	1	1	1	**Clarification:** For all subconditions of gestational trophoblastic disease, classifications are based on the assumption that persons are under close medical supervision because of the need for monitoring of β-hCG levels for appropriate disease surveillance. **Evidence:** Limited evidence suggests that women using an IUD after uterine evacuation for a molar pregnancy are not at greater risk for postmolar trophoblastic disease than are women using other methods of contraception (*118*). **Comment:** Once β-hCG levels have decreased to nonpregnant levels, the risk for disease progression is likely to be very low.
ii. Decreasing β-hCG levels	2	1	2	1	**Clarification:** For all subconditions of gestational trophoblastic disease, classifications are based on the assumption that persons are under close medical supervision because of the need for monitoring of β-hCG levels for appropriate disease surveillance. **Clarification:** For persons at higher risk for disease progression, the benefits of effective contraception must be weighed against the potential need for early IUD removal. **Evidence:** Limited evidence suggests that women using an IUD after uterine evacuation for a molar pregnancy are not at greater risk for postmolar trophoblastic disease than are women using other methods of contraception (*118*).
iii. Persistently elevated β-hCG levels or malignant disease, with no evidence or suspicion of intrauterine disease	2	1	2	1	**Clarification:** For all subconditions of gestational trophoblastic disease, classifications are based on the assumption that persons are under close medical supervision because of the need for monitoring of β-hCG levels for appropriate disease surveillance. **Evidence:** Limited evidence suggests that women using an IUD after uterine evacuation for a molar pregnancy are not at greater risk for postmolar trophoblastic disease than are women using other methods of contraception (*118*).
iv. Persistently elevated β-hCG levels or malignant disease, with evidence or suspicion of intrauterine disease	4	2	4	2	**Clarification:** For all subconditions of gestational trophoblastic disease, classifications are based on the assumption that persons are under close medical supervision because of the need for monitoring of β-hCG levels for appropriate disease surveillance. **Evidence:** Limited evidence suggests that women using an IUD after uterine evacuation for a molar pregnancy are not at greater risk for postmolar trophoblastic disease than are women using other methods of contraception (*118*). **Comment:** For persons with suspected or confirmed intrauterine disease, an IUD should not be placed because of theoretical risk for perforation, infection, and hemorrhage. For persons who already have an IUD in place, individual circumstance along with the benefits of effective contraception must be weighed against theoretical risks of either removal or continuation of the IUD.
**Cervical ectropion**	1		1		—
**Cervical intraepithelial neoplasia**	1		2		**Comment:** Theoretical concern exists that LNG-IUDs might enhance progression of cervical intraepithelial neoplasia.
**Cervical cancer** (awaiting treatment)	Initiation	Continuation	Initiation	Continuation	**Comment:** Concern exists about the increased risk for infection and bleeding at placement. The IUD most likely will need to be removed at the time of treatment but until then, the person is at risk for pregnancy.
	4	2	4	2	
**Breast disease** Breast cancer is associated with increased risk for adverse health events as a result of pregnancy (Box 3).					
a. Undiagnosed mass	1		2		**Clarification (LNG-IUD):** Evaluation of mass should be pursued as early as possible.
b. Benign breast disease	1		1		—
c. Family history of cancer	1		1		—
d. Breast cancer					**Comment:** Breast cancer is a hormonally sensitive tumor. Concerns about progression of the disease might be less with LNG-IUDs than with COCs or higher-dose POCs.
i. Current	1		4		
ii. Past and no evidence of current disease for 5 years	1		3		
**Endometrial hyperplasia**	1		1		**Evidence:** Among women with endometrial hyperplasia, no adverse health events occurred with LNG-IUD use; most women experienced disease regression (*119*).
**Endometrial cancer** This condition is associated with increased risk for adverse health events as a result of pregnancy (Box 3).	Initiation	Continuation	Initiation	Continuation	**Comment:** Concern exists about the increased risk for infection, perforation, and bleeding at placement. The IUD most likely will need to be removed at the time of treatment, but until then, the person is at risk for pregnancy.
	4	2	4	2	
**Ovarian cancer** This condition is associated with increased risk for adverse health events as a result of pregnancy (Box 3).	1		1		**Comment:** Persons with ovarian cancer who undergo fertility-sparing treatment and need contraception can use an IUD.
**Uterine fibroids**	2		2		**Evidence:** Among women with uterine fibroids using an LNG-IUD, most experienced improvements in serum levels of hemoglobin, hematocrit, and ferritin and in menstrual blood loss (*120*). Rates of LNG-IUD expulsion were higher in women with uterine fibroids (11%) than in women without fibroids (0%–3%); these findings were either not statistically significant or significance testing was not conducted (*120*). Rates of expulsion found in noncomparative studies ranged from 0%–20% (*120*). **Comment:** Persons with heavy or prolonged bleeding should be assigned the category for that condition.
**Anatomical abnormalities**					
a. Distorted uterine cavity (any congenital or acquired uterine abnormality distorting the uterine cavity in a manner that is incompatible with IUD placement)	4		4		**Comment:** An anatomical abnormality that distorts the uterine cavity might preclude proper IUD placement.
b. Other abnormalities (including cervical stenosis or cervical lacerations) not distorting the uterine cavity or interfering with IUD placement	2		2		—
**Pelvic inflammatory disease**	Initiation	Continuation	Initiation	Continuation	**Clarification (continuation):** Treat the PID using appropriate antibiotics. The IUD usually does not need to be removed if the person wants to continue using it. Continued use of an IUD depends on the person’s informed choice and current risk factors for STIs and PID. **Evidence:** Among IUD users treated for PID, clinical course did not differ regardless of whether the IUD was removed or left in place (*121*).
a. Current PID	4	2	4	2	
b. Past PID					**Comment:** IUDs do not protect against STIs, including HIV infection, or PID. In persons at low risk for STIs, IUD placement poses little risk for PID.
i. With subsequent pregnancy	1	1	1	1	
ii. Without subsequent pregnancy	2	2	2	2	
**Sexually transmitted infections**	Initiation	Continuation	Initiation	Continuation	
a. Current purulent cervicitis or chlamydial infection or gonococcal infection	4	2	4	2	**Clarification (continuation):** Treat the STI using appropriate antibiotics. The IUD usually does not need to be removed if the person wants to continue using it. Continued use of an IUD depends on the person’s informed choice and current risk factors for STIs and PID. **Evidence:** Among women who had an IUD placed, the absolute risk for subsequent PID was low among women with STI at the time of placement but greater than among women with no STI at the time of IUD placement (*122**–**128*).
b. Vaginitis (including *Trichomonas vaginalis* and bacterial vaginosis)	2	2	2	2	—
c. Other factors related to STIs	2	2	2	2	**Clarification (initiation):** Most persons do not require additional STI screening at the time of IUD placement. If a person with risk factors for STIs has not been screened for gonorrhea and chlamydia according to CDC STI treatment guidelines (*1*), screening may be performed at the time of IUD placement and placement should not be delayed. **Evidence:** Women who undergo same-day STI screening and IUD placement have low incidence rates of PID. Algorithms for predicting PID among women with risk factors for STIs have poor predictive value. Risk for PID among women with risk factors for STIs is low (*129*).
**HIV**					
**High risk for HIV infection**	Initiation	Continuation	Initiation	Continuation	**Clarification:** Many persons at high risk for HIV infection are also at risk for other STIs (see recommendations for Sexually transmitted Infections in U.S. MEC and recommendations on STI screening before IUD placement in U.S. SPR (https://www.cdc.gov/contraception/hcp/usspr) (*3*). **Evidence:** High-quality evidence from one RCT, along with low-quality evidence from two observational studies, suggested no increased risk for HIV acquisition with Cu-IUD use. No studies were identified for LNG-IUDs (*130**–**132*).
	1	1	1	1	
**HIV infection** For persons with HIV infection who are not clinically well or not receiving ARV therapy, this condition is associated with increased risk for adverse health events as a result of pregnancy (Box 3).					**Evidence:** Among IUD users, limited evidence demonstrates a low risk for PID among HIV-infected women using IUDs and no higher risk for pelvic infectious complications in HIV-infected than in HIV-noninfected women or among women with varying degrees of HIV severity. IUD use did not adversely affect progression of HIV infection during 6–45 months of follow-up or when compared with hormonal contraceptive use among HIV-infected women. Furthermore, IUD use among HIV-infected women was not associated with increased risk for transmission to sex partners or with increased genital viral shedding (*133*).
a. Clinically well receiving ARV therapy	1	1	1	1	
b. Not clinically well or not receiving ARV therapy	2	1	2	1	
**Other Infections**					
**Schistosomiasis** Schistosomiasis with fibrosis of the liver is associated with increased risk for adverse health events as a result of pregnancy (Box 3).					
A. Uncomplicated	1		1		—
b. Fibrosis of the liver (if severe, see recommendations for Cirrhosis)	1		1		—
**Tuberculosis** This condition is associated with increased risk for adverse health events as a result of pregnancy (Box 3).	Initiation	Continuation	Initiation	Continuation	
a. Nonpelvic	1	1	1	1	—
b. Pelvic	4	3	4	3	**Comment:** Placement of an IUD might substantially worsen the condition.
**Malaria**	1		1		—
**Endocrine Conditions**					
**Diabetes** Insulin-dependent diabetes diabetes with nephropathy, retinopathy, or neuropathy; diabetes with other vascular disease; or diabetes of >20 years’ duration are associated with increased risk for adverse health events as a result of pregnancy (Box 3).					
a. History of gestational disease	1		1		—
b. Nonvascular disease					**Evidence:** Limited evidence on the use of the LNG-IUD among women with insulin-dependent or non–insulin-dependent diabetes suggests that these methods have little effect on short-term or long-term diabetes control (e.g., glycosylated hemoglobin levels), hemostatic markers, or lipid profile (*134**,**135*).
i. Non-insulin dependent	1		2		
ii. Insulin dependent	1		2		
c. Nephropathy, retinopathy, or neuropathy	1		2		—
d. Other vascular disease or diabetes of >20 years’ duration	1		2		—
**Thyroid disorders**					
a. Simple goiter	1		1		—
b. Hyperthyroid	1		1		—
c. Hypothyroid	1		1		—
**Gastrointestinal Conditions**					
**Inflammatory bowel disease** (ulcerative colitis or Crohn’s disease)	1		1		**Evidence:** Although two case reports described three women with IBD who experienced exacerbation of disease 5 days–25 months after LNG-IUD placement (*136*), no comparative studies have examined the safety of IUD use among women with IBD (*136*).
**Gallbladder disease**					
a. Asymptomatic	1		2		—
b. Symptomatic					
i. Current	1		2		—
ii. Treated by cholecystectomy	1		2		—
iii. Medically treated	1		2		—
**History of cholestasis**					
a. Pregnancy related	1		1		—
b. Past COC related	1		2		**Comment:** Concern exists that history of COC related cholestasis might predict subsequent cholestasis with LNG use. Whether risk exists with use of LNG-IUD is unclear.
**Viral hepatitis**					
a. Acute or flare	1		1		**Evidence:** No direct evidence was identified on IUD use among persons with viral hepatitis (Supplementary Appendix, https://stacks.cdc.gov/view/cdc/156516).
b. Chronic	1		1		**Evidence:** No direct evidence was identified on IUD use among persons with viral hepatitis (Supplementary Appendix, https://stacks.cdc.gov/view/cdc/156516).
**Cirrhosis** Decompensated cirrhosis is associated with increased risk for adverse health events as a result of pregnancy (Box 3).					
a. Compensated (normal liver function)	1		1		**Evidence:** No direct evidence was identified on IUD use among persons with cirrhosis (Supplementary Appendix, https://stacks.cdc.gov/view/cdc/156516).
b. Decompensated (impaired liver function)	1		2		**Evidence:** No direct evidence was identified on IUD use among persons with cirrhosis (Supplementary Appendix, https://stacks.cdc.gov/view/cdc/156516). **Comment:** Hepatic metabolism of exogenous hormones might be impaired in persons with liver dysfunction, which could lead to increased progestin levels in circulation and progestin-related side effects and adverse events, which might vary by dose and formulation. Any progestin-related hepatotoxicity might be less tolerated in persons with existing liver dysfunction.
**Liver tumors** Hepatocellular adenoma and malignant liver tumors are associated with increased risk for adverse health events as a result of pregnancy (Box 3).					
a. Benign					
i. Focal nodular hyperplasia	1		2		**Evidence:** Limited evidence suggests that progestin use does not influence either progression or regression of focal nodular hyperplasia (Supplementary Appendix, https://stacks.cdc.gov/view/cdc/156516).
ii. Hepatocellular adenoma	1		2		**Evidence:** Limited evidence suggests that hepatocellular adenomas generally regress or remain stable during progestin use (Supplementary Appendix, https://stacks.cdc.gov/view/cdc/156516).
b. Malignant (hepatocellular carcinoma)	1		3		**Evidence:** No direct evidence was identified on IUD use among persons with hepatocellular carcinoma (Supplementary Appendix, https://stacks.cdc.gov/view/cdc/156516).
**Respiratory Conditions**					
**Cystic fibrosis** This condition is associated with increased risk for adverse health events as a result of pregnancy (Box 3).	1		1		**Clarification:** Persons with cystic fibrosis are at increased risk for diabetes, liver disease, gallbladder disease, and VTE (particularly related to use of central venous catheters) and are frequently prescribed antibiotics. Categories assigned to such conditions in U.S. MEC should be the same for persons with cystic fibrosis who have these conditions. For cystic fibrosis, classifications are based on the assumption that no other conditions are present; these classifications must be modified in the presence of such conditions.
**Hematologic Conditions**					
**Thalassemia**	2		1		**Comment:** Concern exists about an increased risk for blood loss with Cu-IUDs.
**Sickle cell disease** This condition is associated with increased risk for adverse health events as a result of pregnancy (Box 3).	2		1		**Comment:** Concern exists about an increased risk for blood loss with Cu-IUDs.
**Iron deficiency anemia**	2		1		**Comment:** Concern exists about an increased risk for blood loss with Cu-IUDs.
**Solid Organ Transplantation**					
**Solid organ transplantation** This condition is associated with increased risk for adverse health events as a result of pregnancy (Box 3).	Initiation	Continuation	Initiation	Continuation	**Evidence:** Limited evidence suggests that LNG-IUD use among solid organ transplantation recipients does not increase risk for pelvic infections or decrease contraceptive effectiveness over time or compared with persons without solid organ transplantation No evidence was identified for Cu-IUD (Supplementary Appendix, https://stacks.cdc.gov/view/cdc/156516).
a. No graft failure	1	1	1	1	
b. Graft failure	2	1	2	1	
**Drug Interactions**					
**Antiretrovirals used for prevention (PrEP) or treatment of HIV**	Initiation	Continuation	Initiation	Continuation	**Clarification:** No known interaction exists between ARV therapy and IUD use. However, for persons with HIV infection, IUD placement is classified as category 2 if the person is not clinically well or not receiving ARV therapy. Otherwise, both placement and continuation are classified as category 1 (see recommendations for HIV infection). For persons at high risk for HIV infection, IUDs are category 1 for initiation and continuation (see recommendations for High risk for HIV infection).
a. Nucleoside reverse transcriptase inhibitors (NRTIs)					
i. Abacavir (ABC)	1/2	1	1/2	1	
ii. Tenofovir (TDF)	1/2	1	1/2	1	
iii. Zidovudine (AZT)	1/2	1	1/2	1	
iv. Lamivudine (3TC)	1/2	1	1/2	1	
v. Didanosine (DDI)	1/2	1	1/2	1	
vi. Emtricitabine (FTC)	1/2	1	1/2	1	
vii. Stavudine (D4T)	1/2	1	1/2	1	
b. Nonnucleoside reverse transcriptase inhibitors (NNRTIs)					
i. Efavirenz (EFV)	1/2	1	1/2	1	
ii. Etravirine (ETR)	1/2	1	1/2	1	
iii. Nevirapine (NVP)	1/2	1	1/2	1	
iv. Rilpivirine (RPV)	1/2	1	1/2	1	
c. Ritonavir-boosted protease inhibitors					
i. Ritonavir-boosted atazanavir (ATV/r)	1/2	1	1/2	1	
ii. Ritonavir-boosted darunavir (DRV/r)	1/2	1	1/2	1	
iii. Ritonavir-boosted fosamprenavir (FPV/r)	1/2	1	1/2	1	
iv. Ritonavir-boosted lopinavir (LPV/r)	1/2	1	1/2	1	
v. Ritonavir-boosted saquinavir (SQV/r)	1/2	1	1/2	1	
vi. Ritonavir-boosted tipranavir (TPV/r)	1/2	1	1/2	1	
d. Protease inhibitors without ritonavir					
i. Atazanavir (ATV)	1/2	1	1/2	1	
ii. Fosamprenavir (FPV)	1/2	1	1/2	1	
iii. Indinavir (IDV)	1/2	1	1/2	1	
iv. Nelfinavir (NFV)	1/2	1	1/2	1	
e. CCR5 co-receptor antagonists					
i. Maraviroc (MVC)	1/2	1	1/2	1	
f. HIV integrase strand transfer inhibitors					
i. Raltegravir (RAL)	1/2	1	1/2	1	
ii. Dolutegravir (DTG)	1/2	1	1/2	1	
iii. Elvitegravir (EVG)	1/2	1	1/2	1	
g. Fusion inhibitors					
i. Enfuvirtide	1/2	1	1/2	1	
**Anticonvulsant therapy**					
a. Certain anticonvulsants (phenytoin, carbamazepine, barbiturates, primidone, topiramate, and oxcarbazepine)	1		1		**Evidence:** Limited evidence suggests use of certain anticonvulsants does not interfere with the contraceptive effectiveness of the LNG-IUD (*137**,**138*).
b. Lamotrigine	1		1		**Evidence:** No drug interactions have been reported among women with epilepsy who are receiving lamotrigine and using the LNG-IUD (*138**,**139*).
**Antimicrobial therapy**					
a. Broad-spectrum antibiotics	1		1		—
b. Antifungals	1		1		—
c. Antiparasitics	1		1		—
d. Rifampin or rifabutin therapy	1		1		**Evidence:** One cross-sectional survey found that rifabutin had no impact on the effectiveness of the LNG-IUD (*137*).
**Psychotropic medications**					
a. Selective serotonin reuptake inhibitors (SSRIs)	1		1		**Comment:** For many common psychotropic agents, limited or no theoretical concern exists for clinically significant drug interactions when co-administered with hormonal contraceptives. However, either no or very limited data exist examining potential interactions for these classes of medications.
**St. John’s wort**	1		1		—

**Abbreviations:** ARV = antiretroviral; BMI = body mass index; CHC = combined hormonal contraceptive; CKD = chronic kidney disease; COC = combined oral contraceptive; Cu-IUD = copper intrauterine device; DMPA = depot medroxyprogesterone acetate; DVT = deep venous thrombosis; hCG = human chorionic gonadotropin; HDL = high-density lipoprotein; IBD = inflammatory bowel disease; IUD = intrauterine device; LDL = low-density lipoprotein; LNG = levonorgestrel; LNG-IUD = levonorgestrel intrauterine device; NA = not applicable; PE = pulmonary embolism; PID = pelvic inflammatory disease; POC = progestin-only contraceptive; PrEP = pre-exposure prophylaxis; RCT = randomized clinical trial; SLE = systemic lupus erythematous; STI = sexually transmitted infection; TOA = tubo-ovarian abscess; U.S. MEC = *U.S. Medical Eligibility Criteria for Contraceptive Use*; U.S. SPR = *U.S. Selected Practice Recommendations for Contraceptive Use*; VTE = venous thromboembolism.

## Appendix C Classifications for Progestin-Only Contraceptives

Classifications for progestin-only contraceptives (POCs) include those for progestin-only implants (68 mg etonogestrel), progestin-only injectables (depot medroxyprogesterone acetate [DMPA], 150 mg intramuscular [DMPA-IM] or 104 mg subcutaneous [DMPA-SC]), and progestin-only pills (POPs) (containing norethindrone, norgestrel, or drospirenone [DRSP]) (Box C1) (Table C1). DMPA-SC can be administered by a health care provider or through self-administration. Recommendations in this report and *U.S. Selected Practice Recommendations for Contraceptive Use, 2024* (*1*) for provider-administered DMPA (IM or SC) also apply to self-administered DMPA-SC. POCs do not protect against sexually transmitted infections (STIs), including HIV infection, and patients using POCs should be counseled that consistent and correct use of external (male) latex condoms reduces the risk for STIs, including HIV infection (*2*). Use of internal (female) condoms can provide protection from transmission of STIs, although data are limited (*2*). Patients also should be counseled that pre-exposure prophylaxis, when taken as prescribed, is highly effective for preventing HIV infection (*3*).

BOX C1 Categories for classifying progestin-only contraceptives U.S. MEC 1 = A condition for which there is no restriction for the use of the contraceptive method U.S. MEC 2 = A condition for which the advantages of using the method generally outweigh the theoretical or proven risks U.S. MEC 3 = A condition for which the theoretical or proven risks usually outweigh the advantages of using the method U.S. MEC 4 = A condition that represents an unacceptable health risk if the contraceptive method is used **Abbreviation:** U.S. MEC = *U.S. Medical Eligibility Criteria for Contraceptive Use*.

**TABLE C1:** Classifications for progestin-only contraceptives, including implants, depot medroxyprogesterone acetate, and progestin-only pills

Condition	Category						Clarification/Evidence/Comment
	Implant		DMPA		POP		
**Personal Characteristics and Reproductive History**							
**Pregnancy**	NA		NA		NA		**Clarification:** Use of POCs is not required. No known harm to the patient, the course of pregnancy, or the fetus occurs if POCs are inadvertently used during pregnancy. However, the relation between DMPA use during pregnancy and its effects on the fetus remains unclear.
**Age**							**Evidence:** Most studies have found that women lose BMD during DMPA use but recover BMD after discontinuation (*4*). Limited evidence demonstrates a weak association with fracture. However, one large study suggests that women who choose DMPA might be at higher risk for fracture before initiation (*5*). It is unclear whether adult women with long durations of DMPA use can regain BMD to baseline levels before entering menopause and whether adolescents can reach peak bone mass after discontinuation of DMPA. The relation between these changes in BMD during the reproductive years and future fracture risk is unknown. Studies generally find no effect of POCs other than DMPA on BMD (*4**–**52*)*.*
a. Menarche to <18 years	1		2		1		
b. 18–45 years	1		1		1		
c. >45 years	1		2		1		
**Parity**							
a. Nulliparous	1		1		1		—
b. Parous	1		1		1		—
**Breastfeeding**							
a. <21 days postpartum	2		2		2		**Clarification (breastfeeding):** Breastfeeding provides important health benefits for breastfeeding parent and infant. The U.S. Dietary Guidelines for Americans and American Academy of Pediatrics recommend that infants be exclusively breastfed for about the first 6 months with continued breastfeeding while introducing appropriate complementary foods for 1 year or longer (*53*) or up to age 2 years or longer (*54*). **Evidence (breastfeeding):** Two small, RCTs found no adverse impact on breastfeeding with initiation of etonogestrel implants within 48 hours postpartum. Other studies found that initiation of POPs, injectables, and implants at ≤6 weeks postpartum compared with nonhormonal use had no detrimental effect on breastfeeding outcomes or infant health, growth, and development in the first year postpartum. In general, these studies are of poor quality, lack standard definitions of breastfeeding or outcome measures, and have not included premature or ill infants (*55**,**56*). **Evidence:** Limited evidence suggests that DMPA use might further elevate risk for VTE among postpartum women compared with non-use (Supplementary Appendix, https://stacks.cdc.gov/view/cdc/156516). **Comment:** Risk factors for breastfeeding difficulties include previous breastfeeding difficulties, certain medical conditions, certain perinatal complications, and preterm birth. For all breastfeeding persons, with or without breastfeeding difficulties, discussions about contraception should include information about risks, benefits, and alternatives.
b. 21 to <30 days postpartum							**Clarification (breastfeeding):** Breastfeeding provides important health benefits for breastfeeding parent and infant. The U.S. Dietary Guidelines for Americans and American Academy of Pediatrics recommend that infants be exclusively breastfed for about the first 6 months with continued breastfeeding while introducing appropriate complementary foods for 1 year or longer (*53*) or up to age 2 years or longer (*54*). **Evidence (breastfeeding):** Two small, RCTs found no adverse impact on breastfeeding with initiation of etonogestrel implants within 48 hours postpartum. Other studies found that initiation of POPs, injectables, and implants at ≤6 weeks postpartum compared with nonhormonal use had no detrimental effect on breastfeeding outcomes or infant health, growth, and development in the first year postpartum. In general, these studies are of poor quality, lack standard definitions of breastfeeding or outcome measures, and have not included premature or ill infants (*55**,**56*). **Evidence:** Limited evidence suggests that DMPA use might further elevate risk for VTE among postpartum women compared with nonuse (Supplementary Appendix, https://stacks.cdc.gov/view/cdc/156516). **Comment:** Risk factors for breastfeeding difficulties include previous breastfeeding difficulties, certain medical conditions, certain perinatal complications, and preterm birth. For all breastfeeding persons, with or without difficulties, discussions about contraception should include information about risks, benefits, and alternatives.
i. With other risk factors for VTE (e.g., age ≥35 years, previous VTE, thrombophilia, immobility, transfusion at delivery, peripartum cardiomyopathy, BMI ≥30 kg/m^2^, postpartum hemorrhage, postcesarean delivery, preeclampsia, or smoking)	2		2		2		
ii. Without other risk factors for VTE	2		2		2		
c. 30–42 days postpartum							**Clarification (breastfeeding):** Breastfeeding provides important health benefits for breastfeeding parent and infant. The U.S. Dietary Guidelines for Americans and American Academy of Pediatrics recommend that infants be exclusively breastfed for about the first 6 months with continued breastfeeding while introducing appropriate complementary foods for 1 year or longer (*53*) or up to age 2 years or longer (*54*). **Evidence (breastfeeding):** Two small, RCTs found no adverse impact on breastfeeding with initiation of etonogestrel implants within 48 hours postpartum. Other studies found that initiation of POPs, injectables, and implants at ≤6 weeks postpartum compared with nonhormonal use had no detrimental effect on breastfeeding outcomes or infant health, growth, and development in the first year postpartum. In general, these studies are of poor quality, lack standard definitions of breastfeeding or outcome measures, and have not included premature or ill infants (*55**,**56*). **Evidence**: Limited evidence suggests that DMPA use might further elevate risk for VTE among postpartum women compared with nonuse (Supplementary Appendix, https://stacks.cdc.gov/view/cdc/156516). **Comment:** Risk factors for breastfeeding difficulties include previous breastfeeding difficulties, certain medical conditions, certain perinatal complications, and preterm birth. For all breastfeeding persons, with or without breastfeeding difficulties, discussions about contraception should include information about risks, benefits, and alternatives.
i. With other risk factors for VTE (e.g., age ≥35 years, previous VTE, thrombophilia, immobility, transfusion at delivery, peripartum cardiomyopathy, BMI ≥30 kg/m^2^, postpartum hemorrhage, postcesarean delivery, preeclampsia, or smoking)	1		2		1		
ii. Without other risk factors for VTE	1		1		1		
d. >42 days postpartum	1		1		1		**Clarification (breastfeeding):** Breastfeeding provides important health benefits for breastfeeding parent and infant. The U.S. Dietary Guidelines for Americans and American Academy of Pediatrics recommend that infants be exclusively breastfed for about the first 6 months with continued breastfeeding while introducing appropriate complementary foods for 1 year or longer (*53*) or up to age 2 years or longer (*54*). **Evidence:** Overall, studies found that initiation of POPs, injectables, and implants at >6 weeks postpartum compared with nonhormonal use had no detrimental effect on breastfeeding outcomes or infant health, growth, and development in the first year postpartum. In general, these studies are of poor quality, lack standard definitions of breastfeeding or outcome measures, and have not included premature or ill infants (*56*). **Comment:** Risk factors for breastfeeding difficulties include previous breastfeeding difficulties, certain medical conditions, certain perinatal complications, and preterm birth. For all breastfeeding persons, with or without breastfeeding difficulties, discussions about contraception should include information about risks, benefits, and alternatives.
**Postpartum** (nonbreastfeeding)							
a. <21 days postpartum	1		2		1		**Evidence:** Limited evidence suggests that DMPA use might further elevate risk for VTE among postpartum women compared with nonuse (Supplementary Appendix, https://stacks.cdc.gov/view/cdc/156516).
b. 21–42 days postpartum							
i. With other risk factors for VTE (e.g., age ≥35 years, previous VTE, thrombophilia, immobility, transfusion at delivery, peripartum cardiomyopathy, BMI ≥30 kg/m^2^, postpartum hemorrhage, postcesarean delivery, preeclampsia, or smoking)	1		2		1		**Evidence:** Limited evidence suggests that DMPA use might further elevate risk for VTE among postpartum women compared with nonuse (Supplementary Appendix, https://stacks.cdc.gov/view/cdc/156516).
ii. Without other risk factors for VTE	1		1		1		—
c. >42 days postpartum	1		1		1		—
**Postabortion **(spontaneous or induced)							
a. First trimester abortion							**Clarification:** POCs may be started immediately after abortion completion or at time of medication abortion initiation. **Clarification (DMPA):** After a first trimester medication abortion that did not include mifepristone, there is no restriction for the use of DMPA (category 1). After a first trimester medication abortion that included mifepristone, there is no restriction for use of DMPA after abortion completion (category 1) and benefits generally outweigh risks with DMPA use immediately at time of medication abortion initiation (category 2). Concurrent administration of DMPA with mifepristone might slightly decrease medication abortion effectiveness and increase risk for ongoing pregnancy. Risk for ongoing pregnancy with concurrent administration of DMPA with mifepristone should be considered along with personal preference and access to follow-up abortion and contraceptive care. **Evidence:** Limited evidence suggests decreased first trimester medication abortion effectiveness with concurrent administration of DMPA with mifepristone (immediate) versus DMPA administration after abortion completion (delayed). In one study, the risk for ongoing pregnancy, while overall low, was higher with immediate (3.6%) versus delayed (0.9%) DMPA administration (difference 2.7%; 90% CI = 0.4–5.6%) (*57*). This difference was not seen with other progestin-only methods (*58*). Evidence suggests that there is no increased risk for adverse events when POCs are initiated after first trimester procedural or medication abortion (immediately or delayed) (*58*) (Supplementary Appendix, https://stacks.cdc.gov/view/cdc/156516).
i. Procedural (surgical)	1		1		1		
ii. Medication	1		1/2		1		
iii. Spontaneous abortion with no intervention	1		1		1		
b. Second trimester abortion							**Clarification:** POCs may be started immediately after abortion completion or at time of medication abortion initiation.
i. Procedural (surgical)	1		1		1		
ii. Medication	1		1		1		
iii. Spontaneous abortion with no intervention	1		1		1		
c. Immediate postseptic abortion	1		1		1		**Clarification:** POCs may be started immediately after abortion completion or at time of medication abortion initiation.
**Past ectopic pregnancy**	1		1		2		**Comment:** POP users have a higher absolute rate of ectopic pregnancy than do users of other POCs but still lower than those using no method.
**History of pelvic surgery**	1		1		1		—
**Smoking**							
a. Age <35 years	1		1		1		—
b. Age ≥35 years							
i. <15 cigarettes per day	1		1		1		—
ii. ≥15 cigarettes per day	1		1		1		—
**Obesity**							
a. BMI ≥30 kg/m^2^	1		1		1		—
b. Menarche to <18 years and BMI ≥30 kg/m^2^	1		2		1		**Evidence:** Among adult women, generally no association has been found between baseline weight and weight gain among DMPA users compared with nonusers. Evidence is mixed for adolescent DMPA users, with certain studies observing greater weight gain among users with obesity compared with those without obesity but other studies demonstrating no association; methodologic differences across studies might account for the differences in findings. Data on other POC methods and other adverse outcomes including weight gain are limited (*59**–**76*).
**History of bariatric surgery** This condition is associated with increased risk for adverse health events as a result of pregnancy (Box 3).							
a. Restrictive procedures: decrease storage capacity of the stomach (vertical banded gastroplasty, laparoscopic adjustable gastric band, or laparoscopic sleeve gastrectomy)	1		1		1		**Evidence:** Limited evidence demonstrated no substantial decrease in effectiveness of oral contraceptives among women who underwent laparoscopic placement of an adjustable gastric band (*77*).
b. Malabsorptive procedures: decrease absorption of nutrients and calories by shortening the functional length of the small intestine (Roux-en-Y gastric bypass or biliopancreatic diversion)	1		1		3		**Evidence:** Limited evidence demonstrated no substantial decrease in effectiveness of oral contraceptives among women who underwent a biliopancreatic diversion; however, evidence from pharmacokinetic studies suggested conflicting results regarding oral contraceptive effectiveness among women who underwent a jejunoileal bypass (*77*). **Comment:** Bariatric surgical procedures involving a malabsorptive component have the potential to decrease oral contraceptive effectiveness, perhaps further decreased by postoperative complications such as long-term diarrhea, vomiting, or both.
**Surgery**							
a. Minor surgery without immobilization	1		1		1		—
b. Major surgery							
i. Without prolonged immobilization	1		1		1		—
ii. With prolonged immobilization	1		2		1		**Evidence:** No direct evidence was identified on risk for thrombosis with POC use among those undergoing major surgery. Use of DMPA, which has been associated with a higher risk for venous thrombosis compared with nonuse (Supplementary Appendix, https://stacks.cdc.gov/view/cdc/156516), might further elevate risk for thrombosis among persons with prolonged immobilization after major surgery.
**Cardiovascular Disease**							
**Multiple risk factors for atherosclerotic cardiovascular disease** (e.g., older age, smoking, diabetes, hypertension, low HDL, high LDL, or high triglyceride levels)	2		3		2		**Clarification:** When multiple major risk factors exist, risk for cardiovascular disease might increase substantially. Certain POCs might increase the risk for thrombosis, although this increase is substantially less than with COCs. The effects of DMPA might persist for some time after discontinuation. **Clarification:** The recommendations apply to known pre-existing medical conditions or characteristics. Few if any screening tests are needed before initiation of contraception. See U.S. SPR (https://www.cdc.gov/contraception/hcp/usspr) (*1*).
**Hypertension** Systolic blood pressure ≥160 mm Hg or diastolic blood pressure ≥100 mm Hg are associated with increased risk for adverse health events as a result of pregnancy (Box 3).							
a. Adequately controlled hypertension	1		2		1		**Clarification:** For all categories of hypertension, classifications are based on the assumption that no other risk factors exist for cardiovascular disease. When multiple risk factors do exist, risk for cardiovascular disease might increase substantially. A single reading of blood pressure level is not sufficient to classify a person as hypertensive. **Clarification:** Persons adequately treated for hypertension are at lower risk for acute myocardial infarction and stroke than are untreated persons. Although no data exist, POC users with adequately controlled and monitored hypertension should be at lower risk for acute myocardial infarction and stroke than are untreated hypertensive POC users.
b. Elevated blood pressure levels (properly taken measurements)							**Clarification:** For all categories of hypertension, classifications are based on the assumption that no other risk factors exist for cardiovascular disease. When multiple risk factors do exist, risk for cardiovascular disease might increase substantially. A single reading of blood pressure level is not sufficient to classify a person as hypertensive. **Evidence:** Limited evidence suggests that among women with hypertension, those who used POPs or progestin-only injectables had a small increased risk for cardiovascular events compared with women who did not use these methods (*78*).
i. Systolic 140–159 mm Hg or diastolic 90–99 mm Hg	1		2		1		
ii. Systolic ≥160 mm Hg or diastolic ≥100 mm Hg	2		3		2		
c. Vascular disease	2		3		2		**Clarification:** For all categories of hypertension, classifications are based on the assumption that no other risk factors exist for cardiovascular disease. When multiple risk factors do exist, risk for cardiovascular disease might increase substantially. A single reading of blood pressure level is not sufficient to classify a person as hypertensive. **Comment:** Concern exists about hypoestrogenic effects and reduced HDL levels, particularly among users of DMPA. However, little concern exists about these effects with regard to POPs. The effects of DMPA might persist for some time after discontinuation.
**History of high blood pressure during pregnancy** (when current blood pressure is measurable and normal)	1		1		1		—
**Deep venous thrombosis/ Pulmonary embolism** This condition is associated with increased risk for adverse health events as a result of pregnancy (Box 3).							
a. Current or history of DVT/PE, receiving anticoagulant therapy (therapeutic dose) (e.g., acute DVT/PE or long-term therapeutic dose)	2		2		2		**Clarification**: Persons using anticoagulant therapy are at risk for gynecologic complications of therapy, such as heavy or prolonged bleeding and hemorrhagic ovarian cysts. POCs can be of benefit in preventing or treating these complications; benefits might vary by POC dose and formulation. When a contraceptive method is used as a therapy, rather than solely to prevent pregnancy, the risk/benefit ratio might differ and should be considered on a case-by-case basis. **Evidence**: Limited evidence was identified on use of POCs among women with acute DVT/PE receiving anticoagulant therapy (Supplementary Appendix, https://stacks.cdc.gov/view/cdc/156516 In one study among women with a history of acute VTE currently receiving therapeutic anticoagulant therapy (i.e., rivaroxaban or enoxaparin/vitamin K antagonist [warfarin or acenocoumarol]), the incidence of recurrent VTE was similar among estrogen users (CHC or estrogen-only pills), POC users, and women not on hormonal therapy (*79*). Limited evidence suggests that intramuscular injections of DMPA in women receiving chronic anticoagulation therapy do not pose a significant risk for hematoma at the injection site or increase the risk for heavy or irregular vaginal bleeding (*80*).
b. History of DVT/PE, receiving anticoagulant therapy (prophylactic dose)							**Clarification:** Persons using anticoagulant therapy are at risk for gynecologic complications of therapy, such as heavy or prolonged bleeding and hemorrhagic ovarian cysts. POCs can be of benefit in preventing or treating these complications; benefits might vary by POC dose and formulation. When a contraceptive method is used as a therapy, rather than solely to prevent pregnancy, the risk/benefit ratio might differ and should be considered on a case-by-case basis. **Evidence**: Limited evidence was identified on use of POCs among women with acute DVT/PE receiving anticoagulant therapy (Supplementary Appendix, https://stacks.cdc.gov/view/cdc/156516). Use of DMPA, which has been associated with a higher risk for venous thrombosis compared with nonuse (Supplementary Appendix, https://stacks.cdc.gov/view/cdc/156516), might further elevate risk for thrombosis among persons with a history of DVT/PE and at higher risk for recurrent DVT/PE. Limited evidence suggests that intramuscular injections of DMPA in women receiving chronic anticoagulation therapy do not pose a significant risk for hematoma at the injection site or increase the risk for heavy or irregular vaginal bleeding (*80*).
i. Higher risk for recurrent DVT/PE (one or more risk factors)	2		3		2		
**•** Thrombophilia (e.g., factor V Leiden mutation; prothrombin gene mutation; protein S, protein C, and antithrombin deficiencies; or antiphospholipid syndrome) • Active cancer (metastatic, receiving therapy, or within 6 months after clinical remission), excluding nonmelanoma skin cancer • History of recurrent DVT/PE							
ii. Lower risk for recurrent DVT/PE (no risk factors)	2		2		2		
c. History of DVT/PE, not receiving anticoagulant therapy							
i. Higher risk for recurrent DVT/PE (one or more risk factors)	2		3		2		**Evidence:** Use of DMPA, which has been associated with a higher risk for venous thrombosis compared with nonuse (Supplementary Appendix, https://stacks.cdc.gov/view/cdc/156516), might further elevate risk for thrombosis among persons with a history of DVT/PE and at higher risk for recurrent DVT/PE.
• History of estrogen-associated DVT/PE • Pregnancy-associated DVT/PE • Idiopathic DVT/PE • Thrombophilia (e.g., factor V Leiden mutation; prothrombin gene mutation; protein S, protein C, and antithrombin deficiencies; or antiphospholipid syndrome) • Active cancer (metastatic, receiving therapy, or within 6 months after clinical remission), excluding nonmelanoma skin cancer • History of recurrent DVT/PE							
ii. Lower risk for recurrent DVT/PE (no risk factors)	2		2		2		—
d. Family history (first-degree relatives)	1		1		1		—
**Thrombophilia** (e.g., factor V Leiden mutation; prothrombin gene mutation; protein S, protein C, and antithrombin deficiencies; or antiphospholipid syndrome) This condition is associated with increased risk for adverse health events as a result of pregnancy (Box 3).	2		3		2		**Clarification:** Routine screening in the general population before contraceptive initiation is not recommended. **Clarification**: If a person has current or history of DVT/PE, see recommendations for DVT/PE. **Clarification**: Classification of antiphospholipid syndrome includes presence of a clinical feature (e.g., thrombosis or obstetric morbidity) and persistently abnormal antiphospholipid antibody test on two or more occasions at least 12 weeks apart (*81*). **Evidence:** Among women with factor V Leiden mutation, one study found that women using POCs had an increased risk for venous thrombosis compared with non-users without the mutation, with the highest relative risk for DMPA users (*82*). Women with prothrombin gene mutation using POCs did not have an increased risk for venous thrombosis compared with nonusers without the mutation (*82*). No evidence was identified on POC use among persons with protein S deficiency, protein C deficiency, antithrombin deficiency, or antiphospholipid syndrome (Supplementary Appendix, https://stacks.cdc.gov/view/cdc/156516).
**Superficial venous disorders**							
a. Varicose veins	1		1		1		—
b. Superficial venous thrombosis (acute or history)	1		2		1		**Evidence:** No direct evidence was identified on risk for thrombosis with POC use among persons with superficial venous thrombosis (Supplementary Appendix, https://stacks.cdc.gov/view/cdc/156516). Persons with superficial venous thrombosis are at higher risk for venous thrombosis than the general population (*83*). Use of DMPA, which has been associated with a higher risk for venous thrombosis compared with non-use (Supplementary Appendix, https://stacks.cdc.gov/view/cdc/156516), might further elevate risk for thrombosis among persons with acute or history of superficial venous thrombosis.
**Current and history of ischemic heart disease** This condition is associated with increased risk for adverse health events as a result of pregnancy (Box 3).	Initiation	Continuation			Initiation	Continuation	**Comment:** Concern exists about hypoestrogenic effects and reduced HDL levels, particularly among users of DMPA. However, little concern exists about these effects with regard to POPs. The effects of DMPA might persist for some time after discontinuation.
	2	3	3		2	3	
**Stroke** (history of cerebrovascular accident) This condition is associated with increased risk for adverse health events as a result of pregnancy (Box 3).	Initiation	Continuation			Initiation	Continuation	**Comment:** Concern exists about hypoestrogenic effects and reduced HDL levels, particularly among users of DMPA. However, little concern exists about these effects with regard to POPs. The effects of DMPA might persist for some time after discontinuation.
	2	3	3		2	3	
**Valvular heart disease** Complicated valvular heart disease is associated with increased risk for adverse health events as a result of pregnancy (Box 3).							
a. Uncomplicated	1		1		1		—
b. Complicated (pulmonary hypertension, risk for atrial fibrillation, or history of subacute bacterial endocarditis)	1		2		1		**Evidence:** No direct evidence was identified on risk for thrombosis with POC use among persons with valvular heart disease (Supplementary Appendix, https://stacks.cdc.gov/view/cdc/156516). Use of DMPA, which has been associated with a higher risk for venous thrombosis compared with nonuse (Supplementary Appendix, https://stacks.cdc.gov/view/cdc/156516), might further elevate risk for thrombosis among persons with complicated valvular heart disease.
**Peripartum cardiomyopathy** This condition is associated with increased risk for adverse health events as a result of pregnancy (Box 3).							**Evidence:** No direct evidence was identified on the safety of POC use among persons with peripartum cardiomyopathy (Supplementary Appendix, https://stacks.cdc.gov/view/cdc/156516). Limited indirect evidence from noncomparative studies of women with cardiac disease demonstrated few cases of hypertension, thromboembolism, and heart failure in women with cardiac disease using POPs and DMPA (*84*). Use of DMPA, which has been associated with a higher risk for venous thrombosis compared with nonuse (Supplementary Appendix, https://stacks.cdc.gov/view/cdc/156516), might further elevate risk for thrombosis among persons with peripartum cardiomyopathy. **Comment:** Progestin-only implants might induce cardiac arrhythmias in healthy persons; persons with peripartum cardiomyopathy have a high incidence of cardiac arrhythmias.
a. Normal or mildly impaired cardiac function (New York Heart Association Functional Class I or II: no limitation of activities or slight, mild limitation of activity) (*85*)							
i. <6 months	1		2		1		
ii. ≥6 months	1		2		1		
b. Moderately or severely impaired cardiac function (New York Heart Association Functional Class III or IV: marked limitation of activity or should be at complete rest) (*85*)	2		3		2		
**Renal Disease**							
**Chronic kidney disease** This condition is associated with increased risk for adverse health events as a result of pregnancy (Box 3).							
a. Current nephrotic syndrome	2		3		2 DRSP POP with known hyperkalemia: 4		**Clarification (DRSP POP):** Persons with known hyperkalemia should not use DRSP POPs because of the risk for worsening hyperkalemia (category 4). For persons with CKD without known hyperkalemia (category 2), consider checking serum potassium level during first cycle of DRSP POPs. **Evidence:** No direct evidence was identified on POC use among persons with CKD with current nephrotic syndrome (Supplementary Appendix, https://stacks.cdc.gov/view/cdc/156516). Persons with severe CKD or nephrotic syndrome are at higher risk for thrombosis than the general population (*86**–**90*). Use of DMPA, which has been associated with increased risk for thrombosis compared with nonuse (Supplementary Appendix, https://stacks.cdc.gov/view/cdc/156516), might further elevate risk for thrombosis among those with CKD with current nephrotic syndrome. Persons with severe CKD have a higher prevalence of fracture than the general population (*91**–**93*). Use of DMPA, which has been associated with small changes in bone mineral density (*4*) might further elevate risk for fracture among persons with CKD with current nephrotic syndrome. **Comment:** A person might have CKD without current nephrotic syndrome, but might have other conditions often associated with CKD (e.g., diabetes, hypertension, SLE). See recommendations for other conditions if they apply.
b. Hemodialysis	2		3		2 DRSP POP with known hyperkalemia: 4		**Clarification (DRSP POP):** Persons with known hyperkalemia should not use DRSP POPs because of the risk for worsening hyperkalemia (category 4). For persons with CKD without known hyperkalemia (category 2), consider checking serum potassium level during first cycle of DRSP POPs. **Evidence:** No direct evidence was identified on POC use among persons with CKD on hemodialysis (Supplementary Appendix, https://stacks.cdc.gov/view/cdc/156516). Persons with CKD on dialysis are at higher risk for thrombosis than the general population (94-96). Use of DMPA, which has been associated with increased risk for thrombosis compared with nonuse (Supplementary Appendix, https://stacks.cdc.gov/view/cdc/156516), might further elevate risk for thrombosis among those with CKD on dialysis. Persons with CKD on dialysis have a higher prevalence of fracture than the general population (*97**–**99*). Use of DMPA, which has been associated with small changes in bone mineral density (*4*), might further elevate risk for fracture among persons with CKD on dialysis. **Comment:** A person might have CKD without hemodialysis, but might have other conditions often associated with CKD (e.g., diabetes, hypertension, SLE). See recommendations for other conditions if they apply.
c. Peritoneal dialysis	2		3		2 DRSP POP with known hyperkalemia: 4		**Clarification (DRSP POP):** Persons with known hyperkalemia should not use DRSP POPs because of the risk for worsening hyperkalemia (category 4). For persons with CKD without known hyperkalemia (category 2), consider checking serum potassium level during first cycle of DRSP POPs. **Evidence:** No direct evidence was identified on POC use among persons with CKD on peritoneal dialysis (Supplementary Appendix, https://stacks.cdc.gov/view/cdc/156516). Persons with CKD on dialysis are at higher risk for thrombosis than the general population (*94**–**96*). Use of DMPA, which has been associated with increased risk for thrombosis compared with nonuse (Supplementary Appendix, https://stacks.cdc.gov/view/cdc/156516), might further elevate risk for thrombosis among those with CKD on dialysis. Persons with CKD on dialysis have a higher prevalence of fracture than the general population (*97**–**99*). Use of DMPA, which has been associated with small changes in bone mineral density (*4*), might further elevate risk for fracture among persons with CKD on dialysis. **Comment:** A person might have CKD without peritoneal dialysis but might have other conditions often associated with CKD (e.g., diabetes, hypertension, and SLE). See recommendations for other conditions if they apply.
**Rheumatic Diseases**							
**Systemic lupus erythematosus** This condition is associated with increased risk for adverse health events as a result of pregnancy (Box 3).			Initiation	Continuation	—		
a. Positive (or unknown) antiphospholipid antibodies	2		3	3	2		**Clarification:** Persons with SLE are at increased risk for ischemic heart disease, stroke, and VTE. Categories assigned to such conditions in U.S. MEC should be the same for persons with SLE who have these conditions. For all subconditions of SLE, classifications are based on the assumption that no other risk factors for cardiovascular disease are present; these classifications must be modified in the presence of such risk factors (*100**–**118*). **Evidence:** No direct evidence was identified on POC use among persons with SLE with antiphospholipid antibodies (*119*) (Supplementary Appendix, https://stacks.cdc.gov/view/cdc/156516). Persons with SLE with antiphospholipid antibodies are at higher risk for thrombosis than the general population (*120**,**121*). Use of DMPA, which has been associated with a higher risk for venous thrombosis compared with nonuse (Supplementary Appendix, https://stacks.cdc.gov/view/cdc/156516), might further elevate risk for thrombosis among persons with SLE with antiphospholipid antibodies.
b. Severe thrombocytopenia	2		3	2	2		**Clarification:** Persons with SLE are at increased risk for ischemic heart disease, stroke, and VTE. Categories assigned to such conditions in U.S. MEC should be the same for persons with SLE who have these conditions. For all subconditions of SLE, classifications are based on the assumption that no other risk factors for cardiovascular disease are present; these classifications must be modified in the presence of such risk factors (*100**–**118*). **Comment:** Severe thrombocytopenia increases the risk for bleeding. POCs might be useful in treating heavy or prolonged bleeding in persons with severe thrombocytopenia. However, given the increased or erratic bleeding that might be seen on initiation of DMPA and its irreversibility for 11–13 weeks after administration, initiation of this method in persons with severe thrombocytopenia should be done with caution.
c. Immunosuppressive therapy	2		2	2	2		**Clarification:** Persons with SLE are at increased risk for ischemic heart disease, stroke, and VTE. Categories assigned to such conditions in U.S. MEC should be the same for persons with SLE who have these conditions. For all subconditions of SLE, classifications are based on the assumption that no other risk factors for cardiovascular disease are present; these classifications must be modified in the presence of such risk factors (*100**–**118*).
d. None of the above	2		2	2	2		**Clarification:** Persons with SLE are at increased risk for ischemic heart disease, stroke, and VTE. Categories assigned to such conditions in U.S. MEC should be the same for persons with SLE who have these conditions. For all subconditions of SLE, classifications are based on the assumption that no other risk factors for cardiovascular disease are present; these classifications must be modified in the presence of such risk factors (*100**–**118*).
**Rheumatoid arthritis**							
a. Not receiving immunosuppressive therapy	1		2		1		**Evidence:** Limited evidence demonstrates no consistent pattern of improvement or worsening of rheumatoid arthritis with use of oral contraceptives, progesterone, or estrogen (*122*).
b. Receiving immunosuppressive therapy	1		2/3		1		**Clarification (DMPA):** DMPA use among persons receiving long-term corticosteroid therapy with a history of, or with risk factors for, nontraumatic fractures is classified as category 3. Otherwise, DMPA use for persons with rheumatoid arthritis is classified as category 2. **Evidence:** Limited evidence demonstrates no consistent pattern of improvement or worsening of rheumatoid arthritis with use of oral contraceptives, progesterone, or estrogen (*122*).
**Neurologic Conditions**							
**Headaches**							
a. Nonmigraine (mild or severe)	1		1		1		—
b. Migraine							**Evidence:** No studies directly examined the risk for stroke among women with migraine using POCs (*123*). Limited evidence demonstrated that women using POPs, DMPA, or implants do not have an increased risk for ischemic stroke compared with nonusers (*124*). **Comment:** Menstrual migraine is a subtype of migraine without aura. For more information, see the International Headache Society’s *International Classification of Headache Disorders, 3rd ed. *(https://ichd-3.org) (*125*).
i. Without aura (includes menstrual migraine)	1		1		1		
ii. With aura	1		1		1		
**Epilepsy** This condition is associated with increased risk for adverse health events as a result of pregnancy (Box 3).	1		1		1		**Clarification:** If a person is taking anticonvulsants, see recommendations for Drug Interactions. Certain anticonvulsants lower POC effectiveness.
**Multiple sclerosis**							**Evidence:** Limited evidence demonstrates that use of COCs or oral contraceptives (type not specified) among women with multiple sclerosis does not worsen the clinical course of disease (*126*). **Comment:** Persons with multiple sclerosis might have compromised bone health from disease-related disability, immobility, and use of corticosteroids. Use of DMPA, which has been associated with small changes in BMD, might be of concern.
a. Without prolonged immobility	1		2		1		
b. With prolonged immobility	1		2		1		
**Depressive Disorders**							
**Depressive disorders**	1		1		1		**Clarification:** If a person is taking psychotropic medications or St. John’s wort, see recommendations for Drug Interactions. **Evidence:** The frequency of psychiatric hospitalizations for women with bipolar disorder or depression did not significantly differ among women using DMPA, LNG-IUD, Cu-IUD, or sterilization (*127*).
**Reproductive Tract Infections and Disorders**							
**Vaginal bleeding patterns**							
a. Irregular pattern without heavy bleeding	2		2		2		**Comment:** Irregular menstrual bleeding patterns are common among healthy persons. POC use frequently induces an irregular bleeding pattern. Implant use might induce irregular bleeding patterns, especially during the first 3–6 months, although these patterns might persist longer.
b. Heavy or prolonged bleeding (includes regular and irregular patterns)	2		2		2		**Clarification:** Unusually heavy bleeding should raise the suspicion of a serious underlying condition.
**Unexplained vaginal bleeding** (suspicious for serious condition) before evaluation	3		3		2		**Clarification:** If pregnancy or an underlying pathological condition (e.g., pelvic malignancy) is suspected, it must be evaluated and the category adjusted after evaluation. **Comment:** POCs might cause irregular bleeding patterns, which might mask symptoms of underlying pathologic conditions. The effects of DMPA might persist for some time after discontinuation.
**Endometriosis**	1		1		1		—
**Benign ovarian tumors** (including cysts)	1		1		1		—
**Severe dysmenorrhea**	1		1		1		—
**Gestational trophoblastic disease** This condition is associated with increased risk for adverse health events as a result of pregnancy (Box 3).							**Clarification:** For all subconditions of gestational trophoblastic disease, classifications are based on the assumption that persons are under close medical supervision because of the need for monitoring of β-hCG levels for appropriate disease surveillance.
a. Suspected gestational trophoblastic disease (immediate postevacuation)							
i. Uterine size first trimester	1		1		1		
ii. Uterine size second trimester	1		1		1		
b. Confirmed gestational trophoblastic disease (after initial evacuation and during monitoring)							
i. Undetectable or nonpregnant β–hCG levels	1		1		1		
ii. Decreasing β–hCG levels	1		1		1		
iii. Persistently elevated β-hCG levels or malignant disease, with no evidence or suspicion of intrauterine disease	1		1		1		
iv. Persistently elevated β-hCG levels or malignant disease, with evidence or suspicion of intrauterine disease	1		1		1		
**Cervical ectropion**	1		1		1		—
**Cervical intraepithelial neoplasia**	2		2		1		**Evidence:** Among women with persistent human papillomavirus infection, long-term DMPA use (≥5 years) might increase the risk for carcinoma in situ and invasive carcinoma (*128*).
**Cervical cancer** (awaiting treatment)	2		2		1		**Comment:** Theoretical concern exists that POC use might affect prognosis of the existing disease. While awaiting treatment, POCs may be used. In general, treatment of this condition can render a person infertile.
**Breast disease** Breast cancer is associated with increased risk for adverse health events as a result of pregnancy (Box 3).							
a. Undiagnosed mass	2		2		2		**Clarification:** Evaluation of mass should be pursued as early as possible.
b. Benign breast disease	1		1		1		—
c. Family history of cancer	1		1		1		—
d. Breast cancer							
i. Current	4		4		4		**Comment:** Breast cancer is a hormonally sensitive tumor, and the prognosis for persons with current or recent breast cancer might worsen with POC use.
ii. Past and no evidence of current disease for 5 years	3		3		3		
**Endometrial hyperplasia**	1		1		1		—
**Endometrial cancer** This condition is associated with increased risk for adverse health events as a result of pregnancy (Box 3).	1		1		1		**Comment:** While awaiting treatment, POCs may be used. In general, treatment of this condition renders a person infertile.
**Ovarian cancer** This condition is associated with increased risk for adverse health events as a result of pregnancy (Box 3).	1		1		1		**Comment:** While awaiting treatment, POCs may be used. In general, treatment of this condition renders a person infertile.
**Uterine fibroids**	1		1		1		**Comment:** POCs do not appear to cause growth of uterine fibroids.
**Pelvic inflammatory disease**							**Comment:** Whether POCs, like COCs, reduce the risk for PID among persons with STIs is unknown; however, they do not protect against HIV infection or lower genital tract STIs.
a. Current PID	1		1		1		
b. Past PID							
i. With subsequent pregnancy	1		1		1		
ii. Without subsequent pregnancy	1		1		1		
**Sexually transmitted infections**							
a. Current purulent cervicitis or chlamydial infection or gonococcal infection	1		1		1		—
b. Vaginitis (including *Trichomonas vaginalis* and bacterial vaginosis)	1		1		1		—
c. Other factors related to STIs	1		1		1		—
**HIV**							
**High risk for HIV infection**	1		1		1		**Evidence:** High-quality evidence from one RCT observed no statistically significant differences in HIV acquisition between DMPA-IM versus Cu-IUD, DMPA-IM versus LNG implant, and Cu-IUD versus LNG implant. Of the low-to-moderate-quality evidence from 14 observational studies, certain studies suggested a possible increased risk for HIV infection with progestin-only injectable use, which was most likely due to unmeasured confounding. Low-quality evidence from three observational studies did not suggest an increased HIV infection risk for implant users. No studies of sufficient quality were identified for POPs (*129**–**131*).
**HIV infection** For persons with HIV infection who are not clinically well or not receiving ARV therapy, this condition is associated with increased risk for adverse health events as a result of pregnancy (Box 3).	1		1		1		**Clarification:** Drug interactions might exist between hormonal contraceptives and ARV drugs (see recommendations for Drug Interactions). **Evidence:** Overall, evidence does not support an association between POC use and progression of HIV infection. Limited direct evidence on an association between POC use and transmission of HIV to noninfected partners, as well as studies measuring genital viral shedding as a proxy for infectivity, have had mixed results. Studies measuring whether hormonal contraceptive methods affect plasma HIV viral load generally have found no effect (*132**–**134*).
**Other Infections**							
**Schistosomiasis** Schistosomiasis with fibrosis of the liver is associated with increased risk for adverse health events as a result of pregnancy (Box 3).							
a. Uncomplicated	1		1		1		**Evidence:** Among women with uncomplicated schistosomiasis, limited evidence demonstrated that DMPA use had no adverse effects on liver function (*135*).
b. Fibrosis of the liver (if severe, see recommendations for Cirrhosis)	1		1		1		—
**Tuberculosis** This condition is associated with increased risk for adverse health events as a result of pregnancy (Box 3).							**Clarification:** If a person is taking rifampin, see recommendations for Drug Interactions. Rifampin is likely to decrease the effectiveness of certain POCs.
a. Nonpelvic	1		1		1		
b. Pelvic	1		1		1		
**Malaria**	1		1		1		—
**Endocrine Conditions**							
**Diabetes** Insulin-dependent diabetes; diabetes with nephropathy, retinopathy or neuropathy; diabetes with other vascular disease; or diabetes of >20 years’ duration are associated with increased risk for adverse health events as a result of pregnancy (Box 3).							
a. History of gestational disease	1		1		1		**Evidence:** POCs had no adverse effects on serum lipid levels in women with a history of gestational diabetes in two small studies (*136**,**137*). Limited evidence is inconsistent about the development of noninsulin-dependent diabetes among users of POCs with a history of gestational diabetes (*138**–**141*).
b. Nonvascular disease							**Evidence:** Among women with insulin-dependent or noninsulin-dependent diabetes, limited evidence on use of POCs (POPs, DMPA, and LNG implant) suggests that these methods have little effect on short-term or long-term diabetes control (e.g., glycosylated hemoglobin levels), hemostatic markers, or lipid profile (*142**–**145*).
i. Non-insulin dependent	2		2		2		
ii. Insulin dependent	2		2		2		
c. Nephropathy, retinopathy or neuropathy	2		3		2		**Comment:** Concern exists about hypoestrogenic effects and reduced HDL levels, particularly among users of DMPA. The effects of DMPA might persist for some time after discontinuation. Certain POCs might increase the risk for thrombosis, although this increase is substantially less than with COCs.
d. Other vascular disease or diabetes of >20 years’ duration	2		3		2		**Comment:** Concern exists about hypoestrogenic effects and reduced HDL levels, particularly among users of DMPA. The effects of DMPA might persist for some time after discontinuation. Certain POCs might increase the risk for thrombosis, although this increase is substantially less than with COCs.
**Thyroid disorders**							
a. Simple goiter	1		1		1		—
b. Hyperthyroid	1		1		1		—
c. Hypothyroid	1		1		1		—
**Gastrointestinal Conditions**							
**Inflammatory bowel disease** (ulcerative colitis or Crohn’s disease)	1		2		2		**Evidence:** Risk for disease relapse among women with IBD using oral contraceptives (most studies did not specify formulation) did not increase significantly from that for nonusers (*146*). **Comment:** Absorption of POPs among persons with IBD might be reduced if the person has substantial malabsorption caused by severe disease or small bowel surgery. Women with IBD have a higher prevalence of osteoporosis and osteopenia than the general population. Use of DMPA, which has been associated with small changes in BMD, might be of concern.
**Gallbladder disease**							
a. Asymptomatic	2		2		2		—
b. Symptomatic							
i. Current	2		2		2		—
ii. Treated by cholecystectomy	2		2		2		—
iii. Medically treated	2		2		2		—
**History of cholestasis**							
a. Pregnancy related	1		1		1		
b. Past COC related	2		2		2		**Comment:** Theoretical concern exists that a history of COC-related cholestasis might predict subsequent cholestasis with POC use. However, this has not been documented.
**Viral hepatitis**							
a. Acute or flare	1		1		1		**Evidence**: No direct evidence was identified on POC use among persons with viral hepatitis (Supplementary Appendix, https://stacks.cdc.gov/view/cdc/156516).
b. Chronic	1		1		1		**Evidence**: No evidence was identified on POC use among persons with viral hepatitis (Supplementary Appendix, https://stacks.cdc.gov/view/cdc/156516).
**Cirrhosis** Decompensated cirrhosis is associated with increased risk for adverse health events as a result of pregnancy (Box 3).							
a. Compensated (normal liver function)	1		1		1		**Evidence**: No direct evidence was identified on POC use among persons with cirrhosis (Supplementary Appendix, https://stacks.cdc.gov/view/cdc/156516).
b. Decompensated (impaired liver function)	2		3		2		**Evidence:** No direct evidence was identified on POC use among persons with cirrhosis. (Supplementary Appendix, https://stacks.cdc.gov/view/cdc/156516). DMPA use has been associated with a higher risk for venous thrombosis compared with nonuse (Supplementary Appendix, https://stacks.cdc.gov/view/cdc/156516). **Comment:** Hepatic metabolism of exogenous hormones might be impaired in persons with liver dysfunction, which could lead to increased progestin levels in circulation and progestin-related side effects and adverse events (e.g., thrombosis), which might vary by dose and formulation. Any progestin-related hepatotoxicity might be less tolerated in persons with existing liver dysfunction.
**Liver tumors** Hepatocellular adenoma and malignant liver tumors are associated with increased risk for adverse health events as a result of pregnancy (Box 3).							
a. Benign							
i. Focal nodular hyperplasia	2		2		2		**Evidence:** Limited evidence suggests that progestin use does not influence either progression or regression of focal nodular hyperplasia (Supplementary Appendix, https://stacks.cdc.gov/view/cdc/156516).
ii. Hepatocellular adenoma	2		3		2		**Evidence:** Limited evidence suggests that hepatocellular adenomas generally regress or remain stable during progestin use (Supplementary Appendix, https://stacks.cdc.gov/view/cdc/156516).
b. Malignant (hepatocellular carcinoma)	3		3		3		**Evidence:** No direct evidence was identified on POC use among persons with hepatocellular carcinoma (Supplementary Appendix, https://stacks.cdc.gov/view/cdc/156516).
**Respiratory Conditions**							
**Cystic fibrosis** This condition is associated with increased risk for adverse health events as a result of pregnancy (Box 3).	1		2		1		**Clarification:** Persons with cystic fibrosis are at increased risk for diabetes, liver disease, gallbladder disease, and VTE (particularly related to use of central venous catheters) and are frequently prescribed antibiotics. Categories assigned to such conditions in U.S. MEC should be the same for persons with cystic fibrosis who have these conditions. For cystic fibrosis, classifications are based on the assumption that no other conditions are present; these classifications must be modified in the presence of such conditions. **Clarification:** Certain drugs to treat cystic fibrosis (e.g., lumacaftor) might reduce effectiveness of hormonal contraceptives, including oral, injectable, transdermal, and implantable contraceptives. **Evidence:** Limited evidence suggests that use of COCs or oral contraceptives (type not specified) among women with cystic fibrosis is not associated with worsening of disease severity. Very limited evidence suggests that cystic fibrosis does not impair the effectiveness of hormonal contraception (*147*). **Comment:** Persons with cystic fibrosis have a higher prevalence of osteopenia, osteoporosis, and fragility fractures than the general population. Use of DMPA, which has been associated with small changes in BMD, might be of concern.
**Hematologic Conditions**							
**Thalassemia**	1		1		1		—
**Sickle cell disease** This condition is associated with increased risk for adverse health events as a result of pregnancy (Box 3).	1		2/3		1		**Clarification (DMPA):** The category should be assessed according to the severity of the condition and risk for thrombosis. **Evidence:** Limited evidence suggests that POC use does not increase risk for thrombosis among persons with sickle cell disease (Supplementary Appendix, https://stacks.cdc.gov/view/cdc/156516). Persons with sickle cell disease are at higher risk for stroke and venous thrombosis than the general population (*148**–**151*). Use of DMPA, which has been associated with a higher risk for venous thrombosis compared with nonuse (Supplementary Appendix, https://stacks.cdc.gov/view/cdc/156516), might further elevate risk for thrombosis among persons with sickle cell disease. POC might be beneficial in reducing clinical symptoms (e.g., pain crises) (Supplementary Appendix, https://stacks.cdc.gov/view/cdc/156516).
**Iron deficiency anemia**	1		1		1		**Comment:** Changes in the menstrual pattern associated with POC use have little effect on hemoglobin levels.
**Solid Organ Transplantation**							
**Solid organ transplantation** This condition is associated with increased risk for adverse health events as a result of pregnancy (Box 3).							
a. No graft failure	2		2/3		2		**Clarification (DMPA):** DMPA use among persons receiving long-term immunosuppressive therapy with a history of, or risk factors for, nontraumatic fractures is classified as category 3. Otherwise, DMPA use for persons with solid organ transplantation is classified as category 2. **Evidence:** One study observed no differences in transplant-related adverse outcomes (e.g., infection, graft failure, and graft rejection) or occurrence of pregnancy between transplant recipients using the implant and those using no hormonal method (Supplementary Appendix, https://stacks.cdc.gov/view/cdc/156516). No direct evidence was identified on bone health or fracture with use of POCs, including DMPA, among persons with solid organ transplantation. Persons with solid organ transplantation have a higher prevalence of osteoporosis and fracture than the general population, especially in the early posttransplantation period (*152*). Use of DMPA, which has been associated with small changes in bone mineral density compared with nonuse (*4*) might further elevate risk for fracture among persons with solid organ transplantation.
b. Graft failure	2		2/3		2		
**Drug Interactions**							
**Antiretrovirals used for prevention (PrEP) or treatment of HIV infection**							**Comment:** These recommendations generally are for ARV agents used alone. However, most persons receiving ARV are using multiple drugs in combination. In general, whether interactions between ARVs and hormonal contraceptives differ when ARVs are given alone or in combination is unknown.
See the following guidelines for the most up-to-date recommendations on drug-drug interactions between hormonal contraception and antiretrovirals: 1) Recommendations for the Use of Antiretroviral Drugs During Pregnancy and Interventions to Reduce Perinatal HIV Transmission in the United States (https://clinicalinfo.hiv.gov/en/guidelines/perinatal/prepregnancy-counseling-childbearing-age-overview?view=full#table-3) (*153*) and 2) Guidelines for the Use of Antiretroviral Agents in Adults and Adolescents with HIV (https://clinicalinfo.hiv.gov/en/guidelines/hiv-clinical-guidelines-adult-and-adolescent-arv/drug-interactions-overview?view=full) (*154*).							
a. Nucleoside reverse transcriptase inhibitors (NRTIs)							
i. Abacavir (ABC)	1		1		1		**Evidence:** NRTIs do not appear to have significant risk for interactions with hormonal contraceptive methods (*155**–**160*).
ii. Tenofovir (TDF)	1		1		1		
iii. Zidovudine (AZT)	1		1		1		
iv. Lamivudine (3TC)	1		1		1		
v. Didanosine (DDI)	1		1		1		
vi. Emtricitabine (FTC)	1		1		1		
vii. Stavudine (D4T)	1		1		1		
b. Nonnucleoside reverse transcriptase inhibitors (NNRTIs)							
i. Efavirenz (EFV)	2		1		2		**Clarification:** Evidence suggests drug interactions between EFV and certain hormonal contraceptives. These interactions might reduce the effectiveness of the hormonal contraceptive. **Evidence:** One study found that women using etonogestrel implants with EFV had a higher pregnancy rate than women not using ARVs, although confidence intervals overlapped and absolute pregnancy rates were still lower than for other hormonal methods; another study found that etonogestrel levels were decreased and 5% of women had presumptive ovulation while using etonogestrel implants with EFV (*161**,**162*). Three studies of women using LNG implants demonstrated increased pregnancy rates for women using EFV-containing ARV therapy compared with no ARV use, although absolute pregnancy rates were still lower than for other hormonal methods in one study (*162**–**164*); another study of LNG implant users found no difference in pregnancy rates with EFV compared with no EFV (165). No significant effects were found on pregnancy rates, DMPA levels, EFV levels, or HIV disease progression in women using DMPA and EFV compared with DMPA alone (*162**,**165**–**169*). No significant effects were found on HIV disease progression in women using LNG implants and EFV compared with no ARVs (*164*). No data have assessed effectiveness of contraceptive implants during later years of use when progestin concentrations are lower and risk for failure from drug interactions might be greater.
ii. Etravirine (ETR)	1		1		1		—
iii. Nevirapine (NVP)	1		1		1		**Evidence:** Five studies found no significant increase in pregnancy rates among women using implants and NVP compared with implants alone (*162**–**165**,**170*). Four studies found no significant increase in pregnancy rates among women using DMPA or other contraceptive injectables and NVP compared with DMPA or other contraceptive injectables alone (*162**,**165**,**168**,**171*). One study found no ovulations or changes in DMPA concentrations (*166*). No effect was found on HIV disease progression with use of NVP and DMPA or LNG implants (*164**,**166**,**168**–**170**,**172*). No data have assessed effectiveness of contraceptive implants during later years of use when progestin concentrations are lower and risk for failure from drug interactions might be greater.
iv. Rilpivirine (RPV)	1		1		1		—
c. Ritonavir-boosted protease inhibitors							
i. Ritonavir-boosted atazanavir (ATV/r)	2		1		2		**Clarification:** Theoretically, drug interactions might occur between certain ritonavir-boosted protease inhibitors and certain hormonal contraceptives that might reduce the effectiveness of the hormonal contraceptive. Any potential effect on contraceptive effectiveness is likely to be lower with DMPA than with other POCs because of the higher dose of DMPA. **Evidence:** One pharmacokinetic study demonstrated increased progestin concentrations with use of POPs and ATV/r compared with POPs alone (*173*).
ii. Ritonavir-boosted darunavir (DRV/r)	2		1		2		**Clarification:** Theoretically, drug interactions might occur between certain ritonavir-boosted protease inhibitors and certain hormonal contraceptives that might reduce the effectiveness of the hormonal contraceptive. Any potential effect on contraceptive effectiveness is likely to be lower with DMPA than with other POCs because of the higher dose of DMPA.
iii. Ritonavir-boosted fosamprenavir (FPV/r)	2		1		2		**Clarification:** Theoretically, drug interactions might occur between certain ritonavir-boosted protease inhibitors and certain hormonal contraceptives that might reduce the effectiveness of the hormonal contraceptive. Any potential effect on contraceptive effectiveness is likely to be lower with DMPA than with other POCs because of the higher dose of DMPA.
iv. Ritonavir-boosted lopinavir (LPV/r)	1		1		1		**Evidence:** One study demonstrated no pregnancies, no ovulations, no change in LPV/r level, and no change in HIV disease progression in women using DMPA (*174*); another study found a small increase in pregnancy rate in women using DMPA with LPV/r compared with no ARV therapy, however confidence intervals overlapped (*162*). Two studies found no increased risk for pregnancy in women using implants (*162**,**163*). Two studies found contraceptive hormones increased in women using LPV/r with DMPA or etonogestrel implants (*161**,**174*).
v. Ritonavir-boosted saquinavir (SQV/r)	2		1		2		**Clarification:** Theoretically, drug interactions might occur between certain ritonavir-boosted protease inhibitors and certain hormonal contraceptives that might reduce the effectiveness of the hormonal contraceptive. Any potential effect on contraceptive effectiveness is likely to be lower with DMPA than with other POCs because of the higher dose of DMPA.
vi. Ritonavir-boosted tipranavir (TPV/r)	2		1		2		**Clarification:** Theoretically, drug interactions might occur between certain ritonavir-boosted protease inhibitors and certain hormonal contraceptives that might reduce the effectiveness of the hormonal contraceptive. Any potential effect on contraceptive effectiveness is likely to be lower with DMPA than with other POCs because of the higher dose of DMPA.
d. Protease inhibitors without ritonavir							
i. Atazanavir (ATV)	1		1		1		**Comment:** When ATV is administered with cobicistat, theoretical concern exists for a drug interaction with hormonal contraceptives. Cobicistat is an inhibitor of CYP3A and CYP2D6 and could theoretically increase contraceptive hormone levels. However, its effects on CYP enzymes and drug levels might vary when combined with other ARVs.
ii. Fosamprenavir (FPV)	2		2		2		**Clarification:** Theoretical concern exists that interactions between FPV and hormonal contraceptives leading to decreased levels of FPV might diminish effectiveness of the ARV drug. The drug interaction likely involves CYP3A4 pathways; POCs have less effect on CYP3A4 enzymes than CHCs.
iii. Indinavir (IDV)	1		1		1		—
iv. Nelfinavir (NFV)	2		1		2		**Clarification:** Theoretically, drug interactions might occur between certain protease inhibitors and certain hormonal contraceptives that might reduce the effectiveness of the hormonal contraceptive. Any potential effect on contraceptive effectiveness is likely to be lower with DMPA than with other POCs because of the higher dose of DMPA. Concern exists that interactions between NFV and POCs might decrease NFV levels. **Evidence:** One study found no pregnancies, no ovulations, no change in DMPA concentrations and no change in HIV disease progression with use of DMPA and NFV compared with DMPA alone; NFV concentrations were decreased with concomitant DMPA use (*166**,**168*).
e. CCR5 co-receptor antagonists							
i. Maraviroc (MVC)	1		1		1		—
f. HIV integrase strand transfer inhibitors							
i. Raltegravir (RAL)	1		1		1		—
ii. Dolutegravir (DTG)	1		1		1		—
iii. Elvitegravir (EVG)	1		1		1		**Comment:** When EVG is administered with cobicistat, theoretical concern exists for a drug interaction with hormonal contraceptives. Cobicistat is an inhibitor of CYP3A and CYP2D6 and could theoretically increase contraceptive hormone levels. However, its effects on CYP enzymes and drug levels might vary when combined with other ARVs.
g. Fusion inhibitors							
i. Enfuvirtide	1		1		1		—
**Anticonvulsant therapy**							
a. Certain anticonvulsants (phenytoin, carbamazepine, barbiturates, primidone, topiramate, and oxcarbazepine)	2		1		3		**Clarification:** Although the interaction of certain anticonvulsants with POPs and etonogestrel implants is not harmful, it is likely to reduce the effectiveness of POPs and etonogestrel implants. Whether increasing the hormone dose of POPs alleviates this concern remains unclear. Use of other contraceptives should be encouraged for persons who are long-term users of any of these drugs. Use of DMPA is a category 1 because its effectiveness is not decreased by use of certain anticonvulsants. **Evidence:** Use of certain anticonvulsants might decrease the effectiveness of POCs (*175**–**178*).
b. Lamotrigine	1		1		1		**Evidence:** No drug interactions have been reported among women with epilepsy receiving lamotrigine and POCs (*178**,**179*).
**Antimicrobial therapy**							
a. Broad-spectrum antibiotics	1		1		1		—
b. Antifungals	1		1		1		—
c. Antiparasitics	1		1		1		—
d. Rifampin or rifabutin therapy	2		1		3		**Clarification:** Although the interaction of rifampin or rifabutin with POPs and etonogestrel implants is not harmful, it is likely to reduce the effectiveness of POPs and etonogestrel implants. Use of other contraceptives should be encouraged for persons who are long-term users of any of these drugs. Use of DMPA is a category 1 because its effectiveness is not decreased by use of rifampin or rifabutin. Whether increasing the hormone dose of POPs alleviates this concern remains unclear.
**Psychotropic medications**							**Comment:** For many common psychotropic agents, limited or no theoretical concern exits for clinically significant drug interactions when co-administered with hormonal contraceptives. However, either no or very limited data exist examining potential interactions for these classes of medications.
a. Selective serotonin reuptake inhibitors (SSRIs)	1		1		1		**Evidence:** No evidence specifically examined the use of POCs with SSRIs. Limited clinical and pharmacokinetic data do not demonstrate concern for SSRIs decreasing the effectiveness of oral contraceptives. Limited evidence suggests that for women taking SSRIs, the use of hormonal contraceptives was not associated with differences in effectiveness of the SSRI for treatment or in adverse events when compared with women not taking hormonal contraceptives (*180*). **Comment:** Drugs that are inhibitors of CYP3A4 or CYP2C9 theoretically have the potential to increase levels of contraceptive steroid, which might increase adverse events. Fluvoxamine is an SSRI known to be a moderate inhibitor of both 3A4 and 2C9; however, no clinical or pharmacokinetic studies were identified to explore potential drug-drug interactions.
**St. John’s wort**	2		1		2		**Evidence:** No evidence specifically examined the use of POCs with St. John’s wort. Although clinical data are limited, studies with pharmacokinetic and pharmacodynamics outcomes raise concern that St. John’s wort might decrease effectiveness of hormonal contraceptives, including increased risk for breakthrough bleeding and ovulation and increased metabolism of estrogen and progestin. Any interactions might be dependent on the dose of St. John’s wort, and the concentration of active ingredients across types of St. John’s wort preparations might vary (*181*). **Comment:** Any potential effect on contraceptive effectiveness is likely to be lower with DMPA than with other POCs because of the higher dose of DMPA.

**Abbreviations:** ARV = antiretroviral; BMD = bone mineral density; BMI = body mass index; CHC = combined hormonal contraceptive; CKD = chronic kidney disease; COC = combined oral contraceptive; Cu-IUD = copper intrauterine device; CYP = cytochrome P450; DMPA = depot medroxyprogesterone acetate; DRSP = drospirenone; DVT = deep venous thrombosis; hCG = human chorionic gonadotropin; HDL = high-density lipoprotein; IBD = inflammatory bowel disease; IM = intramuscular; LDL = low-density lipoprotein; LNG = levonorgestrel; LNG-IUD = levonorgestrel intrauterine device; NA = not applicable; PE = pulmonary embolism; PID = pelvic inflammatory disease; POC = progestin-only contraceptive; POP = progestin-only pill; PrEP = pre-exposure prophylaxis; RCT = randomized clinical trial; SLE = systemic lupus erythematosus; STI = sexually transmitted infection; U.S. MEC = *U.S. Medical Eligibility Criteria for Contraceptive Use*; U.S. SPR = *U.S. Selected Practice Recommendations for Contraceptive Use*; VTE = venous thromboembolism.

## Appendix D Classifications for Combined Hormonal Contraceptives

Combined hormonal contraceptives (CHCs) include combined oral contraceptives (COCs) (containing a progestin plus ethinyl estradiol [EE] ≤35 *μ*g, estradiol valerate, or estetrol); combined transdermal patches (levonorgestrel/EE or norelgestromin/EE); and combined vaginal rings (etonogestrel/EE or segesterone acetate/EE) (Box D1) (Table D1). Limited information is available about the safety of COCs with estradiol valerate or estetrol, combined transdermal patches, and combined vaginal rings among users with specific medical conditions. Evidence indicates that estradiol valerate and estetrol COCs, combined transdermal patches, and combined vaginal rings provide comparable safety and pharmacokinetic profiles to EE-containing COCs with similar hormone formulations (*1**–**33*). Pending further studies, the evidence available for recommendations about EE-containing COCs applies to the recommendations for estradiol valerate and estetrol COCs, the combined transdermal patch, and vaginal rings. Therefore, the estradiol valerate and estetrol COCs, the patches, and the rings should have the same categories as EE-containing COCs, except where noted. The assigned categories should be considered a preliminary best judgment, which will be reevaluated as new data become available.

COCs, patches, and rings do not protect against sexually transmitted infections (STIs), including HIV infection, and patients using CHCs should be counseled that consistent and correct use of external (male) latex condoms reduces the risk for STIs, including HIV infection (*34*). Use of internal (female) condoms can provide protection from transmission of STIs, although data are limited (*34*). Patients also should be counseled that pre-exposure prophylaxis, when taken as prescribed, is highly effective for preventing HIV infection (*35*).

BOX D1 Categories for classifying combined hormonal contraceptives U.S. MEC 1 = A condition for which there is no restriction for the use of the contraceptive method U.S. MEC 2 = A condition for which the advantages of using the method generally outweigh the theoretical or proven risks U.S. MEC 3 = A condition for which the theoretical or proven risks usually outweigh the advantages of using the method U.S. MEC 4 = A condition that represents an unacceptable health risk if the contraceptive method is used **Abbreviation:** U.S. MEC *= U.S. Medical Eligibility Criteria for Contraceptive Use*.

**TABLE D1:** Classifications for combined hormonal contraceptives, including pill, patch, and ring

Condition	CHC		Clarification/Evidence/Comment
**Personal Characteristics and Reproductive History**			
**Pregnancy**	NA		**Clarification:** Use of CHCs is not required. No known harm to the patient, the course of pregnancy, or the fetus occurs if CHCs are inadvertently used during pregnancy.
**Age**			**Evidence:** Evidence is inconsistent about whether CHC use affects fracture risk (*36**–**47*), although three recent studies demonstrate no effect (*36**,**37**,**47*). CHC use might decrease BMD in adolescents, especially in those choosing very low-dose formulations (COCs containing <30 *µg* ethinyl estradiol) (*48**–**61*). CHC use has little to no effect on BMD in premenopausal women (*62**–**76*) and might preserve bone mass in those who are perimenopausal (*77**–**85*). BMD is a surrogate marker for fracture risk that might not be valid for premenopausal women and therefore might not accurately predict current or future (postmenopausal) fracture risk (*86**–**88*). **Comment:** The risk for cardiovascular disease increases with age and might increase with CHC use. In the absence of other adverse clinical conditions, CHCs can be used until menopause.
a. Menarche to <40 years	1		
b. ≥40 years	2		
**Parity**			
a. Nulliparous	1		—
b. Parous	1		—
**Breastfeeding**			
a. <21 days postpartum	4		**Clarification (breastfeeding):** Breastfeeding provides important health benefits for breastfeeding parent and infant. The U.S. Dietary Guidelines for Americans and American Academy of Pediatrics recommend that infants be exclusively breastfed for about the first 6 months with continued breastfeeding while introducing appropriate complementary foods for 1 year or longer (*89*) or up to age 2 years or longer (*90*). **Evidence (breastfeeding):** Clinical studies demonstrate conflicting results regarding effects on breastfeeding continuation or exclusivity in women exposed to COCs during lactation. No consistent effects on infant growth or illness have been reported. Adverse health outcomes or manifestations of exogenous estrogen in infants exposed to CHCs through breast milk have not been demonstrated; however, studies have been inadequately designed to determine whether a risk for either serious or subtle long-term effects exists (*91*). **Evidence:** One study examined use of CHCs during the postpartum period and found that VTE rates were higher for CHC users compared with nonusers at all time points postpartum (*92*). Rates were significantly different only after 13 weeks postpartum; however, the numbers needed to harm were lowest in the first 6 weeks postpartum. VTE risk is increased during pregnancy and the postpartum period; this risk is most pronounced in the first 3 weeks after delivery, decreasing to near baseline levels by 42 days postpartum (*93**–**97*). **Comment:** Risk factors for breastfeeding difficulties include previous breastfeeding difficulties, certain medical conditions, certain perinatal complications, and preterm birth. For all breastfeeding persons, with or without risk factors for breastfeeding difficulties, discussions about contraception should include information about risks, benefits, and alternatives.
b. 21 to <30 days postpartum			
i. With other risk factors for VTE (e.g., age ≥35 years, previous VTE, thrombophilia, immobility, transfusion at delivery, peripartum cardiomyopathy, BMI ≥30 kg/m^2^, postpartum hemorrhage, postcesarean delivery, preeclampsia, or smoking)	3		**Clarification:** For persons with other risk factors for VTE, these risk factors might increase the classification to a category 4. **Clarification (breastfeeding):** Breastfeeding provides important health benefits for breastfeeding parent and infant. The U.S. Dietary Guidelines for Americans and American Academy of Pediatrics recommend that infants be exclusively breastfed for about the first 6 months with continued breastfeeding while introducing appropriate complementary foods for 1 year or longer (*89*) or up to age 2 years or longer (*90*). **Evidence (breastfeeding):** Clinical studies demonstrate conflicting results regarding effects on breastfeeding continuation or exclusivity in women exposed to COCs during lactation. No consistent effects on infant growth or illness have been reported. Adverse health outcomes or manifestations of exogenous estrogen in infants exposed to CHCs through breast milk have not been demonstrated; however, studies have been inadequately designed to determine whether a risk for either serious or subtle long-term effects exists (*91*). **Evidence:** One study examined use of CHCs during the postpartum period and found that VTE rates were higher for CHC users compared with nonusers at all time points postpartum (*92*). Rates were significantly different only after 13 weeks postpartum; however, the numbers needed to harm were lowest in the first 6 weeks postpartum. VTE risk is increased during pregnancy and the postpartum period; this risk is most pronounced in the first 3 weeks after delivery, decreasing to near baseline levels by 42 days postpartum (*93**–**97*). **Comment:** Risk factors for breastfeeding difficulties include previous breastfeeding difficulties, certain medical conditions, certain perinatal complications, and preterm birth. For all breastfeeding persons, with or without risk factors for breastfeeding difficulties, discussions about contraception should include information about risks, benefits, and alternatives.
ii. Without other risk factors for VTE	3		**Clarification (breastfeeding):** Breastfeeding provides important health benefits for breastfeeding parent and infant. The U.S. Dietary Guidelines for Americans and American Academy of Pediatrics recommend that infants be exclusively breastfed for about the first 6 months with continued breastfeeding while introducing appropriate complementary foods for 1 year or longer (*89*) or up to age 2 years or longer (*90*). **Evidence (breastfeeding):** Clinical studies demonstrate conflicting results regarding effects on breastfeeding continuation or exclusivity in women exposed to COCs during lactation. No consistent effects on infant growth or illness have been reported. Adverse health outcomes or manifestations of exogenous estrogen in infants exposed to CHCs through breast milk have not been demonstrated; however, studies have been inadequately designed to determine whether a risk for either serious or subtle long-term effects exists (*91*). **Evidence:** One study examined use of CHCs during the postpartum period and found that VTE rates were higher for CHC users compared with nonusers at all time points postpartum (*92*). Rates were significantly different only after 13 weeks postpartum; however, the numbers needed to harm were lowest in the first 6 weeks postpartum. VTE risk is increased during pregnancy and the postpartum period; this risk is most pronounced in the first 3 weeks after delivery, decreasing to near baseline levels by 42 days postpartum (*93**–**97*). **Comment:** Risk factors for breastfeeding difficulties include previous breastfeeding difficulties, certain medical conditions, certain perinatal complications, and preterm birth. For all breastfeeding persons, with or without breastfeeding difficulties, discussions about contraception should include information about risks, benefits, and alternatives.
c. 30–42 days postpartum			
i. With other risk factors for VTE (e.g., age ≥35 years, previous VTE, thrombophilia, immobility, transfusion at delivery, peripartum cardiomyopathy, BMI ≥30 kg/m^2^, postpartum hemorrhage, postcesarean delivery, preeclampsia, or smoking)	3		**Clarification:** For persons with other risk factors for VTE, these risk factors might increase the classification to a category 4. **Clarification (breastfeeding):** Breastfeeding provides important health benefits for breastfeeding parent and infant. The U.S. Dietary Guidelines for Americans and American Academy of Pediatrics recommend that infants be exclusively breastfed for about the first 6 months with continued breastfeeding while introducing appropriate complementary foods for 1 year or longer (*89*) or up to age 2 years or longer (*90*). **Evidence (breastfeeding):** Clinical studies demonstrate conflicting results regarding effects on breastfeeding continuation or exclusivity in women exposed to COCs during lactation. No consistent effects on infant growth or illness have been reported. Adverse health outcomes or manifestations of exogenous estrogen in infants exposed to CHCs through breast milk have not been demonstrated; however, studies have been inadequately designed to determine whether a risk for either serious or subtle long-term effects exists (*91*). **Evidence:** One study examined use of CHCs during the postpartum period and found that VTE rates were higher for CHC users compared with nonusers at all time points postpartum (*92*). Rates were significantly different only after 13 weeks postpartum; however, the numbers needed to harm were lowest in the first 6 weeks postpartum. VTE risk is increased during pregnancy and the postpartum period; this risk is most pronounced in the first 3 weeks after delivery, decreasing to near baseline levels by 42 days postpartum (*93**–**97*). **Comment:** Risk factors for breastfeeding difficulties include previous breastfeeding difficulties, certain medical conditions, certain perinatal complications, and preterm birth. For all breastfeeding persons, with or without breastfeeding difficulties, discussions about contraception should include information about risks, benefits, and alternatives.
ii. Without other risk factors for VTE	2		**Clarification (breastfeeding):** Breastfeeding provides important health benefits for breastfeeding parent and infant. The U.S. Dietary Guidelines for Americans and American Academy of Pediatrics recommend that infants be exclusively breastfed for about the first 6 months with continued breastfeeding while introducing appropriate complementary foods for 1 year or longer (*89*) or up to age 2 years or longer (*90*). **Evidence (breastfeeding):** Clinical studies demonstrate conflicting results regarding effects on breastfeeding continuation or exclusivity in women exposed to COCs during lactation. No consistent effects on infant growth or illness have been reported. Adverse health outcomes or manifestations of exogenous estrogen in infants exposed to CHCs through breast milk have not been demonstrated; however, studies have been inadequately designed to determine whether a risk for either serious or subtle long-term effects exists (*91*). **Evidence:** One study examined use of CHCs during the postpartum period and found that VTE rates were higher for CHC users compared with nonusers at all time points postpartum (*92*). Rates were significantly different only after 13 weeks postpartum; however, the numbers needed to harm were lowest in the first 6 weeks postpartum. VTE risk is increased during pregnancy and the postpartum period; this risk is most pronounced in the first 3 weeks after delivery, decreasing to near baseline levels by 42 days postpartum (*93**–**97*). **Comment:** Risk factors for breastfeeding difficulties include previous breastfeeding difficulties, certain medical conditions, certain perinatal complications, and preterm birth. For all breastfeeding persons, with or without breastfeeding difficulties, discussions about contraception should include information about risks, benefits, and alternatives.
d. >42 days postpartum	2		**Clarification (breastfeeding):** Breastfeeding provides important health benefits for breastfeeding parent and infant. The U.S. Dietary Guidelines for Americans and American Academy of Pediatrics recommend that infants be exclusively breastfed for about the first 6 months with continued breastfeeding while introducing appropriate complementary foods for 1 year or longer (*89*) or up to age 2 years or longer (*90*). **Evidence:** Clinical studies demonstrate conflicting results regarding effects on breastfeeding continuation or exclusivity in women exposed to COCs during lactation. No consistent effects on infant growth or illness have been reported. Adverse health outcomes or manifestations of exogenous estrogen in infants exposed to CHCs through breast milk have not been demonstrated; however, studies have been inadequately designed to determine whether a risk for either serious or subtle long-term effects exists (*91*). **Comment:** Risk factors for breastfeeding difficulties include previous breastfeeding difficulties, certain medical conditions, certain perinatal complications, and preterm birth. For all breastfeeding persons, with or without breastfeeding difficulties, discussions about contraception should include information about risks, benefits, and alternatives.
**Postpartum** (nonbreastfeeding)			
a. <21 days postpartum	4		**Evidence:** One study examined use of CHCs during the postpartum period and found that VTE rates were higher for CHC users compared with nonusers at all time points postpartum (*92*). Rates were significantly different only after 13 weeks postpartum; however, the numbers needed to harm were lowest in the first 6 weeks postpartum. VTE risk is increased during pregnancy and the postpartum period; this risk is most pronounced in the first 3 weeks after delivery, decreasing to near baseline levels by 42 days postpartum (*93**–**97*). Risk for pregnancy during the first 21 days postpartum is very low but increases after that point; ovulation before first menses is common (*98*).
b. 21–42 days postpartum			
i. With other risk factors for VTE (e.g., age ≥35 years, previous VTE, thrombophilia, immobility, transfusion at delivery, peripartum cardiomyopathy, BMI ≥30 kg/m^2^ postpartum hemorrhage, postcesarean delivery, preeclampsia, or smoking)	3		**Clarification:** For persons with other risk factors for VTE, these risk factors might increase the classification to a category 4. **Evidence:** One study examined use of CHCs during the postpartum period and found that VTE rates were higher for CHC users compared with nonusers at all time points postpartum (*92*). Rates were significantly different only after 13 weeks postpartum; however, the numbers needed to harm were lowest in the first 6 weeks postpartum. VTE risk is increased during pregnancy and the postpartum period; this risk is most pronounced in the first 3 weeks after delivery, decreasing to near baseline levels by 42 days postpartum (*93**–**97*).
ii. Without other risk factors for VTE	2		**Evidence:** One study examined use of CHCs during the postpartum period and found that VTE rates were higher for CHC users compared with nonusers at all time points postpartum (*92*). Rates were significantly different only after 13 weeks postpartum; however, the numbers needed to harm were lowest in the first 6 weeks postpartum. VTE risk is increased during pregnancy and the postpartum period; this risk is most pronounced in the first 3 weeks after delivery, decreasing to near baseline levels by 42 days postpartum (*93**–**97*).
c. >42 days postpartum	1		—
**Postabortion **(spontaneous or induced)			
a. First trimester abortion			**Clarification:** CHCs may be started immediately after abortion completion or at time of medication abortion initiation. **Evidence:** Evidence suggests that there is no increased risk for adverse events when CHCs are initiated after first trimester procedural or medication abortion (immediately or delayed) (*99*). Immediate initiation of COCs after first trimester procedural or medication abortion did not cause clinically significant changes in coagulation parameters compared with placebo, a hormonal IUD, a nonhormonal contraceptive method, or delayed COC initiation (*100*).
i. Procedural (surgical)	1		
ii. Medication	1		
iii. Spontaneous abortion with no intervention	1		
b. Second trimester abortion			**Clarification:** CHCs may be started immediately after abortion completion or at time of medication abortion initiation. **Evidence:** Limited evidence suggests that there is no increased risk for adverse events when CHCs are initiated after second trimester procedural abortion (immediately or delayed) (*99*).
i. Procedural (surgical)	1		
ii. Medication	1		
iii. Spontaneous abortion with no intervention	1		
c. Immediate postseptic abortion	1		**Clarification:** CHCs may be started immediately after abortion completion or at time of medication abortion initiation.
**Past ectopic pregnancy**	1		**Comment:** The risk for future ectopic pregnancy is increased among those who have had an ectopic pregnancy in the past. CHCs protect against pregnancy in general, including ectopic gestation.
**History of pelvic surgery**	1		—
**Smoking**			**Evidence:** COC users who smoked were at increased risk for cardiovascular diseases, especially myocardial infarction, compared with those who did not smoke. Studies also demonstrated an increased risk for myocardial infarction with increasing number of cigarettes smoked per day (*101**–**113*).
a. Age <35 years	2		
b. Age ≥35 years			
i. <15 cigarettes per day	3		
ii. ≥15 cigarettes per day	4		
**Obesity**			**Clarification:** Risk for thrombosis increases with multiple risk factors, such as obesity, older age (e.g., ≥40 years), diabetes, smoking, family history of thrombosis, and dyslipidemia. When a person has multiple risk factors, any of which alone would increase risk for thrombosis, use of CHCs might increase thrombosis risk to an unacceptable level. However, a simple addition of categories for multiple risk factors is not intended; for example, a combination of two category 2 risk factors might not necessarily warrant a higher category. **Evidence:** Although the absolute risk for VTE in healthy women of reproductive age is small, COC use and higher BMI independently increase risk for VTE, with the greatest relative risks among those with both risk factors. From a systematic review, COC users with obesity consistently had a relative risk for VTE of 5–8 times that of nonusers with obesity (Supplementary Appendix, https://stacks.cdc.gov/view/cdc/156516). Research examining the interaction between COCs and BMI on VTE risk is limited, particularly for those in the highest BMI categories (BMI ≥35 kg/m^2^). Comparative studies on the risk for VTE among contraceptive patch or ring users by weight or BMI were not identified (*114**–**116*). Limited evidence suggests that COC users with obesity do not have a higher risk for acute myocardial infarction or stroke than do nonusers with obesity (*114*) (Supplementary Appendix, https://stacks.cdc.gov/view/cdc/156516). Limited evidence suggests that effectiveness of certain COC formulations might decrease with increasing BMI; however the observed reductions in effectiveness are minimal and evidence is conflicting (*117**–**124*). Effectiveness of the patch might be reduced in women with BMI ≥30 kg/m^2^ or weight >90 kg (*125*).
a. BMI ≥30 kg/m^2^	2		
b. Menarche to <18 years and BMI ≥30 kg/m^2^	2		
**History of bariatric surgery** This condition is associated with increased risk for adverse health events as a result of pregnancy (Box 3).			
a. Restrictive procedures: decrease storage capacity of the stomach (vertical banded gastroplasty, laparoscopic adjustable gastric band, or laparoscopic sleeve gastrectomy)	1		**Evidence:** Limited evidence demonstrated no substantial decrease in effectiveness of oral contraceptives among women who underwent laparoscopic placement of an adjustable gastric band (*126*).
b. Malabsorptive procedures: decrease absorption of nutrients and calories by shortening the functional length of the small intestine (Roux-en-Y gastric bypass or biliopancreatic diversion)	COCs: 3 Patch and ring: 1		**Evidence:** Limited evidence demonstrated no substantial decrease in effectiveness of oral contraceptives among women who underwent a biliopancreatic diversion; however, evidence from pharmacokinetic studies reported conflicting results of oral contraceptive effectiveness among women who underwent a jejunoileal bypass (*126*). **Comment:** Bariatric surgical procedures involving a malabsorptive component have the potential to decrease oral contraceptive effectiveness, perhaps further decreased by postoperative complications, such as long-term diarrhea or vomiting.
**Surgery**			
a. Minor surgery without immobilization	1		—
b. Major surgery			
ii. Without prolonged immobilization	2		—
i. With prolonged immobilization	4		—
**Cardiovascular Disease**			
**Multiple risk factors for atherosclerotic cardiovascular disease** (e.g., older age, smoking, diabetes, hypertension, low HDL, high LDL, or high triglyceride levels)	3/4		**Clarification:** When a person has multiple major risk factors, any of which alone would substantially increase risk for cardiovascular disease, use of CHCs might increase risk to an unacceptable level. However, a simple addition of categories for multiple risk factors is not intended; for example, a combination of two category 2 risk factors might not necessarily warrant a higher category. **Clarification:** The recommendations apply to known pre-existing medical conditions or characteristics. Few if any screening tests are needed before initiation of contraception. See U.S. *S*PR (https://www.cdc.gov/contraception/hcp/usspr/HYPERLINK) (*127*).
**Hypertension** Systolic blood pressure ≥160 mm Hg or diastolic blood pressure ≥100 mm Hg are associated with increased risk for adverse health events as a result of pregnancy (Box 3).			
a. Adequately controlled hypertension	3		**Clarification:** For all categories of hypertension, classifications are based on the assumption that no other risk factors exist for cardiovascular disease. When multiple risk factors do exist, risk for cardiovascular disease might increase substantially. A single reading of blood pressure level is not sufficient to classify a person as hypertensive. **Clarification:** Persons adequately treated for hypertension are at reduced risk for acute myocardial infarction and stroke compared with untreated persons. Although no data exist, CHC users with adequately controlled and monitored hypertension should be at reduced risk for acute myocardial infarction and stroke compared with untreated hypertensive CHC users. **Evidence:** Among women with hypertension, COC users were at higher risk than nonusers for stroke, acute myocardial infarction, and peripheral arterial disease (*101**,**103**,**110**–**113**,**128**–**142*). Discontinuation of COCs in women with hypertension might improve blood pressure control (*143*).
b. Elevated blood pressure levels (properly taken measurements)			**Clarification:** For all categories of hypertension, classifications are based on the assumption that no other risk factors exist for cardiovascular disease. When multiple risk factors do exist, risk for cardiovascular disease might increase substantially. A single reading of blood pressure level is not sufficient to classify a person as hypertensive. **Evidence:** Among women with hypertension, COC users were at higher risk than nonusers for stroke, acute myocardial infarction, and peripheral arterial disease (*101**,**103**,**110**–**113**,**128**–**142*). Discontinuation of COCs in women with hypertension might improve blood pressure control (*143*).
i. Systolic 140–159 mm Hg or diastolic 90–99 mm Hg	3		
ii. Systolic ≥160 mm Hg or diastolic ≥100 mm Hg	4		
c. Vascular disease	4		
**History of high blood pressure during pregnancy** (when current blood pressure is measurable and normal)	2		**Evidence:** Women with a history of high blood pressure in pregnancy who also used COCs had a higher risk for myocardial infarction and VTE than did COC users who did not have a history of high blood pressure during pregnancy. The absolute risks for acute myocardial infarction and VTE in this population remained small (*112**,**129**,**141**,**142**,**144**–**150*).
**Deep venous thrombosis/ Pulmonary embolism** This condition is associated with increased risk for adverse health events as a result of pregnancy (Box 3).			
a. Current or history of DVT/PE, receiving anticoagulant therapy (therapeutic dose) (e.g., acute DVT/PE or long-term therapeutic dose)	3		**Clarification**: Persons using anticoagulant therapy are at risk for gynecologic complications of therapy, such as heavy or prolonged bleeding and hemorrhagic ovarian cysts. CHCs can be of benefit in preventing or treating these complications. When a contraceptive method is used as a therapy, rather than solely to prevent pregnancy, the risk/benefit ratio might differ and should be considered on a case-by-case basis. **Clarification:** When a patient discontinues therapeutic dose of anticoagulant therapy, careful consideration should be given to transitioning from CHCs to a progestin-only or nonhormonal method, if acceptable to the patient. **Evidence**: Limited evidence was identified on use of CHCs among women with DVT/PE receiving anticoagulant therapy (Supplementary Appendix, https://stacks.cdc.gov/view/cdc/156516). In one study among women with a history of acute VTE currently receiving therapeutic anticoagulant therapy (i.e., rivaroxaban or enoxaparin/vitamin K antagonist [warfarin or acenocoumarol]), the incidence of recurrent VTE was similar among estrogen users (CHC or estrogen-only pills), POC users, and women not on hormonal therapy (*151*).
b. History of DVT/PE, receiving anticoagulant therapy (prophylactic dose)			**Clarification**: Persons using anticoagulant therapy are at risk for gynecologic complications of therapy, such as heavy or prolonged bleeding and hemorrhagic ovarian cysts. CHCs can be of benefit in preventing or treating these complications. When a contraceptive method is used as a therapy, rather than solely to prevent pregnancy, the risk/benefit ratio might differ and should be considered on a case-by-case basis.
i. Higher risk for recurrent DVT/PE (one or more risk factors)	4		
• Thrombophilia (e.g., factor V Leiden mutation; prothrombin gene mutation; protein S, protein C, and antithrombin deficiencies; or antiphospholipid syndrome) • Active cancer (metastatic, receiving therapy, or within 6 months after clinical remission), excluding nonmelanoma skin cancer • History of recurrent DVT/PE			
ii. Lower risk for recurrent DVT/PE (no risk factors)	3		
c. History of DVT/PE, not receiving anticoagulant therapy			
i. Higher risk for recurrent DVT/PE (one or more risk factors)	4		—
• History of estrogen-associated DVT/PE • Pregnancy-associated DVT/PE • Idiopathic DVT/PE • Thrombophilia (e.g., factor V Leiden mutation; prothrombin gene mutation; protein S, protein C, and antithrombin deficiencies; or antiphospholipid syndrome) • Active cancer (metastatic, receiving therapy, or within 6 months after clinical remission), excluding nonmelanoma skin cancer • History of recurrent DVT/PE			
ii. Lower risk for recurrent DVT/PE (no risk factors)	3		—
d. Family history (first-degree relatives)	2		**Comment:** Certain conditions that increase the risk for DVT/PE are heritable.
**Thrombophilia** (e.g., factor V Leiden mutation; prothrombin gene mutation; protein S, protein C, and antithrombin deficiencies; or antiphospholipid syndrome) This condition is associated with increased risk for adverse health events as a result of pregnancy (Box 3).	4		**Clarification**: Routine screening in the general population before contraceptive initiation is not recommended. **Clarification**: If a person has current or history of DVT/PE, see recommendations for DVT/PE. **Clarification**: Classification of antiphospholipid syndrome includes presence of a clinical feature (e.g., thrombosis or obstetric morbidity) and persistently abnormal antiphospholipid antibody test on two or more occasions at least 12 weeks apart (*152*). **Evidence:** Among women with factor V Leiden mutation, prothrombin gene mutation, antithrombin deficiency, and protein C deficiency, COC users had an increased risk for venous and arterial thrombosis compared with nonusers. Evidence was inconsistent on risk for thrombosis among women with protein S deficiency using COCs. No evidence was identified on COC use among persons with antiphospholipid syndrome (Supplementary Appendix, https://stacks.cdc.gov/view/cdc/156516).
**Superficial venous disorders**			
a. Varicose veins	1		**Evidence:** One study suggested that among women with varicose veins, the rate of VTE and superficial venous thrombosis was higher in oral contraceptive users compared with nonusers; however, statistical significance was not reported and the number of events was small (*153*).
b. Superficial venous thrombosis (acute or history)	3		**Clarification:** Superficial venous thrombosis might be associated with an increased risk for VTE. If a person has risk factors for concurrent DVT (e.g., thrombophilia or cancer) or has current or history of DVT, see recommendations for DVT/PE. Superficial venous thrombosis associated with a peripheral intravenous catheter is less likely to be associated with additional thrombosis and use of CHCs may be considered. **Evidence:** One study demonstrated that among women with superficial venous thrombosis, the risk for VTE was higher in oral contraceptive users compared with nonusers (*153*).
**Current and history of ischemic heart disease** This condition is associated with increased risk for adverse health events as a result of pregnancy (Box 3).	4		—
**Stroke** (history of cerebrovascular accident) This condition is associated with increased risk for adverse health events as a result of pregnancy (Box 3).	4		—
**Valvular heart disease** Complicated valvular heart disease is associated with increased risk for adverse health events as a result of pregnancy (Box 3).			
a. Uncomplicated	2		—
b. Complicated (pulmonary hypertension, risk for atrial fibrillation, or history of subacute bacterial endocarditis)	4		**Comment:** Among persons with valvular heart disease, CHC use might further increase the risk for arterial thrombosis; persons with complicated valvular heart disease are at greatest risk.
**Peripartum cardiomyopathy** This condition is associated with increased risk for adverse health events as a result of pregnancy (Box 3).			**Evidence:** No direct evidence exists about the safety of CHCs among women with peripartum cardiomyopathy. Limited indirect evidence from noncomparative studies of women with cardiac disease demonstrated few cases of hypertension and transient ischemic attack in women with cardiac disease using COCs. No cases of heart failure were reported (*154*). **Comment:** COCs might increase fluid retention in healthy persons; fluid retention might worsen heart failure in persons with peripartum cardiomyopathy. COCs might induce cardiac arrhythmias in healthy persons; persons with peripartum cardiomyopathy have a high incidence of cardiac arrhythmias.
a. Normal or mildly impaired cardiac function (New York Heart Association Functional Class I or II: no limitation of activities or slight, mild limitation of activity) (*155*)			
i. <6 months	4		
ii. ≥6 months	3		
b. Moderately or severely impaired cardiac function (New York Heart Association Functional Class III or IV: marked limitation of activity or should be at complete rest) (*155*)	4		
**Renal Disease**			
**Chronic kidney disease** This condition is associated with increased risk for adverse health events as a result of pregnancy (Box 3).			
a. Current nephrotic syndrome	4		**Evidence:** No direct evidence was identified on CHC use among persons with CKD with current nephrotic syndrome (Supplementary Appendix, https://stacks.cdc.gov/view/cdc/156516). Persons with severe CKD or nephrotic syndrome are at higher risk for thrombosis than the general population (*156**–**158*). Use of CHCs might further elevate risk for thrombosis among those with CKD with current nephrotic syndrome. **Comment:** A person might have CKD without current nephrotic syndrome but might have other conditions often associated with CKD (e.g., diabetes, hypertension, and SLE). See recommendations for other conditions if they apply.
b. Hemodialysis	4		**Evidence:** No direct evidence was identified on CHC use among persons with CKD on hemodialysis (Supplementary Appendix, https://stacks.cdc.gov/view/cdc/156516). Persons with CKD on dialysis are at higher risk for thrombosis than the general population (*156**–**158*). Use of CHCs might further elevate risk for thrombosis among those with CKD on dialysis. **Comment:** A person might have CKD without hemodialysis, but might have other conditions often associated with CKD (e.g., diabetes, hypertension, and SLE). See recommendations for other conditions if they apply.
c. Peritoneal dialysis	4		**Evidence:** No direct evidence was identified on CHC use among persons with CKD on peritoneal dialysis (Supplementary Appendix, https://stacks.cdc.gov/view/cdc/156516). Persons with CKD on dialysis are at higher risk for thrombosis than the general population (*156**–**158*). Use of CHCs might further elevate risk for thrombosis among those with CKD. **Comment:** A person might have CKD without peritoneal dialysis, but might have other conditions often associated with CKD (e.g., diabetes, hypertension, and SLE). See recommendations for other conditions if they apply.
**Rheumatic Diseases**			
**Systemic lupus erythematosus** This condition is associated with increased risk for adverse health events as a result of pregnancy (Box 3).			
a. Positive (or unknown) antiphospholipid antibodies	4		**Clarification:** Persons with SLE are at increased risk for ischemic heart disease, stroke, and VTE. Categories assigned to such conditions in U.S. MEC should be the same for persons with SLE who have these conditions. For all subconditions of SLE, classifications are based on the assumption that no other risk factors for cardiovascular disease are present; these classifications must be modified in the presence of such risk factors (*159**–**177*). **Evidence:** Antiphospholipid antibodies are associated with a higher risk for both arterial and venous thrombosis (*178**,**179*).
b. Severe thrombocytopenia	2		**Clarification:** Persons with SLE are at increased risk for ischemic heart disease, stroke, and VTE. Categories assigned to such conditions in U.S. MEC should be the same for persons with SLE who have these conditions. For all subconditions of SLE, classifications are based on the assumption that no other risk factors for cardiovascular disease are present; these classifications must be modified in the presence of such risk factors (*159**–**177*).
c. Immunosuppressive therapy	2		**Clarification:** Persons with SLE are at increased risk for ischemic heart disease, stroke, and VTE. Categories assigned to such conditions in U.S. MEC should be the same for persons with SLE who have these conditions. For all subconditions of SLE, classifications are based on the assumption that no other risk factors for cardiovascular disease are present; these classifications must be modified in the presence of such risk factors (*159**–**177*).
d. None of the above	2		**Clarification:** Persons with SLE are at increased risk for ischemic heart disease, stroke, and VTE. Categories assigned to such conditions in U.S. MEC should be the same for persons with SLE who have these conditions. For all subconditions of SLE, classifications are based on the assumption that no other risk factors for cardiovascular disease are present; these classifications must be modified in the presence of such risk factors (*159**–**177*).
**Rheumatoid arthritis**			**Evidence:** Limited evidence demonstrates no consistent pattern of improvement or worsening of rheumatoid arthritis with use of oral contraceptives, progesterone, or estrogen (*180*).
a. Not receiving immunosuppressive therapy	2		
b. Receiving immunosuppressive therapy	2		
**Neurologic Conditions**			
**Headaches**			
a. Nonmigraine (mild or severe)	1		**Clarification:** Classification depends on accurate diagnosis of those severe headaches that are migraines and those headaches that are not, as well as diagnosis of ever experiencing aura. Aura is a specific focal neurologic symptom. For more information about headache classification see the International Headache Society’s *International Classification of Headache Disorders, 3rd ed.* (https://ichd-3.org) (*181*). Any new headaches or marked changes in headaches should be evaluated.
b. Migraine			**Clarification:** Classification depends on accurate diagnosis of those severe headaches that are migraines and those headaches that are not, as well as diagnosis of ever experiencing aura. Aura is a specific focal neurologic symptom. For more information about headache classification see the International Headache Society’s *International Classification of Headache Disorders, 3rd ed.* (https://ichd-3.org) (*181*). Any new headaches or marked changes in headaches should be evaluated. **Clarification:** Classification is for persons without any other risk factors for stroke (e.g., age, hypertension, and smoking). **Evidence:** Among women with migraine, oral contraceptive use is associated with about a threefold increased risk for ischemic stroke compared with nonuse, although most studies did not specify migraine type or oral contraceptive formulation. The only study to examine migraine type found that the risk for ischemic stroke among women with migraine with aura was increased to a similar level among both oral contraceptive users and nonusers, compared with women without migraine (*182*). The risk for ischemic stroke is increased among women using COCs, compared with women not using COCs (*101**,**183*). The risk for ischemic stroke is also increased among women with migraine with aura, compared with women without migraine (*184**–**186*). One older meta-analysis found that migraine without aura was associated with an increased risk for ischemic stroke, while two more recent meta-analyses did not find such an association (*184**–**186*). **Comment:** Menstrual migraine is a subtype of migraine without aura. For more information, see the International Headache Society’s *International Classification of Headache Disorders, 3rd ed.* (https://ichd-3.org) (*181*).
i. Without aura (includes menstrual migraine)	2		
ii. With aura	4		
**Epilepsy** This condition is associated with increased risk for adverse health events as a result of pregnancy (Box 3).	1		**Clarification:** If a person is taking anticonvulsants, see recommendations for Drug Interactions. Certain anticonvulsants lower COC effectiveness. The extent to which patch or ring use is similar to COC use in this regard remains unclear.
**Multiple sclerosis**			**Evidence:** Limited evidence suggests that use of COCs or oral contraceptives (type not specified) among women with multiple sclerosis does not worsen the clinical course of disease (*187*). **Comment:** No data exist that evaluate the increased risk for VTE among persons with multiple sclerosis using CHCs. However, persons with multiple sclerosis are at higher risk for VTE than those without multiple sclerosis.
a. Without prolonged immobility	1		
b. With prolonged immobility	3		
**Depressive Disorders**			
**Depressive disorders**	1		**Clarification**: If a person is receiving psychotropic medications or St. John’s wort, see recommendations for Drug Interactions. **Evidence**: COC use was not associated with increased depressive symptoms in women with depression or scoring above threshold levels on a validated depression screening instrument compared with baseline or with nonusers with depression. One small study of women with bipolar disorder found that oral contraceptives did not significantly change mood across the menstrual cycle (*188*).
**Reproductive Tract Infections and Disorders**			
**Vaginal bleeding patterns**			
a. Irregular pattern without heavy bleeding	1		**Comment:** Irregular menstrual bleeding patterns are common among healthy persons.
b. Heavy or prolonged bleeding (includes regular and irregular patterns)	1		**Clarification:** Unusually heavy bleeding should raise the suspicion of a serious underlying condition. **Evidence:** A Cochrane Collaboration Review identified one RCT evaluating the effectiveness of COC use compared with naproxen and danazol in treating menorrhagia. Women with menorrhagia did not report worsening of the condition or any adverse events related to COC use (*189*).
**Unexplained vaginal bleeding** (suspicious for serious condition) before evaluation	2		**Clarification:** If pregnancy or an underlying pathological condition (e.g., pelvic malignancy) is suspected, it must be evaluated and the category adjusted after evaluation. **Comment:** No conditions that cause vaginal bleeding will be worsened in the short-term by use of CHCs.
**Endometriosis**	1		**Evidence:** A Cochrane Collaboration Review identified one RCT evaluating the effectiveness of COC use compared with a gonadotropin-releasing hormone analog in treating the symptoms of endometriosis. Women with endometriosis did not report worsening of the condition or any adverse events related to COC use (*190*).
**Benign ovarian tumors** (including cysts)	1		—
**Severe dysmenorrhea**	1		**Evidence:** Risk for side effects with COC use was not higher among women with dysmenorrhea than among women not using COCs. Certain COC users had a reduction in pain and bleeding (*191**,**192*).
**Gestational trophoblastic disease** This condition is associated with increased risk for adverse health events as a result of pregnancy (Box 3).			**Clarification:** For all subconditions of gestational trophoblastic disease, classifications are based on the assumption that persons are under close medical supervision because of the need for monitoring of β-hCG levels for appropriate disease surveillance. **Evidence:** After molar pregnancy evacuation, the balance of evidence found COC use did not increase the risk for postmolar trophoblastic disease, and β–hCG levels regressed more rapidly in certain COC users than in nonusers (193). Limited evidence suggests that use of COCs during chemotherapy does not significantly affect the regression or treatment of postmolar trophoblastic disease compared with women who used a nonhormonal contraceptive method or DMPA during chemotherapy (*193*).
a. Suspected gestational trophoblastic disease (immediate postevacuation)			
i. Uterine size first trimester	1		
ii. Uterine size second trimester	1		
b. Confirmed gestational trophoblastic disease (after initial evacuation and during monitoring)			
i. Undetectable or nonpregnant β-hCG levels	1		
ii. Decreasing β-hCG levels	1		
iii. Persistently elevated β-hCG levels or malignant disease, with no evidence or suspicion of intrauterine disease	1		
iv. Persistently elevated β-hCG levels or malignant disease, with evidence or suspicion of intrauterine disease	1		
**Cervical ectropion**	1		**Comment:** Cervical ectropion is not a risk factor for cervical cancer, and restriction of CHC use is unnecessary.
**Cervical intraepithelial neoplasia**	2		**Evidence:** Among women with persistent human papillomavirus infection, long-term COC use (≥5 years) might increase the risk for carcinoma in situ and invasive carcinoma (*194*). Limited evidence on women with low-grade squamous intraepithelial lesions found use of the vaginal ring did not worsen the condition (*9*).
**Cervical cancer** (awaiting treatment)	2		**Comment:** Theoretical concern exists that CHC use might affect prognosis of the existing disease. While awaiting treatment, persons may use CHCs. In general, treatment of this condition can render a person infertile.
**Breast disease** Breast cancer is associated with increased risk for adverse health events as a result of pregnancy (Box 3).			
a. Undiagnosed mass	2		**Clarification:** Evaluation of mass should be pursued as early as possible.
b. Benign breast disease	1		—
c. Family history of cancer	1		**Evidence:** Women with breast cancer susceptibility genes (e.g., *BRCA1* and *BRCA2*) have a higher baseline risk for breast cancer than women without these genes. The baseline risk for breast cancer also is higher among women with a family history of breast cancer than among those who do not have such a history. However, evidence does not suggest that the increased risk for breast cancer among women with either a family history of breast cancer or breast cancer susceptibility genes is modified by the use of COCs (*195**–**212*).
d. Breast cancer			**Comment:** Breast cancer is a hormonally sensitive tumor, and the prognosis for persons with current or recent breast cancer might worsen with CHC use.
i. Current	4		
ii. Past and no evidence of current disease for 5 years	3		
**Endometrial hyperplasia**	1		—
**Endometrial cancer** This condition is associated with increased risk for adverse health events as a result of pregnancy (Box 3).	1		**Comment:** COC use reduces the risk for endometrial cancer; whether patch or ring use reduces the risk for endometrial cancer is not known. While awaiting treatment, patients may use CHCs. In general, treatment of this condition can render a person infertile.
**Ovarian cancer** This condition is associated with increased risk for adverse health events as a result of pregnancy (Box 3).	1		**Comment:** COC use reduces the risk for ovarian cancer; whether patch or ring use reduces the risk for ovarian cancer is not known. While awaiting treatment, patients may use CHCs. In general, treatment of this condition can render a person infertile.
**Uterine fibroids**	1		**Comment:** COCs do not appear to cause growth of uterine fibroids, and patch and ring also are not expected to cause growth.
**Pelvic inflammatory disease**			**Comment:** COCs might reduce the risk for PID among persons with STIs but do not protect against HIV infection or lower genital tract STIs. Whether use of patch or ring reduces the risk for PID among persons with STIs is unknown; however, they do not protect against HIV infection or lower genital tract STIs.
a. Current PID	1		
b. Past PID			
i. With subsequent pregnancy	1		
ii. Without subsequent pregnancy	1		
**Sexually transmitted infections**			
a. Current purulent cervicitis or chlamydial infection or gonococcal infection	1		—
b. Vaginitis (including *Trichomonas vaginalis* and bacterial vaginosis)	1		—
c. Other factors related to STIs	1		—
**HIV**			
**High risk for HIV infection**	1		**Evidence:** Low-to-moderate-quality evidence from 11 observational studies suggested no association between COC use (it was assumed that studies that did not specify oral contraceptive type examined mostly, if not exclusively, COC use) and HIV acquisition. No studies of patch or ring were identified (*213**,**214*).
**HIV infection** For persons with HIV infection who are not clinically well or not receiving ARV therapy, this condition is associated with increased risk for adverse health events as a result of pregnancy (Box 3).	1		**Clarification:** Drug interactions might exist between hormonal contraceptives and ARV drugs; see recommendations for Drug Interactions. **Evidence:** Overall, evidence does not support an association between COC use and progression of HIV. Limited direct evidence does not support an association between COC use and transmission of HIV to noninfected partners; studies measuring genital viral shedding as a proxy for infectivity have had mixed results. Studies measuring whether hormonal contraceptive methods affect plasma HIV viral load generally have found no effect (*215**–**217*).
**Other Infections**			
**Schistosomiasis** Schistosomiasis with fibrosis of the liver is associated with increased risk for adverse health events as a result of pregnancy (Box 3).			
a. Uncomplicated	1		**Evidence:** Among women with uncomplicated schistosomiasis, COC use had no adverse effects on liver function (*218**–**224*).
b. Fibrosis of the liver (if severe, see recommendations for Cirrhosis)	1		—
**Tuberculosis** This condition is associated with increased risk for adverse health events as a result of pregnancy (Box 3).			**Clarification:** If a person is taking rifampin, see recommendations for Drug Interactions. Rifampin is likely to decrease COC effectiveness. The extent to which patch or ring use is similar to COC use in this regard remains unclear.
a. Nonpelvic	1		
b. Pelvic	1		
**Malaria**	1		—
**Endocrine Conditions**			
**Diabetes** Insulin-dependent diabetes; diabetes with nephropathy, retinopathy, or neuropathy; diabetes with other vascular disease; or diabetes of >20 years’ duration are associated with increased risk for adverse health events as a result of pregnancy (Box 3).			
a. History of gestational disease	1		**Evidence:** The development of non–insulin-dependent diabetes in women with a history of gestational diabetes is not increased by use of COCs (*225**–**232*). Likewise, lipid levels appear to be unaffected by COC use (*233**–**235*).
b. Nonvascular disease			**Evidence:** Among women with insulin-dependent or non–insulin-dependent diabetes, COC use had limited effect on daily insulin requirements and no effect on long-term diabetes control (e.g., glycosylated hemoglobin levels) or progression to retinopathy. Changes in lipid profile and hemostatic markers were limited, and most changes remained within normal values (*236**–**245*).
i. Non-insulin dependent	2		—
ii. Insulin dependent	2		—
c. Nephropathy, retinopathy, or neuropathy	3/4		**Clarification:** The category should be assessed according to the severity of the condition.
d. Other vascular disease or diabetes of >20 years’ duration	3/4		**Clarification:** The category should be assessed according to the severity of the condition.
**Thyroid disorders**			
a. Simple goiter	1		—
b. Hyperthyroid	1		—
c. Hypothyroid	1		—
**Gastrointestinal Conditions**			
**Inflammatory bowel disease** (ulcerative colitis or Crohn’s disease)	2/3		**Clarification:** For persons with mild IBD and with no other risk factor for VTE, the benefits of CHC use generally outweigh the risks (category 2). However, for persons with IBD who are at increased risk for VTE (e.g., those with active or extensive disease, surgery, immobilization, corticosteroid use, vitamin deficiencies, or fluid depletion), the risks of CHC use generally outweigh the benefits (category 3). **Evidence:** Risk for disease relapse was not significantly higher among women with IBD using oral contraceptives (most studies did not specify type) than among nonusers (*246*). Absorption of COCs among women with mild ulcerative colitis and no or small ileal resections was similar to the absorption among healthy women (*246*). Findings might not apply to women with Crohn’s disease or more extensive bowel resections. No data exist that evaluate the increased risk for VTE among women with IBD using CHCs. However, women with IBD are at higher risk than unaffected women for VTE (*246*).
**Gallbladder disease**			**Comment:** CHCs might cause a small increased risk for gallbladder disease. CHCs might worsen existing gallbladder disease.
a. Asymptomatic	2		
b. Symptomatic			
i. Current	3		
ii. Treated by cholecystectomy	2		
iii. Medically treated	3		
**History of cholestasis**			
a. Pregnancy related	2		**Comment:** History of pregnancy-related cholestasis might predict an increased risk for COC-related cholestasis.
b. Past COC related	3		**Comment:** History of COC-related cholestasis predicts an increased risk with subsequent COC use.
**Viral hepatitis**	Initiation	Continuation	—
a. Acute or flare	3/4	2	**Clarification (initiation):** The category should be assessed according to the severity of the condition. **Evidence:** Limited evidence was identified on COC use among persons with acute viral hepatitis. Data suggest that in women with chronic viral hepatitis, COC use does not increase the risk or severity of fibrosis, nor does it increase the risk for hepatocellular carcinoma. For women with chronic viral hepatitis, COC use does not appear to trigger severe liver dysfunction (Supplementary Appendix, https://stacks.cdc.gov/view/cdc/156516). **Comment:** Hepatic metabolism of exogenous hormones might be impaired in persons with liver dysfunction, which could lead to increased estrogen levels in circulation and estrogen-related side effects and adverse events (e.g., thrombosis).
b. Chronic	1	1	**Evidence:** Data suggest that in women with chronic viral hepatitis, COC use does not increase the risk or severity of fibrosis, nor does it increase the risk for hepatocellular carcinoma. For women with chronic viral hepatitis, COC use does not appear to trigger severe liver dysfunction (Supplementary Appendix, https://stacks.cdc.gov/view/cdc/156516).
**Cirrhosis** Decompensated cirrhosis is associated with increased risk for adverse health events as a result of pregnancy (Box 3).			
a. Compensated (normal liver function)	1		**Evidence**: No direct evidence was identified on CHC use among persons with compensated cirrhosis (Supplementary Appendix, https://stacks.cdc.gov/view/cdc/156516).
b. Decompensated (impaired liver function)	4		**Evidence**: No direct evidence was identified on CHC use among persons with decompensated cirrhosis (Supplementary Appendix, https://stacks.cdc.gov/view/cdc/156516). **Comment:** Hepatic metabolism of exogenous hormones might be impaired in persons with liver dysfunction, which could lead to increased estrogen levels in circulation and estrogen-related side effects and adverse events (e.g., thrombosis). Any estrogen-related hepatotoxicity might be less tolerated in persons with existing liver dysfunction.
**Liver tumors** Hepatocellular adenoma and malignant liver tumors are associated with increased risk for adverse health events as a result of pregnancy (Box 3).			
a. Benign			
i. Focal nodular hyperplasia	2		**Evidence:** Limited evidence suggests that COC use does not influence either progression or regression of focal nodular hyperplasia (Supplementary Appendix, https://stacks.cdc.gov/view/cdc/156516).
ii. Hepatocellular adenoma	4		**Evidence:** Evidence suggests that COC use is associated with progression of hepatocellular adenoma growth, while COC discontinuation is associated with stability or regression (Supplementary Appendix, https://stacks.cdc.gov/view/cdc/156516).
b. Malignant (hepatocellular carcinoma)	4		**Evidence:** No direct evidence was identified on CHC use among persons with hepatocellular carcinoma (Supplementary Appendix, https://stacks.cdc.gov/view/cdc/156516).
**Respiratory Conditions**			
**Cystic fibrosis** This condition is associated with increased risk for adverse health events as a result of pregnancy (Box 3).	1		**Clarification:** Persons with cystic fibrosis are at increased risk for diabetes, liver disease, gallbladder disease, and VTE (particularly related to use of central venous catheters) and are frequently prescribed antibiotics. Categories assigned to such conditions in U.S. MEC should be the same for persons with cystic fibrosis who have these conditions. For cystic fibrosis, classifications are based on the assumption that no other conditions are present; these classifications must be modified in the presence of such conditions. **Clarification:** Certain drugs to treat cystic fibrosis (e.g., lumacaftor) might reduce effectiveness of hormonal contraceptives, including oral, injectable, transdermal, and implantable contraceptives. **Evidence:** Limited evidence suggests that use of COCs or oral contraceptives (type not specified) among women with cystic fibrosis is not associated with worsening of disease severity. Very limited evidence suggests that cystic fibrosis does not impair the effectiveness of hormonal contraception (*247*).
**Hematologic Conditions**			
**Thalassemia**	1		**Comment:** Anecdotal evidence from countries where thalassemia is prevalent indicates that COC use does not worsen the condition.
**Sickle cell disease** This condition is associated with increased risk for adverse health events as a result of pregnancy (Box 3).	4		**Evidence**: Persons with sickle cell disease are at higher risk for stroke and venous thrombosis than the general population (*248**–**251*). CHC use might further elevate risk for thrombosis among persons with sickle cell disease, but evidence is limited (Supplementary Appendix, https://stacks.cdc.gov/view/cdc/156516).
**Iron deficiency anemia**	1		**Comment:** CHC use might decrease menstrual blood loss.
**Solid Organ Transplantation**			
**Solid organ transplantation** This condition is associated with increased risk for adverse health events as a result of pregnancy (Box 3).			
a. No graft failure	2		**Clarification:** Persons with transplant due to Budd-Chiari syndrome should not use CHCs because of the increased risk for thrombosis. **Evidence:** Limited evidence among CHC users indicated no adverse events and no overall changes in biochemical parameters (e.g., blood pressure, cholesterol) and no pregnancies (Supplementary Appendix, https://stacks.cdc.gov/view/cdc/156516). However, one study reported discontinuations of COC use in two (8%) of 26 women as a result of serious medical complications, including acute graft rejection (Supplementary Appendix, https://stacks.cdc.gov/view/cdc/156516).
b. Graft failure	4		**Evidence:** Limited evidence among CHC users indicated no adverse events and no overall changes in biochemical parameters (e.g., blood pressure, cholesterol) and no pregnancies (Supplementary Appendix, https://stacks.cdc.gov/view/cdc/156516). However, one study reported discontinuations of COC use in two (8%) of 26 women as a result of serious medical complications, including acute graft rejection (Supplementary Appendix, https://stacks.cdc.gov/view/cdc/156516).
**Drug Interactions**			
**Antiretrovirals used for prevention (PrEP) or treatment of HIV infection**			**Comment:** These recommendations generally are for ARV agents used alone. However, most persons receiving ARV therapy are using multiple drugs in combination. In general, whether interactions between ARVs and hormonal contraceptives differ when ARVs are given alone or in combination is unknown.
See the following guidelines for the most up-to-date recommendations on drug-drug interactions between hormonal contraception and ARVs: 1) Recommendations for the Use of Antiretroviral Drugs During Pregnancy and Interventions to Reduce Perinatal HIV Transmission in the United States (https://clinicalinfo.hiv.gov/en/guidelines/perinatal/prepregnancy-counseling-childbearing-age-overview?view=full#table-3) (*252*) and 2) Guidelines for the Use of Antiretroviral Agents in Adults and Adolescents with HIV (https://clinicalinfo.hiv.gov/en/guidelines/hiv-clinical-guidelines-adult-and-adolescent-arv/drug-interactions-overview?view=full) (*253*).			
a. Nucleoside reverse transcriptase inhibitors (NRTIs)			
i. Abacavir (ABC)	1		**Evidence:** NRTIs do not appear to have significant risk for interactions with hormonal contraceptive methods (*254**–**259*).
ii. Tenofovir (TDF)	1		
iii. Zidovudine (AZT)	1		
iv. Lamivudine (3TC)	1		
v. Didanosine (DDI)	1		
vi. Emtricitabine (FTC)	1		
vii. Stavudine (D4T)	1		
b. Nonnucleoside reverse transcriptase inhibitors (NNRTIs)			
i. Efavirenz (EFV)	2		**Clarification:** Evidence suggests drug interactions between EFV and certain hormonal contraceptives. These interactions might reduce the effectiveness of the hormonal contraceptive. **Evidence:** Two studies suggested that pregnancy rates might be higher among women using COCs and EFV compared with COCs alone, although one study found no difference in pregnancy rates (*260**–**262*). Two studies found conflicting results on ovulations in women receiving COCs and EFV compared with EFV alone (*263**,**264*). Two pharmacokinetic studies demonstrated decreases in ethinyl estradiol and progestin concentrations in women receiving COCs and EFV compared with COCs alone (*264**,**265*). Pharmacokinetic studies demonstrated generally no changes in EFV concentrations with concomitant COC use (*264**–**266*).
ii. Etravirine (ETR)	1		**Evidence:** One study demonstrated no clinically relevant pharmacokinetic or pharmacodynamic changes in women using COCs and ETR compared with COCs alone (*267*).
iii. Nevirapine (NVP)	1		**Evidence:** Five studies found no significant differences in pregnancy rates among women using COCs and NVP compared with women using COCs alone (*260**–**262**,**266**,**268*). Three studies reported no ovulations among women receiving COCs and NVP (*263**,**268**,**269*). Two pharmacokinetic studies demonstrated decreased concentrations of ethinyl estradiol and progestin among women using COCs and NVP compared with COCs alone, and one study found no change in contraceptive hormone concentrations (*263**,**269**,**270*). Pharmacokinetic studies demonstrated generally no changes in NVP concentrations with concomitant COC use (*263**,**270**,**271*).
iv. Rilpivirine (RPV)	1		**Evidence:** One study demonstrated no clinical significant pharmacokinetic changes or adverse events in women using COCs and RPV compared with COCs alone (*272*).
c. Ritonavir-boosted protease inhibitors			
i. Ritonavir-boosted atazanavir (ATV/r)	2		**Clarification:** Theoretically, drug interactions might occur between certain ritonavir-boosted protease inhibitors and certain hormonal contraceptives that might reduce the effectiveness of the hormonal contraceptive. **Evidence:** One pharmacokinetic study demonstrated decreased estrogen but increased progestin concentrations in women using COCs and ATV/r compared with COCs alone (*273*).
ii. Ritonavir-boosted darunavir (DRV/r)	2		**Clarification:** Theoretically, drug interactions might occur between certain ritonavir-boosted protease inhibitors and certain hormonal contraceptives that might reduce the effectiveness of the hormonal contraceptive. **Evidence:** One pharmacokinetic study demonstrated no change in follicle-stimulating hormone or luteinizing hormone but decreases in ethinyl estradiol and norethindrone in women using COCs with DRV/r compared with COCs alone (*274*).
iii. Ritonavir-boosted fosamprenavir (FPV/r)	2		**Clarification:** Theoretically, drug interactions might occur between certain ritonavir-boosted protease inhibitors and certain hormonal contraceptives that might reduce the effectiveness of the hormonal contraceptive. **Evidence:** Information from the package label states that both ethinyl estradiol and norethindrone concentrations decreased with concurrent administration of COCs and FPV/r (*275*).
iv. Ritonavir-boosted lopinavir (LPV/r)	1		**Evidence:** One study demonstrated a nonsignificant increase in pregnancy rates among women using COCs and LPV/r compared with COCs alone (*260*). One study demonstrated no ovulations in women using the combined hormonal patch and LPV/r compared with combined hormonal patch alone; ethinyl estradiol concentrations for COC and patch users decreased but norelgestromin concentrations increased with use of the patch (*276*).
v. Ritonavir-boosted saquinavir (SQV/r)	2		**Clarification:** Theoretically, drug interactions might occur between certain ritonavir-boosted protease inhibitors and certain hormonal contraceptives that might reduce the effectiveness of the hormonal contraceptive. **Evidence:** One pharmacokinetic study demonstrated no change in SQV concentrations in women using COC and SQV compared with COCs alone (*277*).
iv. Ritonavir-boosted tipranavir (TPV/r)	2		**Clarification:** Theoretically, drug interactions might occur between certain ritonavir-boosted protease inhibitors and certain hormonal contraceptives that might reduce the effectiveness of the hormonal contraceptive. **Evidence:** Information from the package label states that ethinyl estradiol concentrations decrease but norethindrone concentrations increased with concurrent administration of COCs and TPV/r (*278*).
d. Protease inhibitors without ritonavir			
i. Atazanavir (ATV)	2		**Clarification:** Theoretical concern exists that increased levels of ethinyl estradiol because of interactions with ATV might increase the risk for adverse events. **Evidence:** Information from the package label states that there are inconsistent changes in ethinyl estradiol concentrations and increases in progestin concentrations with concurrent administration of two different COCs and ATV (*279*). **Comment:** When ATV is administered with cobicistat, theoretical concern exists for a drug interaction with hormonal contraceptives. Cobicistat is an inhibitor of CYP3A and CYP2D6 and could theoretically increase contraceptive hormone levels. However, its effects on CYP enzymes and drug levels might vary when combined with other ARVs.
ii. Fosamprenavir (FPV)	3		**Clarification:** Concern exists that interactions between FPV and hormonal contraceptives leading to decreased levels of FPV might diminish effectiveness of the ARV drug. **Evidence:** Information from the package label states that amprenavir concentrations decreased with concurrent administration of COCs and amprenavir. Norethindrone concentrations increased and ethinyl estradiol concentrations did not change (*275*).
iii. Indinavir (IDV)	1		**Evidence:** One small study found no pregnancies in women using COCs and IDV (*262*).
iv. Nelfinavir (NFV)	2		**Clarification:** Evidence suggests drug interactions between certain protease inhibitors and certain hormonal contraceptives. These interactions might reduce the effectiveness of the hormonal contraceptive. **Evidence:** One small study suggested that women using COCs and NFV might have had higher pregnancy rates than those using COCs alone (*262*).
e. CCR5 co-receptor antagonists			
i. Maraviroc (MVC)	1		**Evidence:** COC concentrations were not altered by co-administration with MVC (*280*).
f. HIV integrase strand transfer inhibitors			
i. Raltegravir (RAL)	1		**Evidence:** One pharmacokinetic study demonstrated increased concentrations of norgestimate and no change in ethinyl estradiol among women using COCs and RAL compared with COCs alone (*281*).
ii. Dolutegravir (DTG)	1		**Evidence:** One study demonstrated no clinically relevant pharmacokinetic or pharmacodynamic changes in women using COCs and DTG compared with COCs alone (*282*).
iii. Elvitegravir (EVG)	1		**Evidence:** Information from the package label states that ethinyl estradiol concentrations decreased and norgestimate concentrations increased with concurrent administration of COCs and EVG (*283*). **Comment:** When EVG is administered with cobicistat, theoretical concern exists for a drug interaction with hormonal contraceptives. Cobicistat is an inhibitor of CYP3A and CYP2D6 and could theoretically increase contraceptive hormone levels. However, its effects on CYP enzymes and drug levels might vary when combined with other ARVs.
g. Fusion inhibitors			
i. Enfuvirtide	1		—
**Anticonvulsant therapy**			
a. Certain anticonvulsants (phenytoin, carbamazepine, barbiturates, primidone, topiramate, oxcarbazepine)	3		**Clarification:** Although the interaction of certain anticonvulsants with CHCs is not harmful, it is likely to reduce the effectiveness of CHCs. Use of other contraceptives should be encouraged for persons who are long-term users of any of these drugs. When a COC is chosen, a preparation containing a minimum of 30 *μ*g ethinyl estradiol should be used. **Evidence:** Use of certain anticonvulsants might decrease the effectiveness of COCs (*284**–**288*).
b. Lamotrigine	3		**Clarification:** The recommendation for lamotrigine applies only for situations where lamotrigine monotherapy is taken concurrently with COCs. Anticonvulsant treatment regimens that combine lamotrigine and non–enzyme-inducing antiepileptic drugs (e.g., sodium valproate) do not interact with COCs. **Evidence:** Pharmacokinetic studies demonstrate levels of lamotrigine decrease significantly during COC use (*288**–**293*). Certain women who used both COCs and lamotrigine experienced increased seizure activity in one trial (*289*).
**Antimicrobial therapy**			
a. Broad-spectrum antibiotics	1		**Evidence:** Most broad-spectrum antibiotics do not affect the contraceptive effectiveness of COCs (*294**–**330*), patch (*331*), or ring (*332*).
b. Antifungals	1		**Evidence:** Studies of antifungal agents have demonstrated no clinically significant pharmacokinetic interactions with COCs (*333**–**342*) or ring (*343*).
c. Antiparasitics	1		**Evidence:** Studies of antiparasitic agents have demonstrated no clinically significant pharmacokinetic interactions with COCs (*218**,**344**–**348*).
d. Rifampin or rifabutin therapy	3		**Clarification:** Although the interaction of rifampin or rifabutin therapy with CHCs is not harmful, it is likely to reduce the effectiveness of CHCs. Use of other contraceptives should be encouraged for persons who are long-term users of either of these drugs. When a COC is chosen, a preparation containing a minimum of 30 *μ*g ethinyl estradiol should be used. **Evidence:** The balance of the evidence suggests that rifampin reduces the effectiveness of COCs (*349**–**363*). Data on rifabutin are limited, but effects on metabolism of COCs are less than with rifampin, and small studies have not demonstrated evidence of ovulation (*351**,**357*).
**Psychotropic medications**			**Comment:** For many common psychotropic agents, limited or no theoretical concern exists for clinically significant drug interactions when co-administered with hormonal contraceptives. However, either no or very limited data exist examining potential interactions for these classes of medications. For psychotropic agents that are CYP1A2 substrates (e.g., duloxetine, mirtazapine, ziprasidone, olanzapine, clomipramine, imipramine, and amitriptyline), co-administration with CHCs could theoretically yield increased concentrations of the psychotropic drug. For agents with narrow therapeutic windows (e.g., tricyclic antidepressants), increased drug concentrations might pose safety concerns that could necessitate closer monitoring.
a. Selective serotonin reuptake inhibitors (SSRIs)	1		**Evidence:** Limited clinical and pharmacokinetic data do not demonstrate concern for SSRIs decreasing the effectiveness of oral contraceptives. Limited evidence suggests that for women taking SSRIs, the use of hormonal contraceptives was not associated with differences in effectiveness of the SSRI for treatment or in adverse events when compared with women not taking hormonal contraceptives (*364*). **Comment:** Drugs that are inhibitors of CYP3A4 or CYP2C9 theoretically have the potential to increase levels of contraceptive steroids which might increase adverse events. Fluvoxamine is an SSRI known to be a moderate inhibitor of both CYP3A4 and CYP2C9; however, no clinical or pharmacokinetic studies were identified to explore potential drug-drug interactions.
**St. John’s wort**	2		**Evidence:** Although clinical data are limited, studies with pharmacokinetic and pharmacodynamics outcomes raise concern that St. John’s wort might decrease effectiveness of hormonal contraceptives, including increased risk for breakthrough bleeding and ovulation and increased metabolism of estrogen and progestins. Any interactions might be dependent on the dose of St. John’s wort, and the concentration of active ingredients across types of St. John’s wort preparations might vary (*365*).

**Abbreviations:** ARV = antiretroviral; BMD = bone mineral density; BMI = body mass index; CHC = combined hormonal contraceptive; CKD = chronic kidney disease; COC = combined oral contraceptive; CYP = cytochrome P450; DMPA = depot medroxyprogesterone acetate; DVT = deep venous thrombosis; hCG = human chorionic gonadotropin; HDL = high-density lipoprotein; IBD = inflammatory bowel disease; IUD = intrauterine device; LDL = low-density lipoprotein; NA = not applicable; PE = pulmonary embolism; PID = pelvic inflammatory disease; POC = progestin-only contraceptive; PrEP = pre-exposure prophylaxis; RCT = randomized clinical trial; SLE = systemic lupus erythematosus; SSRI = selective serotonin reuptake inhibitor; STI = sexually transmitted infection; U.S. MEC *= U.S. Medical Eligibility Criteria for Contraceptive Use*; U.S. SPR = *U.S. Selected Practice Recommendations for Contraceptive Use*; VTE = venous thromboembolism.

## Appendix E Classifications for Barrier Methods

Classifications for barrier contraceptive methods include those for condoms, which include external (male) condoms (latex or synthetic) and internal (female) condoms, spermicide and vaginal pH modulator, and diaphragm with spermicide and cervical cap with spermicide (Box E1) (Table E1).

Patients should be counseled that consistent and correct use of external (male) latex condoms reduces the risk for sexually transmitted infections (STIs), including HIV infection (*1*). Use of internal (female) condoms can provide protection from transmission of STIs, although data are limited (*1*). Patients also should be counseled that pre-exposure prophylaxis, when taken as prescribed, is highly effective for preventing HIV infection (*2*).

BOX E1 Categories for classifying barrier methods U.S. MEC 1 = A condition for which there is no restriction for the use of the contraceptive method U.S. MEC 2 = A condition for which the advantages of using the method generally outweigh the theoretical or proven risks U.S. MEC 3 = A condition for which the theoretical or proven risks usually outweigh the advantages of using the method U.S. MEC 4 = A condition that represents an unacceptable health risk if the contraceptive method is used **Abbreviation:** U.S. MEC = *U.S. Medical Eligibility Criteria for Contraceptive Use*.

**TABLE E1:** Classifications for barrier methods, including condoms, spermicide and vaginal pH modulator, and diaphragm with spermicide and cervical cap with spermicide

Condition	Category			Clarification/Evidence/Comment
	Condom	Spermicide/Vaginal pH modulator	Diaphragm/Cap (with spermicide)	
**Personal Characteristics and Reproductive History**				
**Pregnancy**	NA	NA	NA	**Clarification:** None of these methods are relevant for contraception during known pregnancy. However, for persons who remain at risk for STIs or HIV infection during pregnancy, the correct and consistent use of condoms is recommended.
**Age**				
a. Menarche to <40 years	1	1	1	—
b. ≥40 years	1	1	1	—
**Parity**				
a. Nulliparous	1	1	1	—
b. Parous	1	1	2	**Clarification:** Risk for cervical cap failure is higher in parous persons than in nulliparous persons.
**Postpartum** (breastfeeding and nonbreastfeeding)				
a. <6 weeks postpartum	1	1	NA	**Clarification:** Diaphragm and cap are unsuitable until uterine involution is complete.
b. ≥6 weeks postpartum	1	1	1	—
**Postabortion **(spontaneous or induced)				
a. First trimester abortion	1	1	1	—
b. Second trimester abortion	1	1	1	**Clarification:** Diaphragm and cap are unsuitable until 6 weeks after second trimester abortion.
c. Immediate postseptic abortion	1	1	1	—
**Past ectopic pregnancy**	1	1	1	—
**History of pelvic surgery**	1	1	1	—
**Smoking**				
a. Age <35 years	1	1	1	—
b. Age ≥35 years				
i. <15 cigarettes per day	1	1	1	—
ii. ≥15 cigarettes per day	1	1	1	—
**Obesity**				
a. BMI ≥30 kg/m^2^	1	1	1	—
b. Menarche to <18 years and BMI ≥30 kg/m^2^	1	1	1	—
**History of bariatric surgery** This condition is associated with increased risk for adverse health events as a result of pregnancy (Box 3).				
a. Restrictive procedures: decrease storage capacity of the stomach (vertical banded gastroplasty, laparoscopic adjustable gastric band, or laparoscopic sleeve gastrectomy)	1	1	1	—
b. Malabsorptive procedures: decrease absorption of nutrients and calories by shortening the functional length of the small intestine (Roux-en-Y gastric bypass or biliopancreatic diversion)	1	1	1	—
**Surgery**				
a. Minor surgery without immobilization	1	1	1	—
b. Major surgery				
i. Without prolonged immobilization	1	1	1	—
ii. With prolonged immobilization	1	1	1	—
**Cardiovascular Disease**				
**Multiple risk factors for atherosclerotic cardiovascular disease** (e.g., older age, smoking, diabetes, hypertension, low HDL, high LDL, or high triglyceride levels)	1	1	1	—
**Hypertension** Systolic blood pressure ≥160 mm Hg or diastolic blood pressure ≥100 mm Hg are associated with increased risk for adverse health events as a result of pregnancy (Box 3).				
a. Adequately controlled hypertension	1	1	1	—
b. Elevated blood pressure levels (properly taken measurements)				
i. Systolic 140–159 mm Hg or diastolic 90–99 mm Hg	1	1	1	—
ii. Systolic ≥160 mm Hg or diastolic ≥100 mm Hg	1	1	1	—
c. Vascular disease	1	1	1	—
**History of high blood pressure during pregnancy** (when current blood pressure is measurable and normal)	1	1	1	—
**Deep venous thrombosis/ Pulmonary embolism** This condition is associated with increased risk for adverse health events as a result of pregnancy (Box 3).				
a. Current or history of DVT/PE, receiving anticoagulant therapy (therapeutic dose) (e.g., acute DVT/PE or long-term therapeutic dose)	1	1	1	—
b. History of DVT/PE, receiving anticoagulant therapy (prophylactic dose)				
i. Higher risk for recurrent DVT/PE (one or more risk factors)	1	1	1	—
• Thrombophilia (e.g., factor V Leiden mutation; prothrombin gene mutation; protein S, protein C, and antithrombin deficiencies; or antiphospholipid syndrome) • Active cancer (metastatic, receiving therapy, or within 6 months after clinical remission), excluding nonmelanoma skin cancer • History of recurrent DVT/PE				
ii. Lower risk for recurrent DVT/PE (no risk factors)	1	1	1	—
c. History of DVT/PE, not receiving anticoagulant therapy				
i. Higher risk for recurrent DVT/PE (one or more risk factors)	1	1	1	—
• History of estrogen-associated DVT/PE • Pregnancy-associated DVT/PE • Idiopathic DVT/PE • Thrombophilia (e.g., factor V Leiden mutation; prothrombin gene mutation; protein S, protein C, and antithrombin deficiencies; or antiphospholipid syndrome) • Active cancer (metastatic, receiving therapy, or within 6 months after clinical remission), excluding nonmelanoma skin cancer • History of recurrent DVT/PE				
ii. Lower risk for recurrent DVT/PE (no risk factors)	1	1	1	—
d. Family history (first-degree relatives)	1	1	1	—
**Thrombophilia** (e.g., factor V Leiden mutation; prothrombin gene mutation; protein S, protein C, and antithrombin deficiencies; or antiphospholipid syndrome) This condition is associated with increased risk for adverse health events as a result of pregnancy (Box 3).	1	1	1	**Clarification:** Routine screening in the general population before contraceptive initiation is not recommended.
**Superficial venous disorders**				
a. Varicose veins	1	1	1	—
b. Superficial venous thrombosis (acute or history)	1	1	1	—
**Current and history of ischemic heart disease** This condition is associated with increased risk for adverse health events as a result of pregnancy (Box 3).	1	1	1	—
**Stroke** (history of cerebrovascular accident) This condition is associated with increased risk for adverse health events as a result of pregnancy (Box 3).	1	1	1	—
**Valvular heart disease** Complicated valvular heart disease is associated with increased risk for adverse health events as a result of pregnancy (Box 3).				
a. Uncomplicated	1	1	1	—
b. Complicated (pulmonary hypertension, risk for atrial fibrillation, or history of subacute bacterial endocarditis)	1	1	2	—
**Peripartum cardiomyopathy** This condition is associated with increased risk for adverse health events as a result of pregnancy (Box 3).				
a. Normal or mildly impaired cardiac function (New York Heart Association Functional Class I or II: no limitation of activities or slight, mild limitation of activity) (*3*)				
i. <6 months	1	1	1	—
ii. ≥6 months	1	1	1	—
b. Moderately or severely impaired cardiac function (New York Heart Association Functional Class III or IV: marked limitation of activity or should be at complete rest) (*3*)	1	1	1	—
**Renal Disease**				
**Chronic kidney disease** This condition is associated with increased risk for adverse health events as a result of pregnancy (Box 3).				
a. Current nephrotic syndrome	1	1	1	—
b. Hemodialysis	1	1	1	—
c. Peritoneal dialysis	1	1	1	—
**Rheumatic Diseases**				
**Systemic lupus erythematosus** This condition is associated with increased risk for adverse health events as a result of pregnancy (Box 3).				
a. Positive (or unknown) antiphospholipid antibodies	1	1	1	—
b. Severe thrombocytopenia	1	1	1	—
c. Immunosuppressive therapy	1	1	1	—
d. None of the above	1	1	1	—
**Rheumatoid arthritis**				
a. Not receiving immunosuppressive therapy	1	1	1	—
b. Receiving immunosuppressive therapy	1	1	1	—
**Neurologic Conditions**				
**Headaches**				
a. Nonmigraine (mild or severe)	1	1	1	—
b. Migraine				
i. Without aura (includes menstrual migraine)	1	1	1	**Comment:** Menstrual migraine is a subtype of migraine without aura. For more information see the International Headache Society’s *International Classification of Headache Disorders, 3rd ed.* (https://ichd-3.org) (*4*).
ii. With aura	1	1	1	—
**Epilepsy** This condition is associated with increased risk for adverse health events as a result of pregnancy (Box 3).	1	1	1	—
**Multiple sclerosis**				
a. Without prolonged immobility	1	1	1	—
b. With prolonged immobility	1	1	1	—
**Depressive Disorders**				
**Depressive disorders**	1	1	1	—
**Reproductive Tract Infections and Disorders**				
**Unexplained vaginal bleeding** (suspicious for serious condition) before evaluation	1	1	1	**Clarification:** If pregnancy or an underlying pathological condition (e.g., pelvic malignancy) is suspected, it must be evaluated and the category adjusted after evaluation.
**Endometriosis**	1	1	1	—
**Benign ovarian tumors** (including cysts)	1	1	1	—
**Severe dysmenorrhea**	1	1	1	—
**Gestational trophoblastic disease** This condition is associated with increased risk for adverse health events as a result of pregnancy (Box 3).				
a. Suspected gestational trophoblastic disease (immediate postevacuation)				
i. Uterine size first trimester	1	1	1	—
ii. Uterine size second trimester	1	1	1	—
b. Confirmed gestational trophoblastic disease (after initial evacuation and during monitoring)				
i. Undetectable or nonpregnant β–hCG levels	1	1	1	—
ii. Decreasing β–hCG levels	1	1	1	—
iii. Persistently elevated β-hCG levels or malignant disease, with no evidence or suspicion of intrauterine disease	1	1	1	—
iv. Persistently elevated β-hCG levels or malignant disease, with evidence or suspicion of intrauterine disease	1	1	1	—
**Cervical ectropion**	1	1	1	—
**Cervical intraepithelial neoplasia**	1	1	1	**Clarification:** The cap should not be used. Diaphragm use has no restrictions.
**Cervical cancer** (awaiting treatment)	1	Vaginal pH modulator: 1 Spermicide: 2	1	**Clarification:** The cap should not be used. Diaphragm use has no restrictions. **Comment:** Repeated and high-dose use of the spermicide nonoxynol-9 can cause vaginal and cervical irritation or abrasions.
**Breast disease** Breast cancer is associated with increased risk for adverse health events as a result of pregnancy (Box 3).				
a. Undiagnosed mass	1	1	1	—
b. Benign breast disease	1	1	1	—
c. Family history of cancer	1	1	1	—
d. Breast cancer				
i. Current	1	1	1	—
ii. Past and no evidence of current disease for 5 years	1	1	1	—
**Endometrial hyperplasia**	1	1	1	—
**Endometrial cancer** This condition is associated with increased risk for adverse health events as a result of pregnancy (Box 3).	1	1	1	—
**Ovarian cancer** This condition is associated with increased risk for adverse health events as a result of pregnancy (Box 3).	1	1	1	—
**Uterine fibroids**	1	1	1	—
**Anatomical abnormalities**	1	1	NA	**Clarification:** The diaphragm cannot be used in certain cases of prolapse. Cap use is not appropriate for a person with markedly distorted cervical anatomy.
**Pelvic inflammatory disease**				
a. Current PID	1	1	1	—
b. Past PID				
i. With subsequent pregnancy	1	1	1	—
ii. Without subsequent pregnancy	1	1	1	—
**Sexually transmitted infections**				
a. Current purulent cervicitis or chlamydial infection or gonococcal infection	1	1	1	—
b. Vaginitis (including *Trichomonas vaginalis* and bacterial vaginosis)	1	1	1	—
c. Other factors related to STIs	1	1	1	—
**HIV**				
**High risk for HIV infection**	1	Vaginal pH modulator: 1 Spermicide: 4	4	**Evidence:** Repeated and high-dose use of the spermicide nonoxynol-9 was associated with increased risk for genital lesions, which might increase the risk for HIV infection (*5*). **Comment:** Diaphragm and cap use is assigned category 4 because of concerns about the spermicide, not the diaphragm or cap.
**HIV infection** For persons with HIV infection who are not clinically well or not receiving ARV therapy, this condition is associated with increased risk for adverse health events as a result of pregnancy (Box 3).	1	Vaginal pH modulator: 1 Spermicide: 3	3	**Comment:** Use of spermicides, including with diaphragms and caps, can disrupt the cervical mucosa, which might increase viral shedding and HIV transmission to noninfected sex partners.
**Other Infections**				
**Schistosomiasis** Schistosomiasis with fibrosis of the liver is associated with increased risk for adverse health events as a result of pregnancy (Box 3).				
a. Uncomplicated	1	1	1	—
b. Fibrosis of the liver	1	1	1	—
**Tuberculosis** This condition is associated with increased risk for adverse health events as a result of pregnancy (Box 3).				
a. Nonpelvic	1	1	1	—
b. Pelvic	1	1	1	—
**Malaria**	1	1	1	—
**History of toxic shock syndrome**	1	1	3	**Comment:** Toxic shock syndrome has been reported in association with contraceptive sponge and diaphragm use.
**Urinary tract infection**	1	Vaginal pH modulator: 2 Spermicide: 1	2	**Comment:** Use of diaphragms and spermicides might increase risk for urinary tract infection.
**Endocrine Conditions**				
**Diabetes** Insulin-dependent diabetes; diabetes with nephropathy, retinopathy, or neuropathy; diabetes with other vascular disease; or diabetes of >20 years’ duration are associated with increased risk for adverse health events as a result of pregnancy (Box 3).				
a. History of gestational disease	1	1	1	—
b. Nonvascular disease				
i. Non-insulin dependent	1	1	1	—
ii. Insulin dependent	1	1	1	—
c. Nephropathy, retinopathy, or neuropathy	1	1	1	—
d. Other vascular disease or diabetes of >20 years' duration	1	1	1	—
**Thyroid disorders**				
a. Simple goiter	1	1	1	—
b. Hyperthyroid	1	1	1	—
c. Hypothyroid	1	1	1	—
**Gastrointestinal Conditions**				
**Inflammatory bowel disease** (ulcerative colitis or Crohn’s disease)	1	1	1	—
**Gallbladder disease**				
a. Asymptomatic	1	1	1	—
b. Symptomatic				
i. Current	1	1	1	—
ii. Treated by cholecystectomy	1	1	1	—
iii. Medically treated	1	1	1	—
**History of cholestasis**				
a. Pregnancy related	1	1	1	—
b. Past COC related	1	1	1	—
**Viral hepatitis**				
a. Acute or flare	1	1	1	—
b. Chronic	1	1	1	—
**Cirrhosis** Decompensated cirrhosis is associated with increased risk for adverse health events as a result of pregnancy (Box 3).				
a. Compensated (normal liver function)	1	1	1	—
b. Decompensated (impaired liver function)	1	1	1	—
**Liver tumors** Hepatocellular adenoma and malignant liver tumors are associated with increased risk for adverse health events as a result of pregnancy (Box 3).				
a. Benign				
i. Focal nodular hyperplasia	1	1	1	—
ii. Hepatocellular adenoma	1	1	1	—
b. Malignant (hepatocellular carcinoma)	1	1	1	—
**Respiratory Conditions**				
**Cystic fibrosis** This condition is associated with increased risk for adverse health events as a result of pregnancy (Box 3).	1	1	1	—
**Hematologic Conditions**				
**Thalassemia**	1	1	1	—
**Sickle cell disease** This condition is associated with increased risk for adverse health events as a result of pregnancy (Box 3).	1	1	1	—
**Iron deficiency anemia**	1	1	1	—
**Solid Organ Transplantation**				
**Solid organ transplantation** This condition is associated with increased risk for adverse health events as a result of pregnancy (Box 3).				
a. No graft failure	1	1	1	—
b. Graft failure	1	1	1	—
**Drug Interactions**				
**Antiretrovirals used for prevention (PrEP) or treatment of HIV infection**				**Clarification:** No drug interaction between ARV therapy and barrier method use is known. HIV infection is classified as category 1 for vaginal pH modulator and category 3 for spermicide and diaphragm and cap use (see recommendations for HIV infection). High risk for HIV infection is classified as category 1 for vaginal pH modulator and category 4 for spermicide and diaphragm or cap (see recommendations for High risk for HIV infection).
a. Nucleoside reverse transcriptase inhibitors (NRTIs)				
i. Abacavir (ABC)	1	1/3/4	3/4	
ii. Tenofovir (TDF)	1	1/3/4	3/4	
iii. Zidovudine (AZT)	1	1/3/4	3/4	
iv. Lamivudine (3TC)	1	1/3/4	3/4	
v. Didanosine (DDI)	1	1/3/4	3/4	
vi. Emtricitabine (FTC)	1	1/3/4	3/4	
vii. Stavudine (D4T)	1	1/3/4	3/4	
b. Nonnucleoside reverse transcriptase inhibitors (NNRTIs)				
i. Efavirenz (EFV)	1	1/3/4	3/4	
ii. Etravirine (ETR)	1	1/3/4	3/4	
iii. Nevirapine (NVP)	1	1/3/4	3/4	
iv. Rilpivirine (RPV)	1	1/3/4	3/4	
c. Ritonavir-boosted protease inhibitors				
i. Ritonavir-boosted atazanavir (ATV/r)	1	1/3/4	3/4	
ii. Ritonavir-boosted darunavir (DRV/r)	1	1/3/4	3/4	
iii. Ritonavir-boosted fosamprenavir (FPV/r)	1	1/3/4	3/4	
iv. Ritonavir-boosted lopinavir (LPV/r)	1	1/3/4	3/4	
v. Ritonavir-boosted saquinavir (SQV/r)	1	1/3/4	3/4	
vi. Ritonavir-boosted tipranavir (TPV/r)	1	1/3/4	3/4	
d. Protease inhibitors without ritonavir				
i. Atazanavir (ATV)	1	1/3/4	3/4	
ii. Fosamprenavir (FPV)	1	1/3/4	3/4	
iii. Indinavir (IDV)	1	1/3/4	3/4	
iv. Nelfinavir (NFV)	1	1/3/4	3/4	
e. CCR5 co-receptor antagonists				
i. Maraviroc (MVC)	1	1/3/4	3/4	
f. HIV integrase strand transfer inhibitors				
i. Raltegravir (RAL)	1	1/3/4	3/4	
ii. Dolutegravir (DTG)	1	1/3/4	3/4	
iii. Elvitegravir (EVG)	1	1/3/4	3/4	
g. Fusion inhibitors				
i. Enfuvirtide	1	1/3/4	3/4	
**Anticonvulsant therapy**				
a. Certain anticonvulsants (phenytoin, carbamazepine, barbiturates, primidone, topiramate, or oxcarbazepine)	1	1	1	—
b. Lamotrigine	1	1	1	—
**Antimicrobial therapy**				
a. Broad-spectrum antibiotics	1	1	1	—
b. Antifungals	1	1	1	—
c. Antiparasitics	1	1	1	—
d. Rifampin or rifabutin therapy	1	1	1	—
**Psychotropic medications**				
a. Selective serotonin reuptake inhibitors (SSRIs)	1	1	1	—
**St. John’s wort**	1	1	1	—
**Allergy to latex**	3	1	3	**Clarification:** The condition of allergy to latex does not apply to plastic condoms or diaphragms.

**Abbreviations:** ARV = antiretroviral; BMI = body mass index; COC = combined oral contraceptive; DVT = deep venous thrombosis; hCG = human chorionic gonadotropin; HDL = high-density lipoprotein; LDL = low-density lipoprotein; NA = not applicable; PE = pulmonary embolism; PID = pelvic inflammatory disease; PrEP = pre-exposure prophylaxis; STI = sexually transmitted infection.

## Appendix F Classifications for Fertility Awareness–Based Methods

Fertility awareness–based (FAB) methods involve identifying the fertile days of the menstrual cycle, whether by observing fertility signs, such as cervical secretions and basal body temperature or by monitoring cycle days, and might include use of Food and Drug Administration–cleared contraceptive software applications (Box F1) (Table F1). FAB methods can be used in combination with abstinence or barrier methods during the fertile time. If barrier methods are used, see Classifications for Barrier Methods (Appendix E).

No medical conditions worsen because of FAB methods. In general, FAB methods can be used without concern for health effects in persons who choose them. However, multiple conditions make their use more complex. The existence of these conditions suggests that use of these methods should be delayed until the condition is corrected or resolved, or persons using FAB methods need special counseling; and a provider with particular training in use of these methods is generally necessary to ensure correct use.

FAB methods do not protect against sexually transmitted infections (STIs), including HIV infection, and patients using FAB methods should be counseled that consistent and correct use of external (male) latex condoms reduces the risk for STIs, including HIV infection (*1*). Use of internal (female) condoms can provide protection from transmission of STIs, although data are limited (*1*). Patients also should be counseled that pre-exposure prophylaxis, when taken as prescribed, is highly effective for preventing HIV infection (*2*).

BOX F1 Definitions for terms associated with fertility awareness–based methods

- **Symptoms-based methods:** FAB methods based on observation of fertility signs (e.g., cervical secretions or basal body temperature) such as the cervical mucus method, the symptothermal method, and the TwoDay method.
- **Calendar-based methods:** FAB methods based on calendar calculations such as the calendar rhythm method and the standard days method.
- **Accept:** No medical reason exists to deny the particular FAB method to a patient in this circumstance.
- **Caution:** The method normally is provided in a routine setting but with extra preparation and precautions. For FAB methods, this usually means that special counseling might be needed to ensure correct use of the method by a patient in this circumstance.
- **Delay:** Use of this method should be delayed until the condition is evaluated or corrected. Alternative temporary methods of contraception should be offered.

**Abbreviation:** FAB = fertility awareness–based.

**TABLE F1:** Fertility awareness–based methods, including symptoms-based and calendar-based methods

Condition	Category			Clarification/Evidence/Comment
	Symptoms-based method	Calendar-based method		
**Personal Characteristics and Reproductive History**				
**Pregnancy**	NA		NA	**Clarification:** FAB methods are not relevant during pregnancy.
**Life stage**				**Comment:** Menstrual irregularities are common in postmenarche and perimenopause and might complicate the use of FAB methods.
a. Postmenarche	Caution		Caution	
b. Perimenopause	Caution		Caution	
**Breastfeeding**				**Comment:** Use of FAB methods when breastfeeding might be less effective than when not breastfeeding.
a. <6 weeks postpartum	Delay		Delay	**Comment:** Persons who are primarily breastfeeding and are amenorrheic are unlikely to have sufficient ovarian function to produce detectable fertility signs and hormonal changes during the first 6 months postpartum. However, the likelihood of resumption of fertility increases with time postpartum and with substitution of breast milk by other foods.
b. ≥6 weeks postpartum	Caution		Delay	
c. After menses begin	Caution		Caution	**Clarification:** Once fertility signs are noted, particularly cervical secretions, then symptoms-based methods can be used. First postpartum menstrual cycles while breastfeeding vary significantly in length. Return to regularity takes several cycles. When there have been at least three postpartum menses and cycles are regular again, a calendar-based method can be used. When there have been at least four postpartum menses and the most recent cycle lasted 26–32 days, the standard days method can be used. Before that time, a barrier method should be offered if the patient plans to use a FAB method later.
**Postpartum** (nonbreastfeeding)				
a. <4 weeks	Delay		Delay	**Clarification:** Nonbreastfeeding persons are not likely to have detectable fertility signs or hormonal changes before 4 weeks postpartum. Although the risk for pregnancy is low, ovulation before first menses is common; therefore, a method appropriate for the postpartum period should be offered.
b. ≥4 weeks	Accept		Delay	**Clarification:** Nonbreastfeeding persons are likely to have sufficient ovarian function to produce detectable fertility signs, hormonal changes, or both at this time; likelihood increases rapidly with time postpartum. Calendar-based methods can be used as soon as three postpartum menses have been completed. Methods appropriate for the postpartum period should be offered before that time.
**Postabortion **(spontaneous or induced)	Caution		Delay	**Clarification:** After abortion, it is possible for ovarian function to produce detectable fertility signs, hormonal changes, or both; likelihood increases with time postabortion. Calendar-based methods can be used following at least one postabortion menses (e.g., persons who before this pregnancy primarily had cycles of 26–32 days can then use the standard days method). Methods appropriate for the postabortion period should be offered before that time.
**Reproductive Tract Infections and Disorders**				
**Irregular vaginal bleeding**	Delay		Delay	**Clarification:** Presence of this condition makes FAB methods unreliable. Therefore, barrier methods should be recommended until the bleeding pattern is compatible with proper method use. The condition should be evaluated and treated as necessary.
**Vaginal discharge**	Delay		Accept	**Clarification:** Because vaginal discharge makes recognition of cervical secretions difficult, the condition should be evaluated and treated if needed before providing methods based on cervical secretions.
**Other**				
**Use of drugs that affect cycle regularity, hormones, or fertility signs**	Caution /Delay		Caution/Delay	**Clarification:** Use of certain mood-altering drugs (e.g., lithium, tricyclic antidepressants, and antianxiety therapies), as well as certain antibiotics and anti-inflammatory drugs, might alter cycle regularity or affect fertility signs. The condition should be carefully evaluated and a barrier method offered until the degree of effect has been determined or the drug is no longer being used.
**Diseases that elevate body temperature**				
a. Chronic diseases	Caution		Accept	**Clarification:** Elevated temperatures might make basal body temperature difficult to interpret but have no effect on cervical secretions. Thus, use of a method that relies on temperature should be delayed until the acute febrile disease abates. Temperature-based methods are not appropriate for persons with chronically elevated temperatures. In addition, certain chronic diseases interfere with cycle regularity, making calendar-based methods difficult to interpret.
b. Acute diseases	Delay		Accept	

**Abbreviations:** FAB = fertility awareness–based; NA = not applicable.

## Appendix G Lactational Amenorrhea Method

The Bellagio Consensus provided the scientific basis for defining the conditions under which breastfeeding can be used safely and effectively for birth-spacing purposes; programmatic guidelines were developed at a meeting of family planning experts for its use as a method of contraception, and the method was then named the lactational amenorrhea method (LAM) (*1*–*3*). These guidelines include the following three criteria, all of which must be met to ensure adequate protection from pregnancy: 1) amenorrhea, 2) fully or nearly fully breastfeeding (intervals between feedings not exceeding 4 hours during the day or 6 hours at night), and 3) <6 months postpartum (*4*–*6*).

The U.S. Dietary Guidelines for Americans recommend that infants be exclusively breastfed for about the first 6 months, with continued breastfeeding while introducing appropriate complementary foods for 1 year or longer (*7*). The American Academy of Pediatrics (AAP) recommends exclusive breastfeeding for about the first 6 months, with continued breastfeeding along with introducing appropriate complementary foods for up to age 2 years or longer (*8*).

No medical conditions exist for which use of LAM for contraception is restricted. However, breastfeeding might not be recommended for persons or infants with certain conditions.

LAM does not protect against sexually transmitted infections (STIs), including HIV infection, and patients using LAM should be counseled that consistent and correct use of external (male) latex condoms reduces the risk for STIs, including HIV infection (*9*). Use of internal (female) condoms can provide protection from transmission of STIs, although data are limited (*9*). Patients also should be counseled that pre-exposure prophylaxis, when taken as prescribed, is highly effective for preventing HIV infection (*10*).

**HIV Infection.** HIV transmission can occur during breastfeeding. For breastfeeding persons on antiretroviral therapy with a sustained undetectable HIV viral load during pregnancy, the risk for transmission through breastfeeding is <1%, but not zero. Patients with HIV infection should receive evidence-based, person-centered counseling to support shared decision-making about infant feeding. For comprehensive information, refer to Infant Feeding for Individuals with HIV in the United States (https://clinicalinfo.hiv.gov/en/guidelines/perinatal/counseling-and-managing-individuals-with-hiv-united-states-who-desire-breastfeed). These recommendations are included within the U.S. Department of Health and Human Services’s Recommendations for the Use of Antiretroviral Drugs During Pregnancy and Interventions to Reduce Perinatal HIV Transmission in the United States (https://clinicalinfo.hiv.gov/en/guidelines/perinatal/whats-new-guidelines) (*11*).

**Other Medical Conditions.** CDC and AAP also recommend against both breastfeeding and feeding expressed milk for persons with untreated brucellosis, positivity for human T-cell lymphotropic virus types I or II, herpes simplex lesions on a breast, Ebola virus disease, or mpox. In addition, infants with classic galactosemia should not breastfeed (*8*,*12*,*13*) (https://www.cdc.gov/breastfeeding-special-circumstances/hcp/contraindications/index.html).

**Medication Used During Breastfeeding.** Although many medications do pass into breast milk, most have little or no effect on milk supply or on infant well-being. Few medications are contraindicated while breastfeeding. More information about specific medications and radioactive compounds is provided by AAP (*14*), LactMed (https://www.ncbi.nlm.nih.gov/books/NBK501922), Mother to Baby (http://www.mothertobaby.org), and InfantRisk Center (https://www.infantrisk.com/category/breastfeeding).

## Appendix H Coitus Interruptus (Withdrawal)

Coitus interruptus, also known as withdrawal, is a contraceptive method in which the penis is completely removed from the vagina and away from the external genitalia before ejaculation. Coitus interruptus prevents sperm from entering the vagina, thereby preventing contact between spermatozoa and the ovum.

Coitus interruptus has no directly associated health risks. Coitus interruptus does not protect against sexually transmitted infections (STIs), including HIV infection, and patients using coitus interruptus should be counseled that consistent and correct use of external (male) latex condoms reduces the risk for STIs, including HIV infection (*1*). Use of internal (female) condoms can provide protection from transmission of STIs, although data are limited (*1*). Patients also should be counseled that pre-exposure prophylaxis, when taken as prescribed, is highly effective for preventing HIV infection (*2*).

## Appendix I Permanent Contraception

Tubal surgery (including laparoscopic and abdominal approaches) and vasectomy are methods of permanent contraception available in the United States. In general, no medical conditions absolutely restrict a person’s eligibility for permanent contraception (with the exception of known allergy or hypersensitivity to any materials used to complete the permanent contraception procedure). However, certain conditions might increase a person’s surgical risk during tubal surgery; in these cases, careful consideration can be given to the risks and benefits of other acceptable long-acting or permanent alternatives, including intrauterine device, implant, and vasectomy.

Patients should be appropriately counseled that permanent contraception is intended to be irreversible and about the availability of highly effective, long-acting reversible methods of contraception. Most persons who choose permanent contraception remain satisfied with their decision. However, a small proportion of women regret this decision (1%–26% from different studies, with higher rates of regret reported by women who were younger at time of permanent contraception procedure) (*1*,*2*). Regret among men about vasectomy has been reported to be approximately 5% (*3*), similar to the proportion of women who report regretting their husbands’ vasectomy (6%) (*4*).

Permanent contraception does not protect against sexually transmitted infections (STIs), including HIV infection, and patients using permanent contraception should be counseled that consistent and correct use of external (male) latex condoms reduces the risk for STIs, including HIV infection (*5*). Use of internal (female) condoms can provide protection from transmission of STIs, although data are limited (*5*). Patients also should be counseled that pre-exposure prophylaxis, when taken as prescribed, is highly effective for preventing HIV infection (*6*).

## Appendix J: Classifications for Emergency Contraception

Classifications are given for the copper intrauterine device (Cu-IUD) as emergency contraception. The Cu-IUD can be placed within 5 days of the first act of unprotected intercourse as emergency contraception. In addition, when the day of ovulation can be estimated, the Cu-IUD can be placed beyond 5 days after sexual intercourse, as long as the placement does not occur >5 days after ovulation. The eligibility criteria for interval Cu-IUD placement also apply for the placement of Cu-IUDs as emergency contraception (Box J1) (Table J1) (*1*).

Classifications for emergency contraceptive pills (ECPs) are given for ulipristal acetate (UPA), levonorgestrel (LNG), and combined oral contraceptives (COCs). ECPs should be taken as soon as possible within 5 days of unprotected sexual intercourse (*1*).

Cu-IUDs, UPA, LNG, and COCs do not protect against sexually transmitted infections (STIs), including HIV infection, and patients using these methods should be counseled that consistent and correct use of external (male) latex condoms reduces the risk for STIs, including HIV infection (*2*). Use of internal (female) condoms can provide protection from transmission of STIs, although data are limited (*2*). Patients also should be counseled that pre-exposure prophylaxis, when taken as prescribed, is highly effective for preventing HIV infection (*3*).

BOX J1. Categories for classifying emergency contraception U.S. MEC 1 = A condition for which there is no restriction for the use of the contraceptive method. U.S. MEC 2 = A condition for which the advantages of using the method generally outweigh the theoretical or proven risks. U.S. MEC 3 = A condition for which the theoretical or proven risks usually outweigh the advantages of using the method. U.S. MEC 4 = A condition that represents an unacceptable health risk if the contraceptive method is used. **Abbreviation:** *U.S. Medical Eligibility Criteria for Contraceptive Use*.

**TABLE:** J1. Classifications for emergency contraception, including the copper intrauterine device, ulipristal acetate, levonorgestrel, and combined oral contraceptives

Condition	Category				Clarification/Evidence/Comment
	Cu-IUD	UPA	LNG	COC	
**Personal Characteristics and Reproductive History**					
**Pregnancy**	4	NA	NA	NA	**Clarification (IUD):** The IUD is not indicated during pregnancy and should not be used because of the risk for serious pelvic infection and septic spontaneous abortion. **Clarification** (**ECPs):** Although this method is not indicated for a patient with a known or suspected pregnancy, no harm to the patient, the course of pregnancy, or the fetus if ECPs are inadvertently used is known to exist. **Evidence:** Evidence suggests that poor pregnancy outcomes are rare among pregnant women who used ECPs during conception cycle or early in pregnancy (*4*).
**Breastfeeding**	1	1	1	1	**Evidence:** Breastfeeding outcomes do not seem to differ between women exposed to LNG and those who are not exposed (*4*). One pharmacokinetic study demonstrated that LNG passes to breast milk but in minimal quantities (*4*). UPA and its active metabolite, monodemethyl-ulipristal acetate, are present in human milk in small amounts; no evidence is available on effects of UPA emergency contraception exposure on infants or children who are breastfed (*5*).
**Past ectopic pregnancy**	1	1	1	1	—
**Obesity (BMI ≥30 kg/m^2^)**	1	2	2	2	**Clarification** **(ECPs):** ECPs might be less effective among persons with BMI ≥30 kg/m^2^ than among persons with BMI <25 kg/m^2^. Despite this, no safety concerns exist. **Evidence:** Limited evidence from secondary data analyses suggests that women with BMI ≥30 kg/m^2^ experience an increased risk for pregnancy after use of LNG compared with women with BMI <25 kg/m^2^. Two analyses suggest that women with obesity might also experience an increased risk for pregnancy after use of UPA compared with those without obesity, although this increase was not significant in one study (*6*).
**History of bariatric surgery** This condition is associated with increased risk for adverse health events as a result of pregnancy (Box 3).					
a. Restrictive procedures: decrease storage capacity of the stomach (vertical banded gastroplasty, laparoscopic adjustable gastric band, or laparoscopic sleeve gastrectomy)	1	1	1	1	—
b. Malabsorptive procedures: decrease absorption of nutrients and calories by shortening the functional length of the small intestine (Roux-en-Y gastric bypass or biliopancreatic diversion)	1	1	1	1	**Comment:** Bariatric surgical procedures involving a malabsorptive component have the potential to decrease oral contraceptive effectiveness, perhaps further decreased by postoperative complications such as long-term diarrhea, vomiting, or both. Because of these malabsorptive concerns, an emergency IUD might be more appropriate than ECPs.
**Cardiovascular Disease**					
**History of severe cardiovascular disease** (ischemic heart disease, cerebrovascular attack, or other thromboembolic conditions) This condition is associated with increased risk for adverse health events as a result of pregnancy (Box 3).	1	2	2	2	**Comment:** The duration of ECP use is less than that of regular use of COCs or POPs and thus would be expected to have less clinical impact.
**Rheumatic Diseases**					
**Rheumatoid arthritis**					
a. Not receiving immunosuppressive therapy	1	1	1	1	—
b. Receiving immunosuppressive therapy	2	1	1	1	—
**Neurologic Conditions**					
**Migraine**	1	1	1	2	**Comment:** The duration of ECP use is less than that of regular use of COCs and thus would be expected to have less clinical impact.
**Gastrointestinal Conditions**					
**Inflammatory bowel disease** (ulcerative colitis or Crohn’s disease)	1	1	1	1	—
**Severe liver disease** (including jaundice) This condition is associated with increased risk for adverse health events as a result of pregnancy (Box 3).	1	2	2	2	**Comment:** The duration of ECP use is less than that of regular use of COCs or POPs and thus would be expected to have less clinical impact.
**Solid Organ Transplantation**					
**Solid organ transplantation** This condition is associated with increased risk for adverse health events as a result of pregnancy (Box 3).					
a. No graft failure	1	1	1	1	—
b. Graft failure	2	1	1	1	—
**Other**					
**Repeated ECP use**	—	1	1	1	**Clarification (ECPs):** Frequently repeated ECP use might be harmful for persons with conditions classified as category 2, 3, or 4 for CHC or POC use. **Evidence:** In one case-control study, risk for ectopic pregnancy compared with intrauterine pregnancy did not increase after repeated use of LNG ECPs compared with nonuse (*4*).
**Sexual assault**	2	1	1	1	**Clarification (IUD):** Persons who have experienced sexual assault are at increased risk for STIs, including HIV infection. According to CDC STI treatment guidelines, routine presumptive treatment of chlamydia, gonorrhea, and trichomonas is recommended after sexual assault (*2*). Persons with current purulent cervicitis, chlamydial infection, or gonococcal infection should not undergo IUD placement (category 4).
**CYP3A4 inducers** (e.g., bosentan, carbamazepine, felbamate, griseofulvin, oxcarbazepine, phenytoin, rifampin, St. John’s wort, topiramate, efavirenz, and lumacaftor)	1	2	2	2	**Clarification (ECPs):** Strong CYP3A4 inducers might reduce the effectiveness of ECPs. **Evidence:** According to labelling information, rifampin markedly decreases UPA levels by ≥90%, which might decrease its efficacy (*5*). Therefore, theoretical concerns extend to use of other CYP3A4 inducers as well as to COC and LNG ECPs, which have metabolic pathways similar to those of UPA. A small pharmacokinetic study found that concomitant efavirenz use decreased LNG levels in women taking LNG ECPs (1.5 mg) by 56% compared with LNG ECPs alone (*7*).

**Abbreviations:** BMI = body mass index; CHC = combined hormonal contraceptive; COC = combined hormonal contraceptive; Cu-IUD = copper intrauterine device; CYP = cytochrome P450; ECP = emergency contraceptive pill; IUD = intrauterine device; LNG = levonorgestrel; NA = not applicable; POC = progestin-only contraceptive; POP = progestin-only pill; STI = sexually transmitted infection; UPA = ulipristal acetate.

## Appendix K: Summary of Classifications for Hormonal Contraceptive Methods and Intrauterine Devices

Health care providers can use the summary table as a quick reference guide to the classifications for hormonal contraceptive methods and intrauterine contraception to compare classifications across these methods (Box K1) (Table K1). See the respective appendix for each method for clarifications to the numeric categories, as well as for summaries of the evidence and additional comments. Hormonal contraceptives and intrauterine devices do not protect against sexually transmitted infections (STIs), including HIV infection, and patients using these methods should be counseled that consistent and correct use of external (male) latex condoms reduces the risk for STIs, including HIV infection (*1*). Use of internal (female) condoms can provide protection from transmission of STIs, although data are limited (*1*). Patients also should be counseled that pre-exposure prophylaxis, when taken as prescribed, is highly effective for preventing HIV infection (*2*).

BOX K1. Categories for classifying hormonal contraceptives and intrauterine devices U.S. MEC 1 = A condition for which there is no restriction for the use of the contraceptive method U.S. MEC 2 = A condition for which the advantages of using the method generally outweigh the theoretical or proven risks U.S. MEC 3 = A condition for which the theoretical or proven risks usually outweigh the advantages of using the method U.S. MEC 4 = A condition that represents an unacceptable health risk if the contraceptive method is used **Abbreviation:** U.S. MEC = *U.S. Medical Eligibility Criteria*.

**TABLE:** K1. Summary of classifications for hormonal contraceptive methods and intrauterine devices

Condition	Cu-IUD		LNG-IUD		Implant		DMPA		POP		CHC	
**Personal Characteristics and Reproductive History**												
**Pregnancy**	4*		4*		NA*		NA*		NA*		NA*	
**Age**	Menarche to <20 years: 2		Menarche to <20 years: 2		Menarche to <18 years: 1		Menarche to <18 years: 2		Menarche to <18 years: 1		Menarche to <40 years: 1	
	≥20 years: 1		≥20 years: 1		18–45 years: 1		18–45 years: 1		18–45 years: 1		≥40 years: 2	
					>45 years: 1		>45 years: 2		>45 years: 1			
**Parity**												
a. Nulliparous	2		2		1		1		1		1	
b. Parous	1		1		1		1		1		1	
**Breastfeeding**												
a. <21 days postpartum	—		—		2*		2*		2*		4*	
b. 21 to <30 days postpartum												
i. With other risk factors for VTE (e.g., age ≥35 years, previous VTE, thrombophilia, immobility, transfusion at delivery, peripartum cardiomyopathy, BMI ≥30 kg/m^2^, postpartum hemorrhage, postcesarean delivery, preeclampsia, or smoking)	—		—		2*		2*		2*		3*	
ii. Without other risk factors for VTE	—		—		2*		2*		2*		3*	
c. 30–42 days postpartum												
i. With other risk factors for VTE (e.g., age ≥35 years, previous VTE, thrombophilia, immobility, transfusion at delivery, peripartum cardiomyopathy, BMI ≥30 kg/m^2^, postpartum hemorrhage, postcesarean delivery, preeclampsia, or smoking)	—		—		1*		2*		1*		3*	
ii. Without other risk factors for VTE	—		—		1*		1*		1*		2*	
d. >42 days postpartum	—		—		1*		1*		1*		2*	
**Postpartum** (nonbreastfeeding)												
a. <21 days postpartum	—		—		1		2		1		4	
b. 21–42 days postpartum												
i. With other risk factors for VTE (e.g., age ≥35 years, previous VTE, thrombophilia, immobility, transfusion at delivery, peripartum cardiomyopathy, BMI ≥30 kg/m^2^, postpartum hemorrhage, postcesarean delivery, preeclampsia, or smoking)	—		—		1		2		1		3*	
ii. Without other risk factors for VTE	—		—		1		1		1		2	
c. >42 days postpartum	—		—		1		1		1		1	
**Postpartum** (including cesarean delivery, breastfeeding, or nonbreastfeeding)												
a. <10 minutes after delivery of the placenta	2*		2*		—		—		—		—	
b. 10 minutes after delivery of the placenta to <4 weeks	2*		2*		—		—		—		—	
c. ≥4 weeks	1*		1*		—		—		—		—	
d. Postpartum sepsis	4		4		—		—		—		—	
**Postabortion **(spontaneous or induced)												
a. First trimester abortion												
i. Procedural (surgical)	1*		1*		1*		1*		1*		1*	
ii. Medication	1*		1*		1*		1/2*		1*		1*	
iii. Spontaneous abortion with no intervention	1*		1*		1*		1*		1*		1*	
b. Second trimester abortion												
i. Procedural (surgical)	2*		2*		1*		1*		1*		1*	
ii. Medication	2*		2*		1*		1*		1*		1*	
iii. Spontaneous abortion with no intervention	2*		2*		1*		1*		1*		1*	
c. Immediate postseptic abortion	4		4		1*		1*		1*		1*	
**Past ectopic pregnancy**	1		1		1		1		2		1	
**History of pelvic surgery** (see recommendations for Postpartum [including cesarean delivery])	1		1		1		1		1		1	
**Smoking**												
a. Age <35 years	1		1		1		1		1		2	
b. Age ≥35 years												
i. <15 cigarettes per day	1		1		1		1		1		3	
ii. ≥15 cigarettes per day	1		1		1		1		1		4	
**Obesity**												
a. BMI ≥30 kg/m^2^	1		1		1		1		1		2*	
b. Menarche to <18 years and BMI ≥30 kg/m^2^	1		1		1		2		1		2*	
**History of bariatric surgery** This condition is associated with increased risk for adverse health events as a result of pregnancy (Box 3).												
a. Restrictive procedures: decrease storage capacity of the stomach (vertical banded gastroplasty, laparoscopic adjustable gastric band, or laparoscopic sleeve gastrectomy)	1		1		1		1		1		1	
b. Malabsorptive procedures: decrease absorption of nutrients and calories by shortening the functional length of the small intestine (Roux-en-Y gastric bypass or biliopancreatic diversion)	1		1		1		1		3		COCs: 3 Patch and ring: 1	
**Surgery**												
a. Minor surgery without immobilization	1		1		1		1		1		1	
b. Major surgery												
i. Without prolonged immobilization	1		1		1		1		1		2	
ii. With prolonged immobilization	1		1		1		2		1		4	
**Cardiovascular Disease**												
**Multiple risk factors for atherosclerotic cardiovascular disease** (e.g., older age, smoking, diabetes, hypertension, low HDL, high LDL, or high triglyceride levels)	1		2		2*		3*		2*		3/4*	
**Hypertension** Systolic blood pressure ≥160 mm Hg or diastolic blood pressure ≥100 mm Hg are associated with increased risk for adverse health events as a result of pregnancy (Box 3).												
a. Adequately controlled hypertension	1*		1*		1*		2*		1*		3*	
b. Elevated blood pressure levels (properly taken measurements)												
i. Systolic 140–159 mm Hg or diastolic 90–99 mm Hg	1*		1*		1*		2*		1*		3*	
ii. Systolic ≥160 mm Hg or diastolic ≥100 mm Hg	1*		2*		2*		3*		2*		4*	
c. Vascular disease	1*		2*		2*		3*		2*		4*	
**History of high blood pressure during pregnancy** (when current blood pressure is measurable and normal)	1		1		1		1		1		2	
**Deep venous thrombosis/Pulmonary embolism** This condition is associated with increased risk for adverse health events as a result of pregnancy (Box 3).												
a. Current or history of DVT/PE, receiving anticoagulant therapy (therapeutic dose) (e.g., acute DVT/PE or long-term therapeutic dose)	2*		2*		2*		2*		2*		3*	
b. History of DVT/PE, receiving anticoagulant therapy (prophylactic dose)												
i. Higher risk for recurrent DVT/PE (one or more risk factors)	2*		2*		2*		3*		2*		4*	
• Thrombophilia (e.g., factor V Leiden mutation; prothrombin gene mutation; protein S, protein C, and antithrombin deficiencies; or antiphospholipid syndrome) • Active cancer (metastatic, receiving therapy, or within 6 months after clinical remission), excluding nonmelanoma skin cancer • History of recurrent DVT/PE												
ii. Lower risk for recurrent DVT/PE (no risk factors)	2*		2*		2*		2*		2*		3*	
c. History of DVT/PE, not receiving anticoagulant therapy												
i. Higher risk for recurrent DVT/PE (one or more risk factors	1		2		2		3		2		4	
• History of estrogen-associated DVT/PE • Pregnancy-associated DVT/PE • Idiopathic DVT/PE • Thrombophilia (e.g., factor V Leiden mutation; prothrombin gene mutation; protein S, protein C, and antithrombin deficiencies; or antiphospholipid syndrome) • Active cancer (metastatic, receiving therapy, or within 6 months after clinical remission), excluding nonmelanoma skin cancer • History of recurrent DVT/PE												
ii. Lower risk for recurrent DVT/PE (no risk factors)	1		2		2		2		2		3	
d. Family history (first-degree relatives)	1		1		1		1		1		2	
**Thrombophilia** (e.g., factor V Leiden mutation; prothrombin gene mutation; protein S, protein C, and antithrombin deficiencies; or antiphospholipid syndrome) This condition is associated with increased risk for adverse health events as a result of pregnancy (Box 3).	1*		2*		2*		3*		2*		4*	
**Superficial venous disorders**												
a. Varicose veins	1		1		1		1		1		1	
b. Superficial venous thrombosis (acute or history)	1		1		1		2		1		3*	
**Current and history of ischemic heart disease** This condition is associated with increased risk for adverse health events as a result of pregnancy (Box 3).	1		Initiation	Continuation	Initiation	Continuation	3		Initiation	Continuation	4	
			2	3	2	3			2	3		
**Stroke** (history of cerebrovascular accident) This condition is associated with increased risk for adverse health events as a result of pregnancy (Box 3).	1		2		Initiation	Continuation	3		Initiation	Continuation	4	
					2	3			2	3		
**Valvular heart disease** Complicated valvular heart disease is associated with increased risk for adverse health events as a result of pregnancy (Box 3).												
a. Uncomplicated	1		1		1		1		1		2	
b. Complicated (pulmonary hypertension, risk for atrial fibrillation, or history of subacute bacterial endocarditis)	1		1		1		2		1		4	
**Peripartum cardiomyopathy** This condition is associated with increased risk for adverse health events as a result of pregnancy (Box 3).												
a. Normal or mildly impaired cardiac function (New York Heart Association Functional Class I or II: no limitation of activities or slight, mild limitation of activity) (*3*)												
i. <6 months	2		2		1		2		1		4	
ii. ≥6 months	2		2		1		2		1		3	
b. Moderately or severely impaired cardiac function (New York Heart Association Functional Class III or IV: marked limitation of activity or should be at complete rest) (*3*)	2		2		2		3		2		4	
**Renal Disease**												
**Chronic kidney disease** This condition is associated with increased risk for adverse health events as a result of pregnancy (Box 3).	Initiation	Continuation	Initiation	Continuation	—							
a. Current nephrotic syndrome	1	1	2	2	2		3		2* DRSP POP with known hyperkalemia: 4*		4	
b. Hemodialysis	1	1	2	2	2		3		2* DRSP POP with known hyperkalemia: 4*		4	
c. Peritoneal dialysis	2	1	2	2	2		3		2* DRSP POP with known hyperkalemia: 4*		4	
**Rheumatic Diseases**												
**Systemic lupus erythematosus** This condition is associated with increased risk for adverse health events as a result of pregnancy (Box 3).	Initiation	Continuation	—				Initiation	Continuation	—			
a. Positive (or unknown) antiphospholipid antibodies	1*	1*	2*		2*		3*	3*	2*		4*	
b. Severe thrombocytopenia	3*	2*	2*		2*		3*	2*	2*		2*	
c. Immunosuppressive therapy	2*	1*	2*		2*		2*	2*	2*		2*	
d. None of the above	1*	1*	2*		2*		2*	2*	2*		2*	
**Rheumatoid arthritis**	Initiation	Continuation	Initiation	Continuation	—							
a. Not receiving immunosuppressive therapy	1	1	1	1	1		2		1		2	
b. Receiving immunosuppressive therapy	2	1	2	1	1		2/3*		1		2	
**Neurologic Conditions**												
**Headaches**												
a. Nonmigraine (mild or severe)	1		1		1		1		1		1*	
b. Migraine												
i. Without aura (includes menstrual migraine)	1		1		1		1		1		2*	
ii. With aura	1		1		1		1		1		4*	
**Epilepsy** This condition is associated with increased risk for adverse health events as a result of pregnancy (Box 3).	1		1		1*		1*		1*		1*	
**Multiple sclerosis**												
a. Without prolonged immobility	1		1		1		2		1		1	
b. With prolonged immobility	1		1		1		2		1		3	
**Depressive Disorders**												
**Depressive disorders**	1*		1*		1*		1*		1*		1*	
**Reproductive Tract Infections and Disorders**												
**Vaginal bleeding patterns**			Initiation	Continuation	—							
a. Irregular pattern without heavy bleeding	1		1	1	2		2		2		1	
b. Heavy or prolonged bleeding (includes regular and irregular patterns)	2*		1*	2*	2*		2*		2*		1*	
**Unexplained vaginal bleeding** (suspicious for serious condition) before evaluation	Initiation	Continuation	Initiation	Continuation	—							
	4*	2*	4*	2*	3*		3*		2*		2*	
**Endometriosis**	2		1		1		1		1		1	
**Benign ovarian tumors** (including cysts)	1		1		1		1		1		1	
**Severe dysmenorrhea**	2		1		1		1		1		1	
**Gestational trophoblastic disease** This condition is associated with increased risk for adverse health events as a result of pregnancy (Box 3).												
a. Suspected gestational trophoblastic disease (immediate postevacuation)												
i. Uterine size first trimester	1*		1*		1*		1*		1*		1*	
ii. Uterine size second trimester	2*		2*		1*		1*		1*		1*	
b. Confirmed gestational trophoblastic disease (after initial evacuation and during monitoring)	Initiation	Continuation	Initiation	Continuation	—							
i. Undetectable or nonpregnant β-hCG levels	1*	1*	1*	1*	1*		1*		1*		1*	
ii. Decreasing β-hCG levels	2*	1*	2*	1*	1*		1*		1*		1*	
iii. Persistently elevated β-hCG levels or malignant disease, with no evidence or suspicion of intrauterine disease	2*	1*	2*	1*	1*		1*		1*		1*	
iv. Persistently elevated β-hCG levels or malignant disease, with evidence or suspicion of intrauterine disease	4*	2*	4*	2*	1*		1*		1*		1*	
**Cervical ectropion**	1		1		1		1		1		1	
**Cervical intraepithelial neoplasia**	1		2		2		2		1		2	
**Cervical cancer** (awaiting treatment)	Initiation	Continuation	Initiation	Continuation	—							
	4	2	4	2	2		2		1		2	
**Breast disease** Breast cancer is associated with increased risk for adverse health events as a result of pregnancy (Box 3).												
a. Undiagnosed mass	1		2*		2*		2*		2*		2*	
b. Benign breast disease	1		1		1		1		1		1	
c. Family history of cancer	1		1		1		1		1		1	
d. Breast cancer												
i. Current	1		4		4		4		4		4	
ii. Past and no evidence of current disease for 5 years	1		3		3		3		3		3	
**Endometrial hyperplasia**	1		1		1		1		1		1	
**Endometrial cancer** This condition is associated with increased risk for adverse health events as a result of pregnancy (Box 3).	Initiation	Continuation	Initiation	Continuation	—							
	4	2	4	2	1		1		1		1	
**Ovarian cancer** This condition is associated with increased risk for adverse health events as a result of pregnancy (Box 3).	1		1		1		1		1		1	
**Uterine fibroids**	2		2		1		1		1		1	
**Anatomical abnormalities**												
a. Distorted uterine cavity (any congenital or acquired uterine abnormality distorting the uterine cavity in a manner that is incompatible with IUD placement)	4		4		—							
b. Other abnormalities (including cervical stenosis or cervical lacerations) not distorting the uterine cavity or interfering with IUD placement	2		2									
**Pelvic inflammatory disease**	Initiation	Continuation	Initiation	Continuation	—							
a. Current PID	4	2*	4	2*	1		1		1		1	
b. Past PID												
i. With subsequent pregnancy	1	1	1	1	1		1		1		1	
ii. Without subsequent pregnancy	2	2	2	2	1		1		1		1	
**Sexually transmitted infections**	Initiation	Continuation	Initiation	Continuation	—							
a. Current purulent cervicitis or chlamydial infection or gonococcal infection	4	2*	4	2*	1		1		1		1	
b. Vaginitis (including *Trichomonas vaginalis* and bacterial vaginosis)	2	2	2	2	1		1		1		1	
c. Other factors related to STIs	2*	2	2*	2	1		1		1		1	
**HIV**												
**High risk for HIV infection**	Initiation	Continuation	Initiation	Continuation	—							
	1*	1*	1*	1*	1		1		1		1	
**HIV infection** For persons with HIV infection who are not clinically well or not receiving ARV therapy, this condition is associated with increased risk for adverse health events as a result of pregnancy (Box 3).	—	—	—	—	1*		1*		1*		1*	
a. Clinically well receiving ARV therapy	1	1	1	1	—		—		—		—	
b. Not clinically well or not receiving ARV therapy	2	1	2	1	—		—		—		—	
**Other Infections**												
**Schistosomiasis** Schistosomiasis with fibrosis of the liver is associated with increased risk for adverse health events as a result of pregnancy (Box 3).												
a. Uncomplicated	1		1		1		1		1		1	
b. Fibrosis of the liver (if severe, see recommendations for Cirrhosis)	1		1		1		1		1		1	
**Tuberculosis** This condition is associated with increased risk for adverse health events as a result of pregnancy (Box 3).	Initiation	Continuation	Initiation	Continuation	—							
a. Nonpelvic	1	1	1	1	1*		1*		1*		1*	
b. Pelvic	4	3	4	3	1*		1*		1*		1*	
**Malaria**	1		1		1		1		1		1	
**Endocrine Conditions**												
**Diabetes** Insulin-dependent diabetes; diabetes with nephropathy, retinopathy, or neuropathy; diabetes with other vascular disease; or diabetes of >20 years’ duration are associated with increased risk for adverse health events as a result of pregnancy (Box 3).												
a. History of gestational disease	1		1		1		1		1		1	
b. Nonvascular disease												
i. Non-insulin dependent	1		2		2		2		2		2	
ii. Insulin dependent	1		2		2		2		2		2	
c. Nephropathy, retinopathy, or neuropathy	1		2		2		3		2		3/4*	
d. Other vascular disease or diabetes of >20 years’ duration	1		2		2		3		2		3/4*	
**Thyroid disorders**												
a. Simple goiter	1		1		1		1		1		1	
b. Hyperthyroid	1		1		1		1		1		1	
c. Hypothyroid	1		1		1		1		1		1	
**Gastrointestinal Conditions**												
**Inflammatory bowel disease** (ulcerative colitis or Crohn’s disease)	1		1		1		2		2		2/3*	
**Gallbladder disease**												
a. Asymptomatic	1		2		2		2		2		2	
b. Symptomatic												
i. Current	1		2		2		2		2		3	
ii. Treated by cholecystectomy	1		2		2		2		2		2	
iii. Medically treated	1		2		2		2		2		3	
**History of cholestasis**												
a. Pregnancy related	1		1		1		1		1		2	
b. Past COC related	1		2		2		2		2		3	
**Viral hepatitis**											Initiation	Continuation
a. Acute or flare	1		1		1		1		1		3/4*	2
b. Chronic	1		1		1		1		1		1	1
**Cirrhosis** Decompensated cirrhosis is associated with increased risk for adverse health events as a result of pregnancy (Box 3).												
a. Compensated (normal liver function)	1		1		1		1		1		1	
b. Decompensated (impaired liver function)	1		2		2		3		2		4	
**Liver tumors** Hepatocellular adenoma and malignant liver tumors are associated with increased risk for adverse health events as a result of pregnancy (Box 3).												
a. Benign												
i. Focal nodular hyperplasia	1		2		2		2		2		2	
ii. Hepatocellular adenoma	1		2		2		3		2		4	
b. Malignant (hepatocellular carcinoma)	1		3		3		3		3		4	
**Respiratory Conditions**												
**Cystic fibrosis** This condition is associated with increased risk for adverse health events as a result of pregnancy (Box 3).	1*		1*		1*		2*		1*		1*	
**Hematologic Conditions**												
**Thalassemia**	2		1		1		1		1		1	
**Sickle cell disease** This condition is associated with increased risk for adverse health events as a result of pregnancy (Box 3).	2		1		1		2/3*		1		4	
**Iron-deficiency anemia**	2		1		1		1		1		1	
**Solid Organ Transplantation**												
**Solid organ transplantation** This condition is associated with increased risk for adverse health events as a result of pregnancy (Box 3).	Initiation	Continuation	Initiation	Continuation	—							
a. No graft failure	1	1	1	1	2		2/3*		2		2*	
b. Graft failure	2	1	2	1	2		2/3*		2		4	
**Drug Interactions**												
**Antiretrovirals used for prevention (PrEP) or treatment of HIV infection**												
See the following guidelines for the most up-to-date recommendations on drug-drug interactions between hormonal contraception and antiretrovirals: 1) Recommendations for the Use of Antiretroviral Drugs During Pregnancy and Interventions to Reduce Perinatal HIV Transmission in the United (https://clinicalinfo.hiv.gov/en/guidelines/perinatal/prepregnancy-counseling-childbearing-age-overview?view=full#table-3) (*4*) and 2) Guidelines for the Use of Antiretroviral Agents in Adults and Adolescents with HIV (https://clinicalinfo.hiv.gov/en/guidelines/hiv-clinical-guidelines-adult-and-adolescent-arv/drug-interactions-overview?view=full) (*5*).												
a. Nucleoside reverse transcriptase inhibitors (NRTIs)	Initiation	Continuation	Initiation	Continuation	—							
i. Abacavir (ABC)	1/2*	1*	1/2*	1*	1		1		1		1	
ii. Tenofovir (TDF)	1/2*	1*	1/2*	1*	1		1		1		1	
iii. Zidovudine (AZT)	1/2*	1*	1/2*	1*	1		1		1		1	
iv. Lamivudine (3TC)	1/2*	1*	1/2*	1*	1		1		1		1	
v. Didanosine (DDI)	1/2*	1*	1/2*	1*	1		1		1		1	
vi. Emtricitabine (FTC)	1/2*	1*	1/2*	1*	1		1		1		1	
vii. Stavudine (D4T)	1/2*	1*	1/2*	1*	1		1		1		1	
b. Nonnucleoside reverse transcriptase inhibitors (NNRTIs)												
i. Efavirenz (EFV)	1/2*	1*	1/2*	1*	2*		1*		2*		2*	
ii. Etravirine (ETR)	1/2*	1*	1/2*	1*	1		1		1		1	
iii. Nevirapine (NVP)	1/2*	1*	1/2*	1*	1		1		1		1	
iv. Rilpivirine (RPV)	1/2*	1*	1/2*	1*	1		1		1		1	
c. Ritonavir-boosted protease inhibitors												
i. Ritonavir-boosted atazanavir (ATV/r)	1/2*	1*	1/2*	1*	2*		1*		2*		2*	
ii. Ritonavir-boosted darunavir (DRV/r)	1/2*	1*	1/2*	1*	2*		1*		2*		2*	
iii. Ritonavir-boosted fosamprenavir (FPV/r)	1/2*	1*	1/2*	1*	2*		1*		2*		2*	
iv. Ritonavir-boosted lopinavir (LPV/r)	1/2*	1*	1/2*	1*	1		1		1		1	
v. Ritonavir-boosted saquinavir (SQV/r)	1/2*	1*	1/2*	1*	2*		1*		2*		2*	
vi. Ritonavir-boosted tipranavir (TPV/r)	1/2*	1*	1/2*	1*	2*		1*		2*		2*	
d. Protease inhibitors without ritonavir												
i. Atazanavir (ATV)	1/2*	1*	1/2*	1*	1		1		1		2*	
ii. Fosamprenavir (FPV)	1/2*	1*	1/2*	1*	2*		2*		2*		3*	
iii. Indinavir (IDV)	1/2*	1*	1/2*	1*	1		1		1		1	
iv. Nelfinavir (NFV)	1/2*	1*	1/2*	1*	2*		1*		2*		2*	
e. CCR5 co-receptor antagonists												
i. Maraviroc (MVC)	1/2*	1*	1/2*	1*	1		1		1		1	
f. HIV integrase strand transfer inhibitors												
i. Raltegravir (RAL)	1/2*	1*	1/2*	1*	1		1		1		1	
ii. Dolutegravir (DTG)	1/2*	1*	1/2*	1*	1		1		1		1	
iii. Elvitegravir (EVG)	1/2*	1*	1/2*	1*	1		1		1		1	
g. Fusion inhibitors												
i. Enfuvirtide	1/2*	1*	1/2*	1*	1		1		1		1	
**Anticonvulsant therapy**												
a. Certain anticonvulsants (phenytoin, carbamazepine, barbiturates, primidone, topiramate, and oxcarbazepine)	1		1		2*		1*		3*		3*	
b. Lamotrigine	1		1		1		1		1		3*	
**Antimicrobial therapy**												
a. Broad-spectrum antibiotics	1		1		1		1		1		1	
b. Antifungals	1		1		1		1		1		1	
c. Antiparasitics	1		1		1		1		1		1	
d. Rifampin or rifabutin therapy	1		1		2*		1*		3*		3*	
**Psychotropic medications**												
a. Selective serotonin reuptake inhibitors (SSRIs)	1		1		1		1		1		1	
**St. John’s wort**	1		1		2		1		2		2	

**Abbreviations:** ARV = antiretroviral; BMI = body mass index; CHC = combined hormonal contraceptive; COC = combined oral contraceptive; Cu-IUD = copper intrauterine device; DMPA = depot medroxyprogesterone acetate; DRSP = drospirenone; DVT = deep venous thrombosis; hCG = human chorionic gonadotropin; HDL = high-density lipoprotein; IUD = intrauterine device; LDL = low-density lipoprotein; LNG-IUD = levonorgestrel intrauterine device; NA = not applicable; PE = pulmonary embolism; PID = pelvic inflammatory disease; POP = progestin-only pill; PrEP = pre-exposure prophylaxis; STI = sexually transmitted infection; VTE = venous thromboembolism.

* Consult the appendix for this contraceptive method for a clarification to this classification.
